# Extracting adverse event nature, severity, timelines and resulting interventions from clinical notes of patients receiving CAR-T cell therapy using large language models

**DOI:** 10.1371/journal.pdig.0001426

**Published:** 2026-08-27

**Authors:** Jordan Guillot, Brenda Miao, Arvind Suresh, Madhumita Sushil, Christopher Y. K. Williams, Rohit Vashisht, Tomiko T. Oskotsky, Marina Sirota, Atul J. Butte

**Affiliations:** 1 Bakar Computational Health Sciences Institute, University of California, San Francisco, San Francisco, California, United States of America; 2 Quality Health, Inc., San Francisco, California, United States of America; 3 Division of Clinical Informatics and Digital Transformation, University of California, San Francisco, San Francisco, California, United States of America; Mayo Clinic Rochester: Mayo Clinic Minnesota, UNITED STATES OF AMERICA

## Abstract

Chimeric Antigen Receptor T-cell (CAR‑T) therapy, genetically engineered patient T cells targeting tumor antigens, has transformed care for hematologic malignancies but requires careful tracking of adverse events (AEs) often documented only in unstructured electronic health record (EHR) notes. We evaluated a Large Language Model (LLM)–based approach in UCSF’s secure environment to extract AEs, dates, grades, and interventions within 30 days post‑infusion for six commercial CAR‑T products (2012–2023), benchmarking against two evaluators. Using GPT‑4‑0314 in a zero‑shot setting with four prompts (prespecified AEs, non‑prespecified AEs, CRS, ICANS), we compared outputs against dual annotations on a random sample of 50 notes using accuracy, precision, recall, F1, and Cohen’s kappa. From 4,762 progress notes for 293 patients (median age 65.6), CRS occurred in 80.2% (median onset 4 days); neutropenia 70.0% (16 days); neutropenic fever 64.8% (4 days); ICANS in 34.8%. Interventions included tocilizumab and corticosteroids. Grades were frequently undocumented (CRS 62.3%, ICANS 56.1%); documented cases were mainly CRS grade 1 (59.4%) and ICANS grade 2 (28.0%). Performance was high on CRS and ICANS grading (accuracy of 0.97 and 0.91, respectively). Moderate performances were assessed for prespecified AE extraction (accuracies 0.62–0.76), and non‑prespecified AEs (accuracies 0.76–0.84). Inter‑rater reliability (IRR) was strong for CRS/ICANS presence and grade (kappa 0.86–0.96), moderate for dates and interventions, and weaker for broader AE attributes. LLM‑derived insights can augment AE monitoring and real‑world evidence generation by unlocking unstructured clinical detail and characteristic timelines after CAR T. However, performance varied for broader AE attributes, warranting cautious use. Performance was highest for detecting and grading CRS and ICANS, with strong to near-perfect IRR. While cautious use of LLMs is warranted due to the variable performance observed in this study, these results support further evaluation of supervised CRS/ICANS extraction in controlled EHR-based research or pilot settings, with monitoring and prospective validation.

## Author summary

Chimeric Antigen Receptor T-cell (CAR‑T) therapy has transformed care for blood cancer but requires careful tracking of adverse events (AEs). We asked whether a large language model could read routine clinical notes and extract AEs after CAR T‑cell therapy. We analyzed de‑identified notes from the first month after infusion. The model identified when two key side effects occurred—cytokine release syndrome (a whole‑body inflammatory reaction) and neurotoxicity (brain and nerve symptoms)—and how severe they were, with accuracy similar to human reviewers. It also captured when side effects started and what treatments were given, though performance was more variable for the wider range of side effects beyond these two. In our data, these reactions often arose within the first week; blood count problems and infections were also common. Since many notes did not state severity explicitly, the model sometimes could not assign a grade, and the small manually reviewed sample means results on performance are preliminary. We recommend cautious, supervised use focused on higher-performing tasks (detecting and grading CRS and neurotoxicity), with continued validation before broader deployment in electronic records.

## Introduction

Chimeric Antigen Receptor T-cell (CAR-T) therapy represents a significant advancement in the field of oncology, offering a treatment modality that can induce high response rates and durable remissions in refractory hematologic cancers, with active innovation aimed at expanding targets, improving safety, and overcoming barriers in solid tumors [1–4]. This therapeutic approach involves the genetic modification of a patient’s T-cells to express a CAR that targets specific antigens on tumor cells, thereby enhancing the immune system’s ability to recognize and destroy cancer cells [5]. In 2023, six CAR T-cell therapies were available on the US market for the treatment of hematologic malignancies, including lymphomas, some forms of leukemia, and multiple myeloma [6]. These Food and Drug Administration (FDA)-approved CAR T-cell therapies target one of two antigens on B cells, CD19 or BCMA. Despite their considerable therapeutic potential, CAR-T cell therapies are associated with frequent and clinically significant adverse events (AEs) [1–4], including Cytokine Release Syndrome (CRS) [7–9] and Immune Effector Cell-Associated Neurotoxicity Syndrome (ICANS) [10] which are among the most important toxicities observed after CAR-T therapy.

CRS is a potentially severe and potentially life-threatening AE characterized by an excessive immune response triggered by the activated CAR-T cells releasing large amounts of cytokines into the bloodstream [11]. Symptoms of CRS can range from mild constitutional symptoms to severe inflammatory responses, which can lead to multi-organ dysfunction or even death if not managed promptly and effectively [12]. The severity of CRS is often correlated with the disease burden and treatment response, which complicates the clinical management of affected patients [13]. In addition to CRS, other AEs can be detrimental to patients, including ICANS - a neurologic toxicity of CAR‑T and other immune effector therapies characterized by symptoms such as encephalopathy, confusion, aphasia, tremor, seizures, and, rarely, cerebral edema - that is thought to arise from cytokine‑driven endothelial activation and blood–brain barrier disruption [8,10,14]. Given the serious nature of CRS, ICANS, and other associated AEs, continuous and meticulous monitoring is imperative for the successful implementation of CAR-T therapy, the early detection of AEs, and the optimization of treatment protocols to reduce patient risks and ensure the benefits of CAR-T therapy [15].

Electronic health records (EHRs) provide a rich data source for monitoring outcomes in patients receiving CAR-T therapy; however, much of the clinically relevant information required is difficult to capture from structured data alone. Structured EHR data (diagnosis codes, medication administrations, lab values, procedure codes) are often not sufficiently accurate or detailed. As a result, they rarely capture adverse events with sufficient precision: the timelines, how symptoms change, what interventions occur, and the differences among AE grades. Prior studies have shown that relying exclusively on structured data can lead to underreporting or misclassification of adverse events in oncology cohorts, as coding practices vary across institutions and key clinical details are frequently documented only in clinical notes [16,17]. In contrast, unstructured data—including provider progress notes, nursing documentation, pathology reports, and consult notes—contain the granular qualitative information necessary to identify event onset, progression, and severity. Extracting such information at scale has historically required intensive manual chart review, which is labor-intensive and not feasible for large real-world datasets [18].

The advent of large language models (LLMs), such as the GPT (Generative Pre-trained Transformer) series developed by OpenAI, allows for innovative application of artificial intelligence in various fields, including healthcare [19]. LLMs are a type of artificial intelligence that utilizes deep learning techniques to understand and generate human-like text based on the training received from a vast corpus of text data [20]. These models are not only capable of analyzing context and generating text but also of performing tasks like translation, summarization, and, importantly for healthcare science, information extraction [21]. LLMs can be used to automate and enhance various data-intensive tasks that traditionally require extensive human labor, such as extracting patient information from unstructured clinical notes or assisting in real-time patient monitoring [22–24]. Leveraging natural language processing and large language models enables automated extraction of clinically relevant features from unstructured text, bridging the gap between codified data and nuanced clinical phenomena, and ultimately supporting more accurate AE monitoring in CAR-T populations [25–27].

The aim of this study was to evaluate the capability of a large language model to extract information about AEs, including their dates and associated clinical interventions, and to identify CRS and ICANS grades from the unstructured text of clinical progress notes following CAR-T therapy.

## Methods

**Fig 1:**
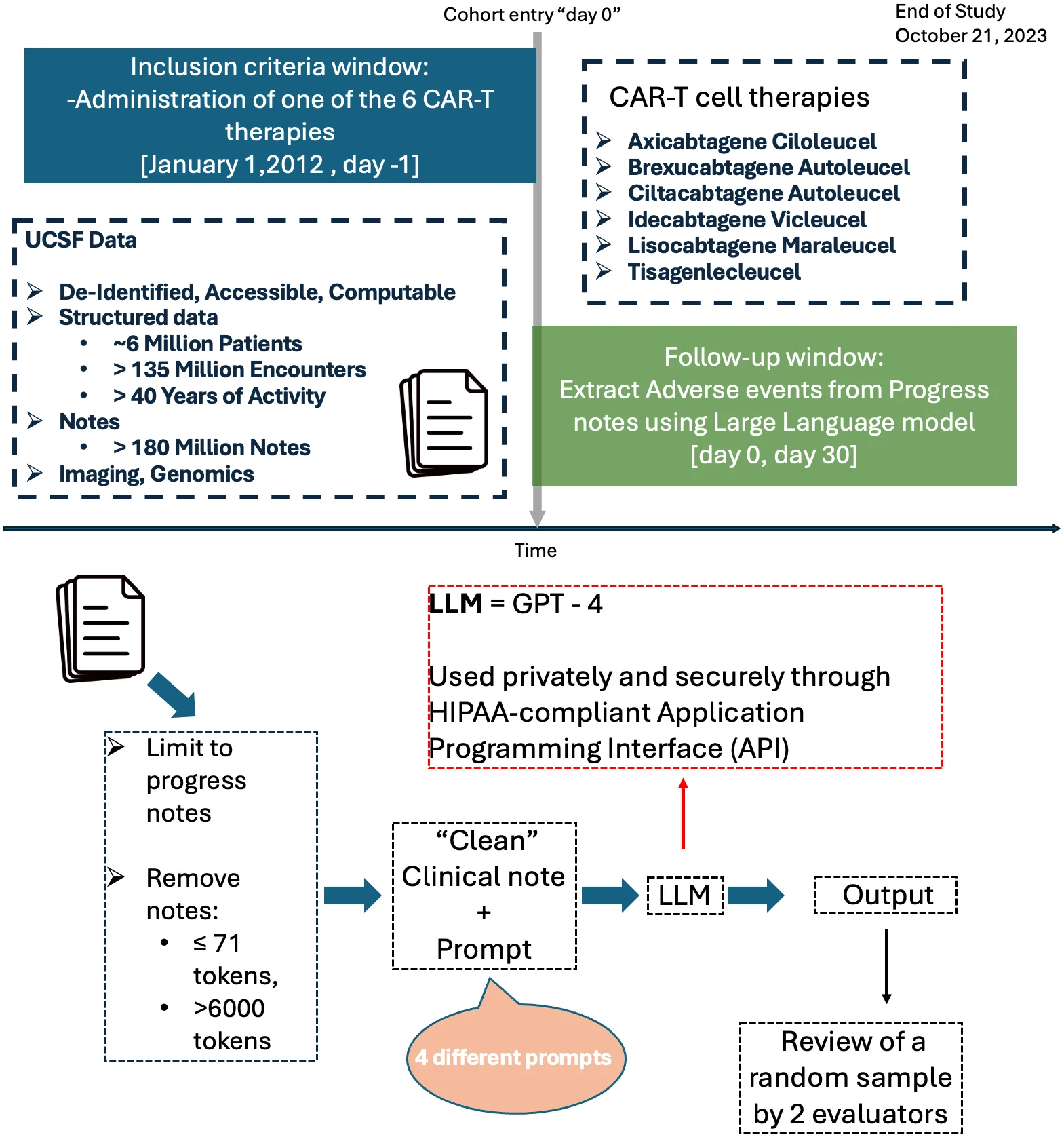
Data sources describing UCSF patients, encounters, and note counts; cohort selection based on CAR‑T therapy administration with follow‑up for adverse event extraction; and study workflow including note filtering, GPT‑4 prompting, and random‑sample annotator review. Data sources and counts. The figure summarizes the electronic health record sources used at University of California, San Francisco (UCSF), including the total number of unique patients, encounters, and clinical notes available for analysis. Patient counts refer to unique individuals; encounter counts refer to distinct inpatient or outpatient visits; note counts refer to individual clinical documents (progress notes, discharge summaries, procedure notes, and related documentation). Cohort selection for chimeric antigen receptor T cell (CAR-T) therapy recipients. The figure shows identification of the study cohort by selecting patients who received CAR-T therapy. The index date is defined as the date of CAR-T administration. The follow up window for adverse event assessment begins at the index date and extends for 30 days after infusion, during which notes are evaluated for adverse events. Study workflow for adverse event extraction. The panel outlines the analytic workflow applied to clinical notes from the final cohort: (1) note filtering by time window relative to CAR T infusion and by note type (progress notes only); (2) large language model (LLM) prompting using Generative Pretrained Transformer 4 (GPT 4) to extract adverse event; and (3) random sample evaluator review to assess model performance. Abbreviations: UCSF, University of California, San Francisco; CAR-T, chimeric antigen receptor T cell; AE, adverse event; EHR, electronic health record; LLM, large language model; GPT 4, Generative Pretrained Transformer 4.

In this cross-sectional study, we identified all patients visiting the University of California, San Francisco (UCSF) hospital between January 1, 2012 and October 21, 2023 and receiving any of the six following CAR-T cell therapies: axicabtagene ciloleucel, idecabtagene vicleucel, ciltacabtagene autoleucel, lisocabtagene maraleucel, tisagenlecleucel, and brexucabtagene autoleucel (Fig 1). From reference papers and expert opinions we identified a list of 25 adverse events that are recognized as CAR-T adverse events [6–8,14]. We defined these as the prespecified adverse events, which included: “Cytokine Release Syndrome (CRS)”, “Immune Effector Cell-Associated Neurotoxicity Syndrome (ICANS)”, “Hypogammaglobulinemia”, “Neutropenic Fever”, “Macrophage Activation Syndrome (MAS)”, “Hemophagocytic Lymphohistiocytosis (HLH)”, “Tumor Lysis Syndrome”, “Movement disorder”, “Cognitive impairment”, “Personality changes”, “B-cell aplasia”, “thrombocytopenia”, “anemia”, “neutropenia”, “leukopenia”, “CD4 lymphopenia”, “Cranial Nerve Palsy”, “Bacterial infection”, “Viral Infection”, “Zoster Infection”, “Fungal infection”, “Aspergillus infection”, “Pneumocystis Jirovecii Pneumonia infection”, “Secondary myeloid malignancies”, “Graft-versus-host disease (GVHD)”.

We retrieved all clinical notes from this cohort. We extracted all progress notes that occurred in the 30 days following CAR-T therapy infusion. If a patient received multiple CAR-T infusions at UCSF, each infusion was counted as a separate, independent episode. However, the primary description of the cohort and AE frequencies were aggregated at the patient level. These clinical notes have been previously de-identified, externally certified, and made available for research as previously described [28]. We tokenized all candidate notes using tiktoken and compared token counts to the GPT 4 0314 context window (8k tokens). To ensure the full note plus prompt (described below) fit and allow headroom for model output, we set an operational cap of 6,000 input tokens per note. Notes with fewer than 71 tokens (bottom quartile) were excluded due to limited clinical content, and notes with more than 6,000 tokens were excluded to avoid exceeding model context. No truncation or chunking was performed in the primary analysis; each retained note was submitted in full.

We restricted the analysis to progress notes because these documents consistently synthesize interval symptoms, assessment/plans, and bedside interventions relevant to AE onset, grade rationales, and management. To reduce heterogeneity in narrative structure and to ensure cost-effective analysis and evaluation, we excluded other types of notes (e.g., nursing documentation, consult notes, procedure notes, imaging reports, discharge summaries). This design choice prioritized meaningful clinical extraction for zero-shot prompting.

Accessing the application programming interface (API) via the Health Insurance Portability and Accountability Act (HIPAA)–compliant UCSF Secure Azure OpenAI environment, we utilized the GPT-4 model (OpenAI model = “gpt-4-0314”; role = “user”; temperature = 0; all other settings at default values). For each retained note, we constructed the user message as ‘Clinical note: “”“”“”’ + task and computed a conservative output-token budget to keep the combined input+output within the 8,192-token context window. Because the 6,000-token input cap was enforced upstream with tiktoken, input side overflows were not expected. If the code returned a token-budget error, we reduced the max tokens parameter and retried. We used four distinct prompts in a zero-shot manner (S1 Table), i.e., providing no additional training context, examples, or guidelines to the model. The first prompt asked the model to return prespecified AEs expected after any CAR-T infusion along with their dates and any associated clinical interventions documented within the clinical notes. The second prompt asked the model to detect the presence of any AEs related to CAR-T that are not among the prespecified AEs along with their dates and any clinical interventions. Third, we prompted the LLM to identify the presence, date, and grade of CRS along with any associated clinical interventions. Lastly, we prompted the LLM to identify the presence, date, and grade of ICANS, and any associated clinical interventions. Clinicians frequently document ‘CRS grade 0’ and ‘ICANS grade 0’ in the clinical notes during daily monitoring to indicate no active toxicity. We therefore instructed the model to extract ‘grade 0’ when explicitly documented, even though it reflects absence of the event. In all analyses, grade 0 was treated as an explicitly documented grade category and interpreted as absence of active CRS/ICANS, rather than as an adverse event. Each of the four prompts was executed independently on the same set of progress notes. AE and Clinical interventions counts were derived separately per prompt without cross‑prompt reconciliation. Therefore, AE and clinical intervention counts are prompt‑specific and may differ across prompts.

A sample of notes was randomly selected for manual evaluation of the LLM performance. It was designed as a feasibility evaluation to assess whether a zero‑shot LLM could recover key adverse event elements from routine notes. We selected 50 progress notes at random from the eligible set. These sampled notes represented 47 different patients and enabled independent double evaluation by two clinical evaluators within practical time constraints. While each note generated multiple element-level assessments across the four prompts (AE presence, date, grade for CRS/ICANS, and interventions), these observations were not fully independent, and therefore do not eliminate the uncertainty associated with the modest note-level sample size. Our intent was not to produce narrow confidence bounds but to obtain point estimates and to identify tasks where model behavior appeared reliable enough at the zero-shot step. No prompt engineering was performed on the validation notes. LLM outputs were manually reviewed and evaluated as True or False for AEs, grades (for the 3rd and 4th prompts), dates of occurrence, and associated clinical interventions by Jordan Guillot (JG): a PharmD, PhD, with 5 years of postgraduate training in pharmacoepidemiology research and Arvind Suresh (AS): an MD, an oncologist with 3 years of postgraduate general medical training, to compare the performance between the LLM models and human annotations. We evaluated performance separately for each element: AE presence, grade, AE date, and clinical intervention. For each element, a true positive was counted when the system correctly extracted the element as documented in the note. A false positive was counted when the system extracted an element that was not documented or extracted an incorrect element. A true negative was counted when the element was not documented in the note and the LLM made no extraction. A false negative was counted when the element was documented but the LLM failed to extract it. An annotation guide was written by JG and provided to AS to ensure that both evaluators followed a similar evaluation process (S1 Annotation Guide). After reviewing the guide, the reviewers independently assessed the veracity of the model outputs. The reviewers remained independent and did not share information or formally resolve disagreements for the IRR analysis, because the goal was to quantify independent agreement rather than generate consensus labels. Performance was evaluated using accuracy, precision, recall, and F1 score, computed using their standard definitions [29]. The average performance was calculated by macro-averaging the results from the two evaluators. Inter-rater reliability (IRR) between the two reviewers was assessed by Cohen’s kappa [30]. Among the prespecified adverse events, four could be identified from either or both free text or blood count laboratory values: anemia, thrombocytopenia, neutropenia, and leukopenia. Given the complexity and variability in how these are reported in clinical notes, we conducted a sensitivity analysis and calculated IRR for the prespecified adverse events after excluding these four.

This study utilized data drawn from the UCSF Health system, where deidentification of the data has been completed to enable clinical research projects in accordance with guidance from UCSF institutional review boards (IRBs), privacy, and compliance standards. The UCSF IRBs determined that this use of de-identified structured and clinical text data in the UCSF Information Commons is not considered human participant research and that the study was therefore exempt from further approval and the need for informed consent (Fig 1).

## Results

A total of 293 patients receiving CAR-T cell therapy at UCSF were included in the study. Among them, 97 (33.1%) patients received Axicabtagene ciloleucel, 64 (21.8%) received Ciltacabtagene autoleucel, 43 (14.7%) received Tisagenlecleucel, 37 (12.6%) received Lisocabtagene maraleucel, 36 (12.3%) received Idecabtagene vicleucel and 16 (5.4%) received Brexucabtagene autoleucel. Among these patients, only one received CAR-T therapy twice, in two separate instances approximately 10 months apart. Due to the rarity of this situation and the time difference between instances, we expect any impact on the analysis descriptive findings to be minimal. Median age was 65.6 years [IQR: 54.8 – 71.7]. There were 176 (60.0%) male and 117 (40.0%) female. Among all patients 170 (58.0%) were White, 69 (23.5%) Hispanic, 34 (11.6%) Asian, 11 (3.8%) Black and ≤ 10 (≤3.4%) other than these race/ethnicity categories. Among these patients, the most common disease codes in UCSF EHR that matched the approved indications for CAR-T therapy were diffuse large B-cell lymphoma (DLBCL), coded for 150 patients (51.2%); multiple myeloma, for 131 patients (44.7%); and acute lymphoblastic leukemia (ALL), for 36 patients (12.3%). Among included patients, 25,974 clinical notes documented within 30 days after CAR‑T administration were identified. Specifically, after filtering by note length (i.e., retaining only notes with more than 71 tokens and fewer than 6000 tokens), 18,808 notes remained, of which 4,762 (25.3%) were progress notes retained for analysis (Table 1). The other types of notes not retained for the analysis were 2503 (13.3%) imaging notes, 2212 (11.8%) consults, 1986 (10.6%) plans of care, 1475 (7.8%) registered nurse notes, 1359 (7.2%) interdisciplinary notes, 588 (3.1%) procedure notes and 3923 (20.9%) other type of notes (S2 Table).

**Table 1:** Description of the patients visiting the University of California, San Francisco (UCSF) hospital between January 1,2012 and October 21, 2023 and receiving any of the 6 following CAR-T cell therapies: axicabtagene ciloleucel, idecabtagene vicleucel, ciltacabtagene autoleucel, lisocabtagene maraleucel, tisagenlecleucel, brexucabtagene autoleucel.

Variables	Cohort	Axicabtagene ciloleucel	Ciltacabtagene autoleucel	Lisocabtagene maraleucel	Idecabtagene vicleucel	Tisagenlecleucel	Brexucabtagene autoleucel
	n = 293	n = 97	n = 64	n = 37	n = 36	n = 43	n = 16
Age – Median [Q1 – Q3]	65.6 [54.8 – 71.7]	64.1 [53.9 – 69.0]	66.9 [63.6 – 71.5]	74.5 [64.0 – 75.9]	69.2 [62.6 – 73.9]	20.1 [11.0 -58.1]	64.0 [48.4 – 72.9]
Sex – n (%)							
Male	176 (60.0)	66 (68.0)	38 (59.4)	22 (56.4)	21 (58.3)	23 (52.2)	12 (75.0)
Female	117 (40.0)	31(32.0)	26 (40.6)	17 (43.6)	15 (41.7)	21 (47.8)	≤10 (≤62.5)
Race/Ethnicity – n (%)							
White	170 (58.0)	55 (56.7)	48 (75.0)	21 (53.8)	26 (72.2)	17 (38.6)	≤10 (≤62.5)
Hispanic	69 (23.5)	26 (26.8)	≤10 (≤15.6)	≤10 (≤27.0)	≤10 (≤27.8)	20 (45.5)	≤10 (≤62.5)
Asian	34 (11.6)	≤10 (≤ 10.3)	≤10 (≤15.6)	≤10 (≤27.0)	≤10 (≤27.8)	≤10 (≤23.3)	–
Black	11 (3.8)	≤10 (≤10.3)	≤10 (≤15.6)	≤10 (≤27.0)	≤10 (≤27.8)	≤10 (≤23.3)	≤10 (≤62.5)
Other	≤10 (≤3.4)	≤10 (≤10.3)	≤10 (≤15.6)	≤10 (≤27.0)	–	≤10 (≤23.3)	≤10 (≤62.5)
CAR-T cell infused – n (%)							
Axicabtagene ciloleucel	97 (33.1)	97 (100)	–	–	–	–	–
Ciltacabtagene autoleucel	64 (21.8)	–	64 (100)	–	–	–	–
Lisocabtagene maraleucel	37 (12.6)	–	–	37 (100)	–	–	–
Idecabtagene vicleucel	36 (12.3)	–	–	–	36 (100)	–	–
TisagenlecleuceL	43 (14.7)	–	–	–	–	43 (100)	–
Brexucabtagene autoleucel	16 (5.5)	–	–	–	–	–	16 (100)
Diagnosis related to CAR-T – n (%)							
Acute Myeloid Leukemia (AML)	13 (4.4)	≤10 (≤10.3)	≤10 (≤15.6)	–	≤10 (≤27.8)	≤10 (≤23.3)	≤10 (≤62.5)
Acute lymphoblastic leukemia (ALL)	36 (12.3)	≤10 (≤10.3)	–	–	–	29 (67.4)	≤10 (≤62.5)
Chronic Lymphocytic Leukemia (CLL)	≤10 (≤3.4)	≤10 (≤10.3)	–	–	–	≤10 (≤23.3)	–
Diffuse Large B-cell Lymphoma (DLBCL)	150 (51.2)	96 (99.0)	–	37 (100)	≤10 (≤27.8)	13 (30.2)	≤10 (≤62.5)
Follicular Lymphoma	26 (8.9)	17 (17.5)	–	≤10 (≤27.0)	–	≤10 (≤23.3)	–
Mantle cell lymphoma (MCL)	12 (4.1)	≤10 (≤10.3)	–	≤10 (≤27.0)	–	–	≤ 10 (≤62.5)
Multiple Myeloma	131 (44.7)	20 (20.6)	64 (100)	≤10 (≤27.0)	36 (100)	≤10 (≤23.3)	≤ 10 (≤62.5)
Clinical notes available							
Total - n	25974	9519	4817	2914	2596	4470	1658
Length filtered Progress notes - n	4762	1651	1118	549	526	601	317

**Fig 2:**
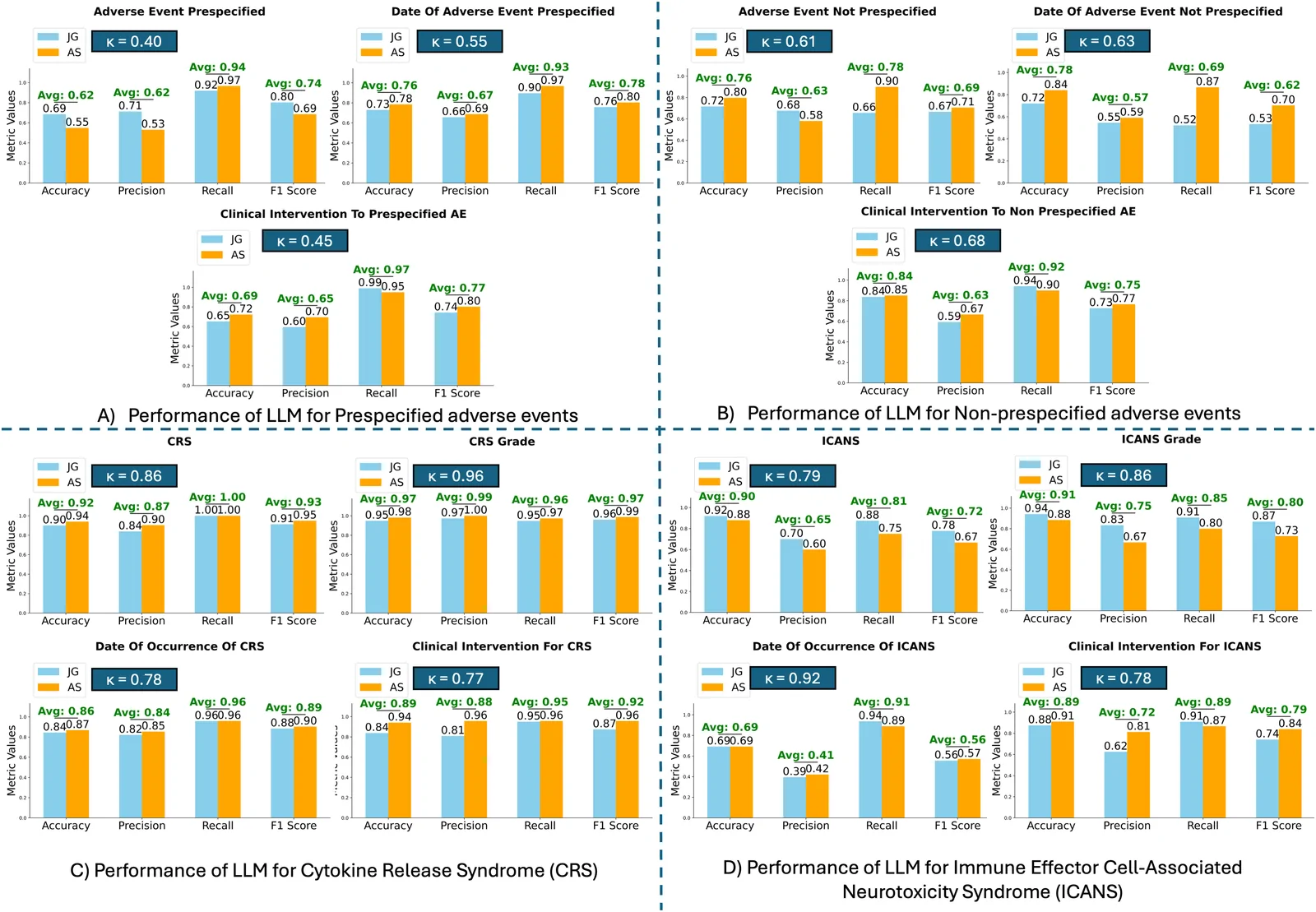
Performance of the LLM in extracting adverse event presence, grade when applicable, date, and clinical interventions from CAR-T clinical notes across four zero-shot prompts: (A) prespecified AEs, (B) non-prespecified AEs, (C) Cytokine Release Syndrome (CRS), and (D) Immune Effector Cell-Associated Neurotoxicity Syndrome (ICANS). Performance metrics include accuracy, precision, recall, and F1 score. Blue bars represent results from reviewer Jordan Guillot (JG), and orange bars represent results from reviewer Arvind Suresh (AS). Green text above each metric indicates the average value across evaluators. The blue box in each panel displays Cohen’s kappa value, representing inter-reviewer agreement. Abbreviations: adverse event, AE; cytokine release syndrome, CRS; immune effector cell-associated neurotoxicity syndrome, ICANS; F1 score, harmonic mean of precision and recall; Cohen’s kappa, measure of inter-reviewer agreement.

Point estimates for performance in our evaluation sample varied across the four prompts (Fig 2, S3 Table). Averaging across the two evaluators, prespecified AEs showed mean accuracies of 0.62 (nature), 0.76 (date), and 0.69 (clinical intervention), with average F1 scores ranging from 0.74 (nature) to 0.78 (date). For non‑prespecified AEs, average accuracies were 0.76 (nature), 0.78 (date), and 0.84 (clinical intervention), with average F1 scores from 0.62 (date) to 0.75 (clinical intervention). For CRS-specific prompts, point estimates were consistently high: average accuracies ranged from 0.86 (date) to 0.97 (grade), with average F1 scores from 0.89 (date) to 0.97 (grade). For ICANS-specific prompts, average accuracies ranged from 0.69 (date) to 0.91 (grade), with average F1 scores from 0.56 (date) to 0.80 (grade).

Inter-rater reliability (IRR), assessed using Cohen’s kappa, between evaluators varied by output category, using the bands weak (0.40–0.60), moderate (0.60–0.80), strong (0.80–0.90), and near-perfect (>0.90). For prespecified AEs, IRR was weak (nature 0.40; date 0.55; clinical intervention 0.45). For non‑prespecified AEs, IRR was moderate (nature 0.61; date 0.63; clinical intervention 0.68). For CRS prompts, IRR was strong for presence (0.86), near-perfect for grade (0.96), and moderate for date (0.78) and clinical intervention (0.77). For ICANS prompts, IRR was moderate for presence (0.79) and clinical intervention (0.78), strong for grade (0.86), and near-perfect for date (0.92) (Fig 2). Of the 264 inter-reviewer discrepancies, 16 (6.1%) involved thrombocytopenia, 14 (5.3%) involved anemia within the prespecified AE prompt. False negative model outputs — defined as instances in which the presence of an AE, date, or clinical intervention was identified by an evaluator but not the LLM — were also common discrepancies (3.8% each for AE presence and date in the non-prespecified event prompt; 2.3% each for AE presence and date in the prespecified event prompt). Disagreements on CRS/ICANS presence were infrequent (CRS yes: 1.5%; ICANS yes: 1.1%), consistent with the stronger kappas and higher point estimates we observed for these tasks. Date-related disagreements included many single-date mismatches at low frequency. The sensitivity analysis excluding lab-based AEs (anemia, thrombocytopenia, neutropenia, leukopenia) in the prespecified event prompt showed a moderate IRR for nature (0.63) and lower agreement for date (0.56) and clinical intervention (0.51).

**Fig 3:**
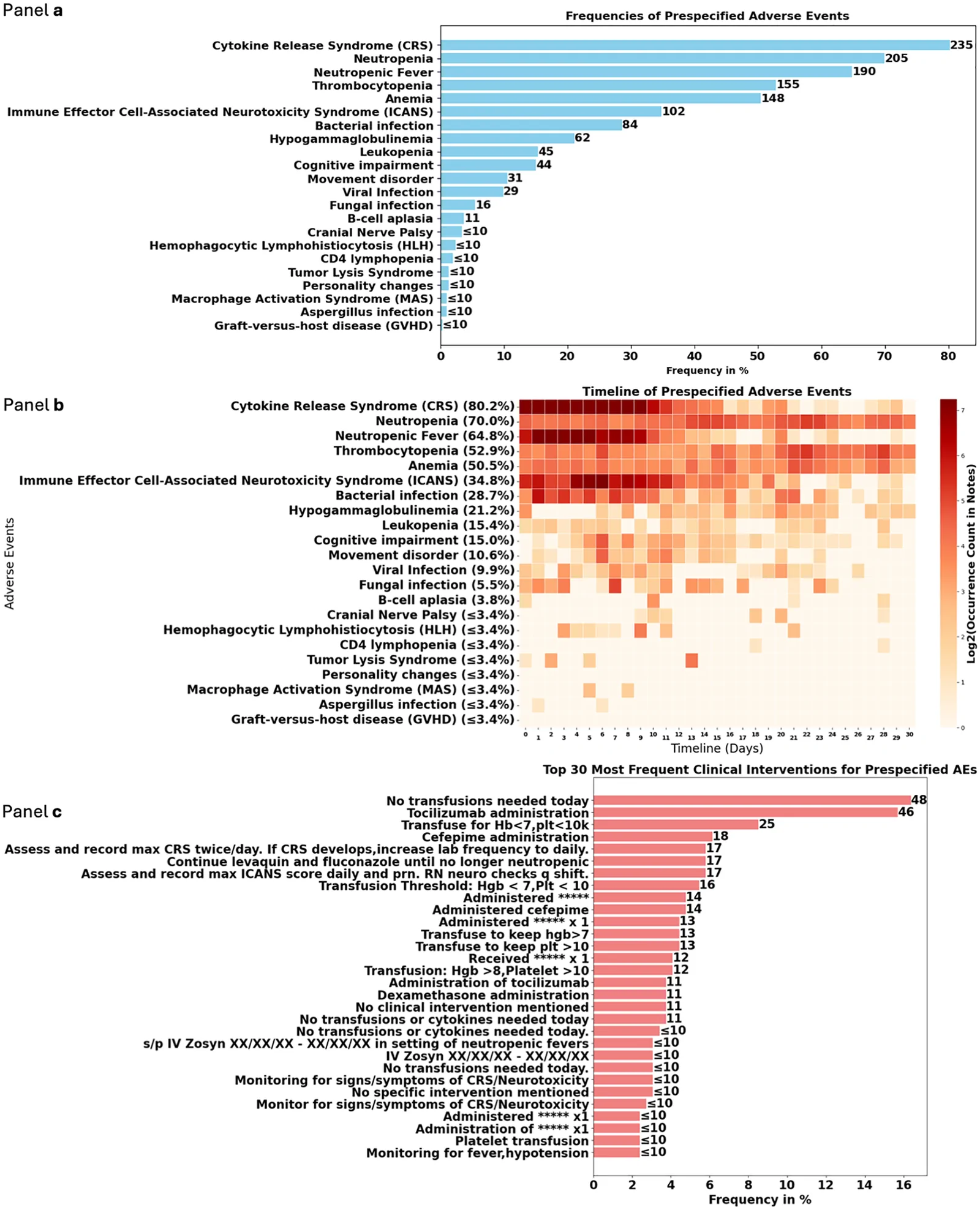
Frequencies and timelines of all identified prespecified adverse events for our cohort (n = 293 patients), with frequencies of the 30 most common resulting clinical interventions. This figure presents the frequencies and timelines of prespecified adverse events (AEs) observed in a cohort of 293 patients, alongside the most common clinical interventions implemented in response. Panel a) Displays a horizontal bar chart illustrating the frequencies of prespecified adverse events as a percentage of the total cohort. The adverse events are ranked from most to least frequent. Each bar represents the percentage of patients experiencing the respective adverse event. Panel b) Shows a heatmap depicting the timeline of prespecified adverse events over the 30-day follow-up time window. The x-axis represents the timeline in days, while the y-axis lists the adverse events and their frequencies. The color intensity corresponds to the log2-transformed occurrence count in clinical notes, with darker red indicating higher frequencies. Panel c) Features a horizontal bar chart of the 30 most frequent clinical interventions for managing the prespecified adverse events. The interventions are ranked by frequency. Each bar represents the frequency patients with the intervention as a percentage of total patient number. Abbreviations used in the figure: CRS: Cytokine Release Syndrome, ICANS: Immune Effector Cell-Associated Neurotoxicity Syndrome, HLH: Hemophagocytic Lymphohistiocytosis, MAS: Macrophage Activation Syndrome, GVHD: Graft-versus-host disease.

The most common prespecified AEs among all patients included CRS, observed in 235 patients (80.2%), with a median onset of 4 days (IQR: 2–7); neutropenia, affecting 205 patients (70.0%), with a median onset at 16 days (IQR: 9–22); and neutropenic fever, reported in 190 patients (64.8%), with a median onset of 4 days (IQR: 2–7). Other hematologic toxicities, such as thrombocytopenia (155, 52.9%) and anemia (148, 50.5%) were also common and had median onset timelines of 19 days (IQR: 8–24) and 15 days (IQR: 6–22), respectively. Infectious complications were also prevalent, with bacterial infections documented in 84 patients (28.7%) and viral infections in 29 patients (9.9%), both occurring at a median timeline of 7–9 days post-treatment. ICANS was reported in 102 (34.8%) patients (Fig 3, Table 2). The most common information on clinical interventions for prespecified AEs extracted by the LLM included “no transfusions needed today” (reported for 48 patients, 16.4%), tocilizumab administration (46, 15.7%), and “Transfuse for Hb<7,plt<10k” (25, 8.5%) (Fig 3, S4 Table).

**Table 2:** Frequencies and timeline of prespecified adverse events.

Adverse event	Frequency	%	Median timeline	Min	Q1	Q3	Max
Cytokine Release Syndrome (CRS)	235	80.2	4	0	2	7	29
Neutropenia	205	70.0	16	0	9	22	30
Neutropenic Fever	190	64.8	4	0	2	7	29
Thrombocytopenia	155	52.9	19	0	8	24	30
Anemia	148	50.5	15	0	6	22	30
Immune Effector Cell-Associated Neurotoxicity Syndrome (ICANS)	102	34.8	6	0	4	9	29
Bacterial infection	84	28.7	7	0	3	11	29
Hypogammaglobulinemia	62	21.2	16.5	0	11	22	30
Leukopenia	45	15.4	12	0	5	21	28
Cognitive impairment	44	15.0	10	0	6	15	29
Movement disorder	31	10.6	10	1	6	14	30
Viral Infection	29	9.9	9	0	6	19	28
Fungal infection	16	5.5	7	0	3	14	24
B-cell aplasia	11	3.8	10	0	10	21	29
Cranial Nerve Palsy	≤10	≤3.4	18	2	10	20	29
Hemophagocytic Lymphohistiocytosis (HLH)	≤10	≤3.4	9	1	4.25	9.75	21
CD4 lymphopenia	≤10	≤3.4	21	12	18	27.5	28
Tumor Lysis Syndrome	≤10	≤3.4	13	0	2	13	18
Personality changes	≤10	≤3.4	21	4	13	22	25
Macrophage Activation Syndrome (MAS)	≤10	≤3.4	6.5	0	5	8	13
Aspergillus infection	≤10	≤3.4	6	1	2.25	17.25	23
Graft-versus-host disease (GVHD)	≤10	≤3.4	28	28	28	28	28

**Fig 4:**
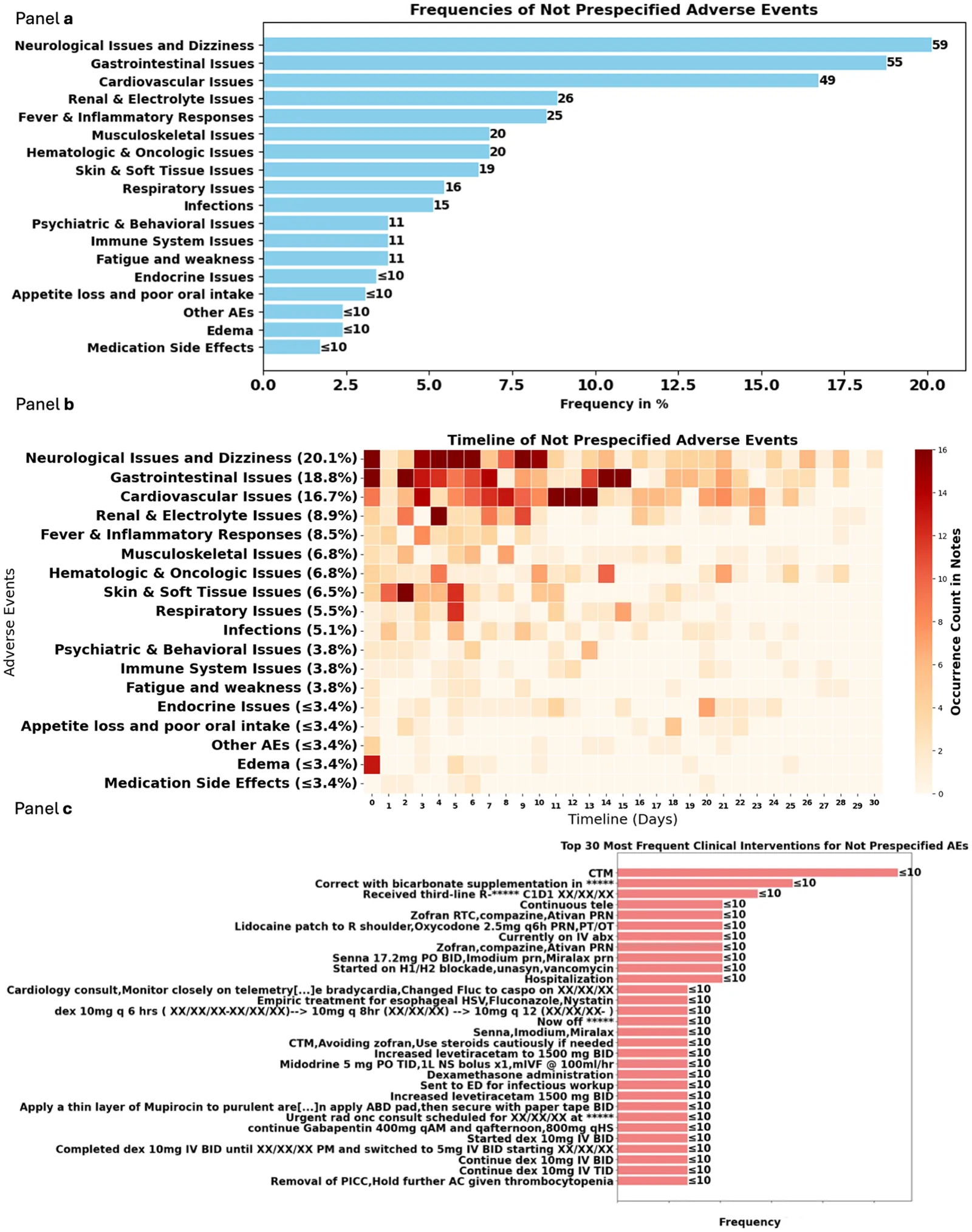
Frequencies and timelines of non-prespecified adverse events for our cohort (n = 293 patients) categorized by medical categories. This figure presents the frequencies and timelines of non-prespecified adverse events (AEs) observed in a cohort of 293 patients, alongside the most common clinical interventions implemented in response. Panel a) Displays a horizontal bar chart illustrating the frequencies of non-prespecified adverse events grouped by medical categories as a percentage of the total cohort. The categories are ranked from most to least frequent. Each bar represents the percentage of patients experiencing the respective adverse event. Panel b) Shows a heatmap depicting the timeline of non-prespecified adverse events grouped by medical categories over the 30-day follow-up time window. The x-axis represents the timeline in days, while the y-axis lists the adverse events grouped by medical categories and their frequencies. The color intensity corresponds to the occurrence count in clinical notes, with darker red indicating higher frequencies. Panel c) Features a horizontal bar chart of the 30 most frequent clinical interventions for managing the non-prespecified adverse events. The interventions are ranked by frequency. Each bar represents the frequency of patients with the intervention. Abbreviations used in the figure: CTM: continuous telemetry monitoring, R:Right, XX/XX/XX: dates were masked, RTC: Return to Clinic, PRN: As needed (“pro re nata”), q6h: Every 6 hours, PT/OT: Physical Therapy/Occupational Therapy, PO: By mouth (orally), BID: Twice a day, IM: Intramuscular, prn: As needed, H1/H2 blockade: Histamine-1/Histamine-2 receptor blockade, IV: Intravenous, ABD: Abdomen, q12h: Every 12 hours, q8h: Every 8 hours, qAM: Every morning, qHS: Every night at bedtime, TID: Three times a day, AC: Before meals (“ante cibum”), PICC: Peripherally Inserted Central Catheter.

Non-prespecified AEs were less frequent than prespecified AEs and more heterogeneous in type because not classified as categories. The most prevalent included fever (4.1%), nausea (3.8%), hypotension (≤3.4%), and constipation (≤3.4%), with median onset timelines ranging from 4 to 7.5 days post-CAR-T therapy. Due to the heterogeneity of non-prespecified AEs extracted, we grouped them for descriptive purposes (S5 Table). When grouped by medical domains, The most prevalent included neurological issues and dizziness (20.1%), gastrointestinal Issues (18.8%), and cardiovascular issues (16.7%), although they might be symptoms of prespecified AEs that would have been wrongly extracted in the prompt for non-prespecified AEs (Fig 4, Table 3). Clinical interventions for non-prespecified AEs were also heterogeneous and included continuous telemetry monitoring (CTM) (≤3.4%) and bicarbonate supplementation (≤3.4%) (Fig 4, S6 Table).

**Table 3:** Frequencies and timelines of the 50 most frequently extracted single non-prespecified adverse events.

Adverse event	Frequency	%	Median timeline	Min	Q1	Q3	Max
Fever	12	4.1	4	0	1.5	5.75	9
Nausea	11	3.8	6	0	1	15.5	19
Hypotension	≤10	≤3.4	7.5	3	4.5	12.5	22
Constipation	≤10	≤3.4	4	0	3	14.5	24
Acute encephalopathy	≤10	≤3.4	6	3	5.25	10	23
Fatigue	≤10	≤3.4	6	0	2	13.5	28
Neurotoxicity	≤10	≤3.4	5.5	4	5	6	6
Rash	≤10	≤3.4	5	1	3.5	9.5	11
Orthostatic hypotension	≤10	≤3.4	10	5	7	14	24
Hypertension	≤10	≤3.4	17.5	5	14.25	22	23
Anxiety	≤10	≤3.4	2.5	0	0.75	5.25	11
Dizziness	≤10	≤3.4	14	3	5	28	30
Hyponatremia	≤10	≤3.4	2	2	2	4.5	8
Headache	≤10	≤3.4	0	0	0	0	18
Hypoxia	≤10	≤3.4	5	5	5	5.5	16
AKI	≤10	≤3.4	9	7	9	9	13
Abdominal pain	≤10	≤3.4	6	5	5	11.5	13
Acute kidney injury	≤10	≤3.4	0	0	0	1	7
Nausea and vomiting	≤10	≤3.4	6	0	5.25	6	14
Pancytopenia	≤10	≤3.4	25	11	18	26.5	28
Immunocompromised	≤10	≤3.4	20	2	12.5	23	27
Adrenal insufficiency	≤10	≤3.4	20	5	15	20	20
Altered mental status	≤10	≤3.4	19	6	18	19	20
Confusion	≤10	≤3.4	3	2	2.5	4	5
Febrile	≤10	≤3.4	5	3	4	6	7
Diarrhea	≤10	≤3.4	15	3	15	15	15
Cytopenias	≤10	≤3.4	13	7	10	20	27
Urinary retention	≤10	≤3.4	20	10	15	20.5	21
Tachycardia	≤10	≤3.4	13	1	12	13	13
Transaminitis	≤10	≤3.4	10	3	7	10	10
Delirium	≤10	≤3.4	5.5	5	5	6	6
Sepsis	≤10	≤3.4	19	5	12	19	19
Malnutrition	≤10	≤3.4	4	3	3.5	8.5	13
Neuropathy	≤10	≤3.4	3	3	3	6.5	10
Elevated LFTs	≤10	≤3.4	14	7	7	14	14
AKI on CKD	≤10	≤3.4	9	5	7	9	9
Syncope	≤10	≤3.4	3	3	3	3	9
Weakness	≤10	≤3.4	7	5	6	8	9
Prolonged QTC	≤10	≤3.4	11	10	10.5	11.5	12
Hypoglycemia	≤10	≤3.4	21	0	10.5	21	21
Bradycardia	≤10	≤3.4	12	5	12	12	12
Immunocompromised State	≤10	≤3.4	3	1	2	4	5
Watery diarrhea	≤10	≤3.4	2	2	2	7	12
Hand tremor	≤10	≤3.4	5	0	2.5	7.5	10
Afib with RVR	≤10	≤3.4	0	0	0	5.25	21
Dizziness with ambulation	≤10	≤3.4	6.5	0	3.25	9.75	13
CKD	≤10	≤3.4	12.5	2	7.25	17.75	23
Neoplasm/malignancy associated pain	≤10	≤3.4	26.5	25	25.75	27.25	28
Somnolence	≤10	≤3.4	8	6	7	9	10
Insomnia	≤10	≤3.4	15.5	11	13.25	17.75	20

**Fig 5:**
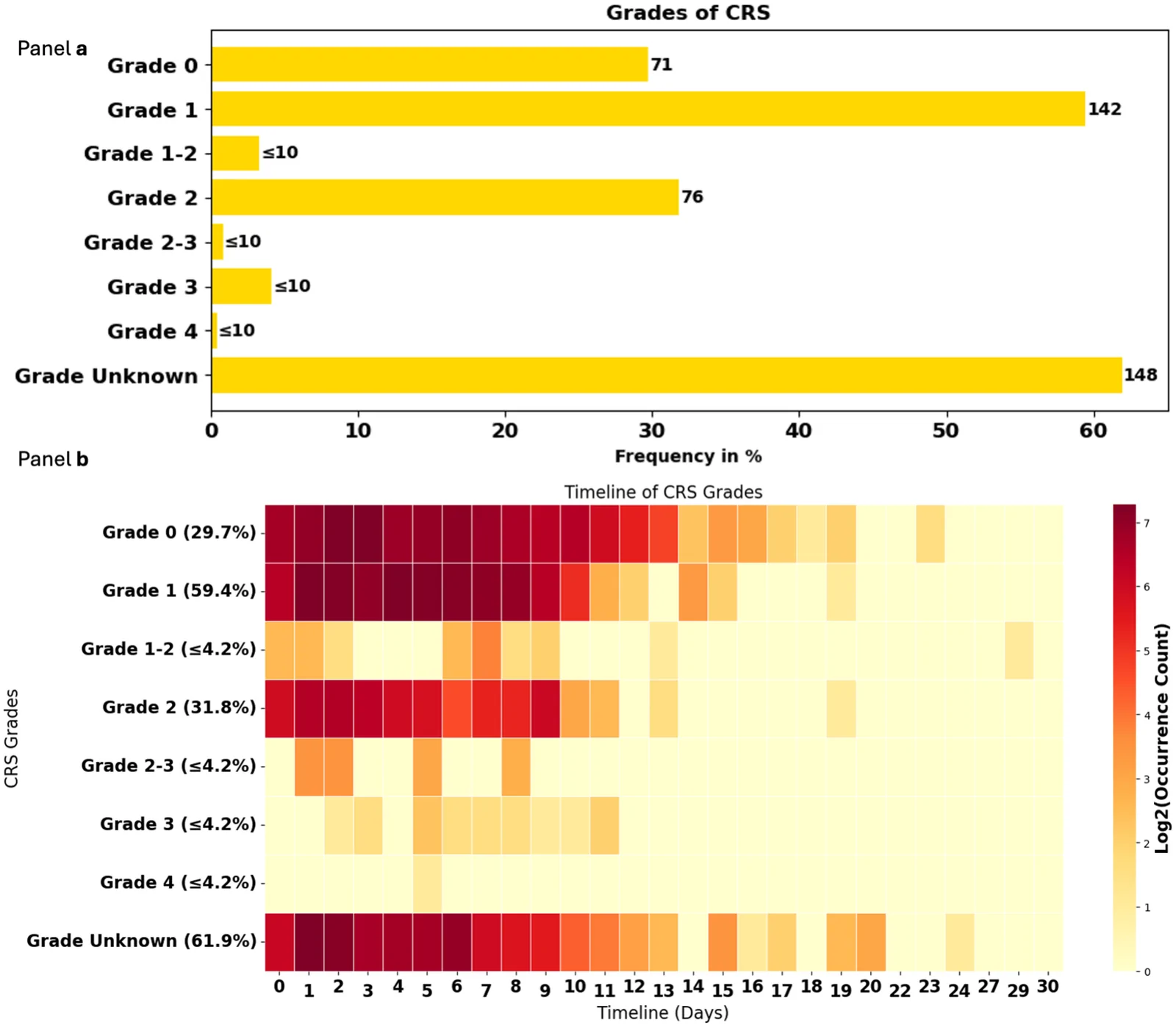
CRS grades: frequencies (bar chart) and timelines (heat map) for our patients experiencing CRS (n = 239 patients), identified using a grade-focused prompt during CRS event detection. This figure presents the frequencies and timelines of occurrence of Cytokine Release Syndrome (CRS) grades in the 239 patients with CRS from our cohort of 293 patients. Panel a) Displays a horizontal bar chart illustrating the frequencies of CRS grades as a percentage of the total patients identified with CRS. The grades are ranked from least to most severe. Panel b) Shows a heatmap depicting the timeline of CRS grades over the 30-day follow-up time window. The x-axis represents the timeline in days, while the y-axis lists the CRS grades and their frequencies. The color intensity corresponds to the log2-transformed occurrence count in clinical notes, with darker red indicating higher frequencies. Abbreviations used in the figure: CRS: Cytokine Release Syndrome.

The prompt aimed at extracting the presence of CRS and its grade identified that CRS impacted 239 (81.6%) of patients. Because each prompt was run independently and outputs were not reconciled across prompts, the patient-level CRS count from the CRS-specific prompt differed from the CRS count extracted by the prespecified AE prompt. Among those, Grade 1 CRS was observed in 142 patients (59.4%), making it the most common grade reported. Grade 0 CRS occurred in 71 patients (29.7%), Grade 1–2 CRS in ≤10 patients (≤4.2%), Grade 2 CRS in 76 patients (31.8%), and Grade 2–3 CRS in ≤10 patients (≤4.2%). Grade 3 CRS in ≤10 patients (≤4.2%), and Grade 4 CRS in ≤10 patients (≤4.2%). CRS grade was identified as unknown for 148 patients (61.9%), highlighting limitations in information available in clinical notes, e.g., physician mentioning CRS without stating the grades even though they might give characteristics of the CRS. Because multiple CRS grades could be documented for the same patient across notes or time points, the grade-specific percentages are not mutually exclusive and should not be summed to 100%; rather, they reflect how frequently each grade was identified overall. (Fig 5, Table 3). The timeline for CRS onset varied across grades, with median onset days ranging from 2 to 7 days post-CAR-T therapy. Patients with Grade 1 CRS had a median onset of 4 days, whereas Grade 2 CRS had a median onset of 3 days. For Grade 3 CRS, the median timeline was 6 days, and Grade 4 CRS had a median onset of 5 days (Fig 5, Table 4). The most frequent clinical interventions were, for Grade 0 CRS, daily assessment of CRS. For Grade 1 CRS, administration of tocilizumab, for Grade 2 CRS, dexamethasone 10 mg IV Q6H, for Grade 3 CRS, administration of dexamethasone 10 mg IV Q6H, for Grade 4 CRS, the use of methylprednisone pulse therapy (S1 Fig, S7–S13 Tables).

**Table 4:** Frequencies and timelines of CRS Grades and ICANS Grades.

	Frequency	Percentage	Min	Q1	Median	Q3	Max
CRS occurrence	239		0	2	4	7	30
CRS Grades							
0	71	29.7	0	2	5	8	30
1	142	59.4	0	2	4	7	22
1-2	≤10	≤4.2	0	1	7	7	29
2	76	31.8	0	2	3	7	20
2-3	≤10	≤4.2	1	1	2	5	8
3	≤10	≤4.2	0	5	6	9	11
4	≤10	≤4.2	5	5	5	5.5	6
Unknown	149	62.3	0	1	4	6	24
ICANS occurrence	132		0	3	5	8	27
ICANS Grade							
0	55	41.7	0	2	5	8	27
1	33	25.0	0	3	6	8	22
1-2	≤10	≤7.6	6	6	6	6	6
2	37	28.0	0	4	6	8	23
2-3	≤10	≤7.6	5	5	5	5	5
3	12	9.1	0	5	6	8	11
3-4	≤10	≤7.6	6	6.5	7	7.5	8
4	≤10	≤7.6	6	6.5	7	7	7
Unknown	74	56.1	0	3	5	8	27

**Fig 6:**
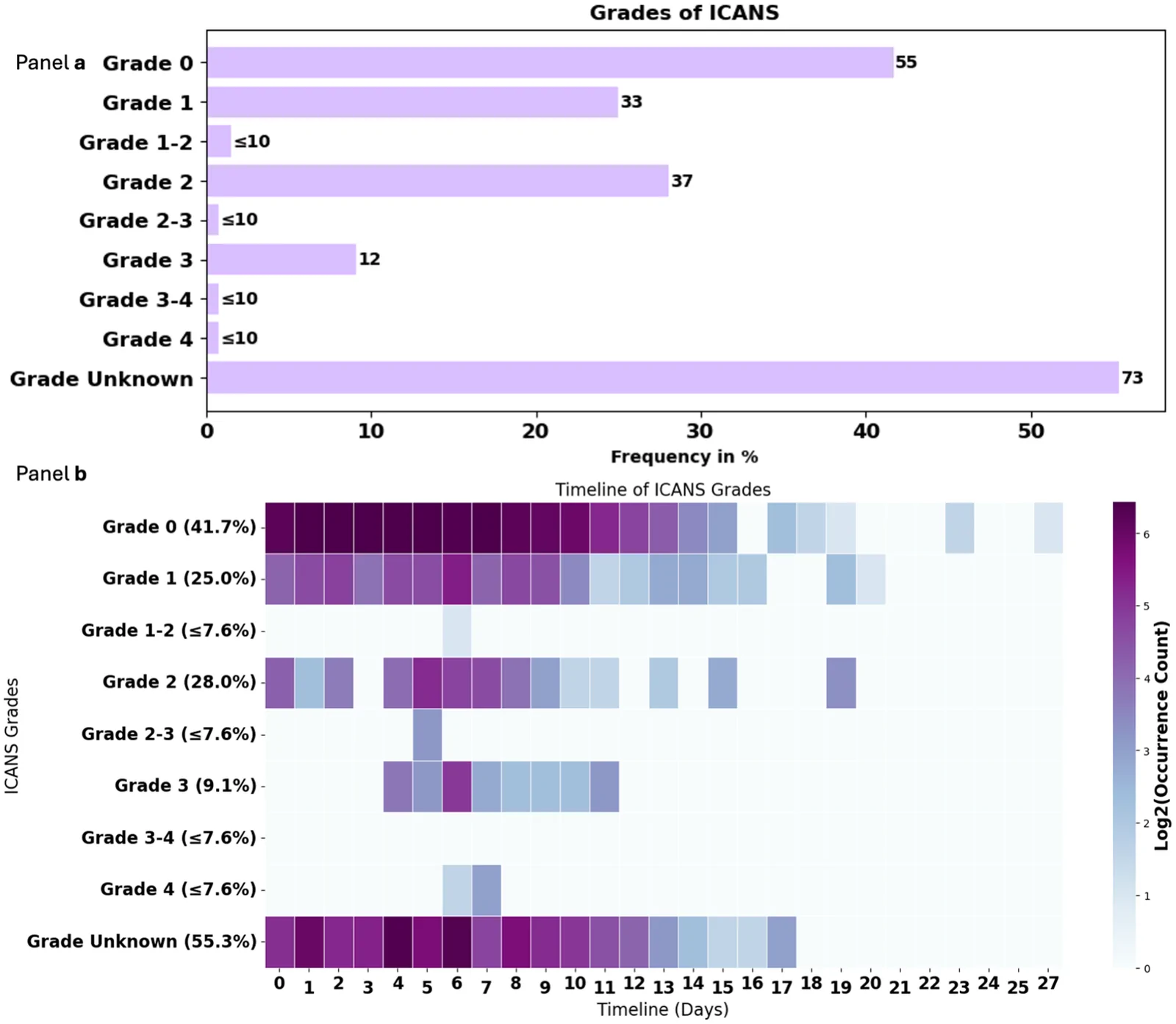
ICANS grades: frequencies (bar chart) and timelines (heat map) for our patients experiencing ICANS (n = 132 patients), identified using a grade-focused prompt during ICANS event detection. This figure presents the frequencies and timelines of occurrence of Immune Effector Cell-Associated Neurotoxicity Syndrome (ICANS) grades in the 132 patients with ICANS from our cohort of 293 patients. Panel a) Displays a horizontal bar chart illustrating the frequencies of ICANS grades as a percentage of the total patients identified with ICANS. The grades are ranked from least to most severe. Panel b) Shows a heatmap depicting the timeline of ICANS grades over the 30-day follow-up time window. The x-axis represents the timeline in days, while the y-axis lists the ICANS grades and their frequencies. The color intensity corresponds to the log2-transformed occurrence count in clinical notes, with darker purple indicating higher frequencies. Abbreviations used in the figure: ICANS: Immune Effector Cell-Associated Neurotoxicity Syndrome.

The prompt designed to identify ICANS grades reported that ICANS occurred in 132 (45.1%) patients. Because each prompt was run independently and outputs were not reconciled across prompts, the patient-level ICANS count from the ICANS-specific prompt differed from the ICANS count extracted by the prespecified AE prompt. Among those, Grade 0 which correspond to absence of ICANS was the most commonly extracted information, observed in 41.7% of the 132 cases, followed by Grade 2 (28.0%) and Grade 1 (25.0%). Higher grades of ICANS, including Grade 3 (9.1%), Grade 3–4 (≤7.6%), and Grade 4 (≤7.6%), were less frequent. Grade Unknown was reported in 56.1% of cases, potentially reflecting again incomplete documentation. Because more than one ICANS grade could be recorded for an individual patient across different notes or time points, the grade-specific percentages are not mutually exclusive and should not be added to total 100%; instead, they indicate the overall frequency with which each grade was identified. (Fig 6, Table 4). ICANS median timeline was 5–7 days post-CAR-T therapy. (Fig 6, Table 4). The most frequent clinical intervention was seizure prophylaxis with Keppra for Grade 0 ICANS, no intervention for Grade 1, dexamethasone 10mg IV q6h for Grades 2 and 3, and Solumedrol 1 gram IV daily for 3 days for Grade 4 (S2 Fig, S14–S21 Tables).

## Discussion

Adverse events (AEs) after Chimeric Antigen Receptor T-cell (CAR-T) therapy are common and often severe, making timely detection and intervention essential for patient safety. However, much of the clinical detail needed to understand AE onset, severity, and management is buried in unstructured notes that are difficult to analyze at scale. Leveraging large language models (LLMs) to extract these patterns offers a promising way to better characterize AE trajectories and ultimately improve monitoring and outcomes for CAR-T recipients.

In our study, the LLM demonstrated high point estimate performance for CRS- and ICANS-specific prompts, particularly for presence and grade. For broader prespecified AE prompts, point estimates were moderate and showed evaluator-dependent variability. Although the validation sample was not powered for an accurate estimate of LLM performance, this likely reflects the ambiguity and variability of AE documentation in unstructured clinical notes and the inherent challenges of using an LLM to identify multiple categories of adverse events. In contrast, agreement was higher for CRS and ICANS presence and grading, likely due to their more standardized clinical definitions and documentation. When using the LLM in our data, trends in AE occurrence highlighted CRS as the most frequently reported event, followed by neutropenia in prevalence, with each displaying characteristic timing patterns post-treatment. Prompts designed to extract grades provided insight into the distribution of severity, with lower grades predominating for CRS and ICANS. These results are supported by the strong accuracy and IRR; however, limitations in data completeness occasionally hindered precise grade assignment. These findings underscore the potential of LLMs to reliably capture key information on CRS and ICANS following CAR-T, while also generating hypotheses for areas where heterogeneity across other AEs and incomplete documentation may affect performance, warranting cautious interpretation of the results for this task and calling for further research.

Recent work has demonstrated the effective application of LLMs to extract clinical information from unstructured data [31–35]. Moreover, emerging studies have shown good performance in the application of these techniques for adverse event detection. While emphasizing challenges such as domain-specific variability in model performance, large language models and LLM-based pipelines have shown strong performance for adverse drug event extraction and structured AE grades, with F1-scores between 0.62 and 0.99, outperforming traditional machine learning models and ICD-based systems [36–41]. Point estimates for performance derived from our study validation sample aligned with these previous findings, highlighting that a commercial LLM could demonstrate moderate to high performance metrics for extraction of CAR-T related AEs, similar to those reported in these prior studies that include oncology and also other domains [36–41]. These results encourage physicians and researchers to conduct further research using these tools in CAR-T monitoring, clinical trials, and real-world evidence research, while emphasizing the need for careful supervision and improving and standardizing performance.

Across pivotal CAR-T cell therapy trials and large real-world cohorts, CRS and ICANS were frequently reported which aligned with the extraction we performed on UCSF data using LLMs. Moreover, in our study, the model captured low-frequency, non-prespecified events such as rash and constipation with good accuracy and IRR, which may serve as early warning signs of broader toxicity syndromes or signal unexpected AEs. In the pivotal ZUMA-1 trial of axicabtagene ciloleucel, the most common AEs of any grade were fever/pyrexia (85% of the patients), neutropenia (84%), and anemia (66%). CRS occurred in 93% of patients, with grade ≥ 3 events in about 13%, while neurologic AEs in 64% with 28% grade ≥ 3 [1]. The JULIET trial of tisagenlecleucel reported rates of CRS (58%), anemia (48%), neutropenia (34%) and any neurologic events (12%) [3], and the TRANSCEND study of lisocabtagene maraleucel demonstrated lower frequencies of neutropenia (60%), anemia (37%), CRS (42%) and ICANS (30%) [42]. Our study included several CAR-T therapies and was conducted in a real‑world setting, which might explain differences to clinical trials of single drugs, although our results preserved relative proportions of the common AEs and fit what is clinically expected. Meta-analytic evidence also supports the prevalence of these AEs, indicating pooled CRS and ICANS incidences of 70–90% and 25–30%, respectively, with pooled high-grade CRS under 15%, under 30% for all grades ICANS about 10% for high-grade ICANS [8,43]. Furthermore, large multicenter registries have reported similar rates of severe CRS and ICANS, with most events occurring within the first two weeks post-infusion and resolving with tocilizumab or corticosteroids, which validate the timeline and interventions that we were able to extract [44–46]. This data along with our performance analysis on our validation sample shows that LLMs exhibit high point estimates for performance in identifying CRS and ICANS occurrence and their respective grades. Our study also showed the high prevalence of CRS, ICANS, neutropenia, anemia, and infections in the real-world CAR-T setting, consistent with previous clinical trials and registry-based analyses. Furthermore, we showed that these AEs typically occur early after infusion, often within the first two weeks, highlighting the critical need for close monitoring and early intervention [13,46]. Severity, and current and previous care are essential considerations in the management of ongoing or evolving toxicities, and can significantly influence the trajectory and recovery from CRS, ICANS, or hematologic AE [9,10,13,44,46]. Our LLM based system demonstrated high accuracy in extracting the occurrence and grades of CRS and ICANS from clinical notes, offering substantial potential to enhance real-time patient monitoring, clinical decision support, and pharmacovigilance monitoring. These findings support further supervised evaluation and validation of LLM-driven extraction tools in clinical and research settings to complement existing manual or structured data sources within oncology practice and research.

This work has several important strengths, while also having limitations that merit consideration. A key limitation is the small manual review sample relative to the number of extraction tasks assessed. The sample is not powered to provide narrow estimates of the model performances and estimates could vary with small changes in counts. In addition, multiple elements within one note were not independent and one note could include content copied forward from previous records or notes without explicit formatting, which can further complicate interpretation and manual assessment of the extracted adverse events. For these reasons, we interpreted performance estimates as preliminary and placed greater emphasis on the qualitative pattern of results (notably, higher point estimates for CRS/ICANS presence and grade). Therefore, all subgroup-level findings, particularly for prespecified and non-prespecified AEs, should be regarded as hypothesis generating. Another limitation is the variability in inter‑rater reliability (IRR), and the discrepancy patterns help localize where performance and agreement were most fragile. First, temporality was difficult to adjudicate when notes contained multiple absolute dates, relative day counts, or carry‑forward text. Evaluators assessed plain‑text exports without the usual EHR sectioning or time stamps, which further impeded determining AE occurrence relative to infusion. Second, prespecified AEs that can be inferred from both laboratory values and narrative (anemia, thrombocytopenia, neutropenia, leukopenia) could have been documented inconsistently across text and labs and were sometimes historical or resolving; clinicians and evaluators may also have applied different thresholds. In our sensitivity analysis excluding these lab‑based cytopenias, IRR improved for AE nature, supporting this interpretation, though agreement for dates and interventions remained lower. Third, AE and intervention mentions were highly heterogeneous; although causality and imputation were not requested, implicit clinical reasoning about whether an event was CAR‑T–related may have influenced extraction and evaluators’ review which also may reflect differences in the evaluators’ training backgrounds. Fourth, de‑identification placeholders (*****), abbreviations, and copy‑forward practices sometimes obscured extraction, evaluation, or whether text reflected a plan, prophylaxis, or an administered therapy, further increasing variability in IRR. Taken together, these factors suggest that weaker kappas for prespecified and non-prespecified AEs reflect documentation ambiguity and annotation-framework complexity at least as much as model limitations. Event extraction relied heavily on documentation quality, and the high proportion of “grade unknown” for CRS and ICANS highlights incomplete or inconsistent charting. This highlights variability in the way physicians document their notes and suggests that grades are not always explicitly stated but are often expected to be inferred by clinicians reading one another’s notes. This calls for further research into LLM interpretation of clinical information to determine occurrence of adverse AEs. Class imbalance (e.g., fewer positive cases for some non-prespecified events) was another limitation, reflecting the lower likelihood of occurrence for certain AEs. Limiting the analysis to progress notes optimized our use of resources and likely reduced heterogeneity, but it may have excluded clinically meaningful AE signals captured elsewhere (e.g., nursing notes for vitals and supportive care, consults for subspecialty assessments, ED notes for early symptoms, and discharge summaries for consolidated course/grade rationales). As a result, some AE dates or interventions may have been missed when they were documented exclusively in non-progress note types. An important next step would be to extend extraction to targeted additional note types. Furthermore, because only length-eligible progress notes were analyzed, the retained note set represented a selected subset of all available post-infusion documentation, which may have introduced selection bias if adverse events, interventions, or severity details were preferentially documented in excluded note types or in notes outside the token-length range. Additionally, the model’s context-window limitations required exclusion of longer notes, limiting our ability to assess performance on lengthy, longitudinal charts spanning multiple encounters and potentially obscuring cross-document dependencies. This limitation may be mitigated by newer models with substantially larger context windows than the gpt-4–0314 model used in this study, enabling more complete evaluation of longer clinical narratives and cross-note relationships. Non-prespecified AEs and clinical interventions were not embedded by any methods to reduce computational steps and minimize the time required for reviewers. Clustering these would enhance interpretation and highlight the need for further research on how to integrate this step. The study employed zero-shot prompting without additional training, context, or guidelines for the model, which may have impacted overall performance but provides a foundation for future methodological refinements, performance improvements and represents an important step in oncology toward establishing the potential of LLMs to identify and extract CAR-T-related AEs from clinical notes. Moreover, we envision evaluating this method in other healthcare systems and with additional model versions to better assess generalizability, robustness, and potential future applications.

## Conclusion

We applied and evaluated an LLM to analyze clinical notes and extract AEs, including CRS and ICANS presence, grades, dates, and related interventions, from unstructured progress notes. Point estimates for performance were high for CRS and ICANS presence and grade, with strong to near-perfect inter-rater reliability, moderate-to-high point estimates for CRS and ICANS dates and interventions, and more variable performance for broader AE nature, date, and intervention categories.

These results support further prospective evaluation of supervised LLM-based extraction of CRS and ICANS grading in EHR-based research or pilot settings, accompanied by prospective monitoring and expanded validation. Broader AE extraction should remain exploratory until supported by larger, more diverse manual evaluations.

Documentation variability and class imbalance for broader AEs remain challenges, highlighting the need for further research into LLM interpretation of clinical results. Additionally, cautious use of LLMs in a zero-prompt manner for broad AE extraction is warranted due to the variable performance observed in this study. Nevertheless, this study provides a foundation for further research, including piloting the introduction of LLMs into real-world evidence studies and safety monitoring in clinical trials and oncology practice, while ensuring careful supervision and ensuring that performance metrics are carefully monitored and interpreted.

## Supporting information

S1 Annotation Guide Annotation Guide for annotators. (PDF)

S1 Fig Clinical interventions by CRS grade. (TIFF)

S2 Fig Clinical interventions by ICANS grade. (TIFF)

S1 Table Prompt texts for AE (prespecified), non-prespecified AE, CRS, and ICANS extraction. (DOCX)

S2 Table Note types and patient counts (cohort overview). (DOCX)

S3 Table LLM performance by prompt and reviewer (accuracy, precision, recall, F1, kappa). (DOCX)

S4 Table Top 50 interventions for prespecified AEs. (DOCX)

S5 Table Categories and examples of non-prespecified AEs. (DOCX)

S6 Table Top 50 interventions for non-prespecified AEs. (DOCX)

S7 Table Top 30 interventions for CRS Grade 0. (DOCX)

S8 Table Top 30 interventions for CRS Grade 1. (DOCX)

S9 Table Top interventions for CRS Grades 1–2. (DOCX)

S10 Table Top 30 interventions for CRS Grade 2. (DOCX)

S11 Table All interventions for CRS Grades 2–3. (DOCX)

S12 Table All interventions for CRS Grade 3. (DOCX)

S13 Table All interventions for CRS Grade 4. (DOCX)

S14 Table Top 30 interventions for ICANS Grade 0. (DOCX)

S15 Table Top 30 interventions for ICANS Grade 1. (DOCX)

S16 Table All interventions for ICANS Grades 1–2. (DOCX)

S17 Table Top 30 interventions for ICANS Grade 2. (DOCX)

S18 Table All interventions for ICANS Grades 2–3. (DOCX)

S19 Table Top 30 interventions for ICANS Grade 3. (DOCX)

S20 Table All interventions for ICANS Grades 3–4. (DOCX)

S21 Table All interventions for ICANS Grade 4. (DOCX)
