# Supplementary material for: Extracting adverse event nature, severity, timelines and resulting interventions from clinical notes of patients receiving CAR-T cell therapy using large language models
Source: PLOS Digit Health. 2026 Aug 27;5(8):e0001426. doi: 10.1371/journal.pdig.0001426 (PMC13521353; doi:10.1371/journal.pdig.0001426)
Supplement: Supplementary file 1 [file pdig.0001426.s001.pdf]

CAR-T adverse events - Annotation Guidelines

<u>**Data overview**</u>

In the Box Folder, you will find 50 folders each named ***'Xnote_keyX'*_note_result**. Each folder corresponds to one note and will include four CSV files

1. ***'Xnote_keyX'***.csv
2. Prompt1_***'Xnote_keyX'***.csv
3. Prompt2_***'Xnote_keyX'***.csv
4. Prompt3_***'Xnote_keyX'***.csv
5. Prompt4_***'Xnote_keyX'***.csv

The **'X*note_keyX'*.csv** file is where you will find the note. The **Prompt1_*'Xnote_keyX'*.csv**, **Prompt2_*'Xnote_keyX'*.csv**, **Prompt3_*'Xnote_keyX'*.csv** and **Prompt4_*'Xnote_keyX'*.csv** are the files that contains results and need to be annotated.

For each file you will have columns with results from the prompt and columns that needs to be annotated simply by a **Yes (Y) or No (N) answer**. Following is a description of each file and their columns

Prompt 1

Prompt 1 is:

"""Task: Extract adverse events due to CAR-T administration, their dates (format mm-dd-yyyy), and the clinical interventions performed from the clinical note provided. Format the output in CSV format with the headers "Adverse event", "Date of Adverse Event", and "Clinical intervention". Ensure that the "Adverse event" is one of the following values: "Cytokine Release Syndrome (CRS)", "Immune Effector Cell-Associated Neurotoxicity Syndrome (ICANS)", "Hypogammaglobulinemia", "Neutropenic Fever", "Macrophage Activation Syndrome (MAS)", "Hemophagocytic Lymphohistiocytosis (HLH)", "Tumor Lysis Syndrome", "Movement disorder", "Cognitive impairment", "Personality changes", "B-cell aplasia", "Thrombocytopenia", "Anemia", "Neutropenia", "Leukopenia", "Hypogammaglobulinemia", "CD4 lymphopenia", "Cranial Nerve Palsy", "Bacterial infection", "Viral Infection", "Zoster Infection", "Fungal infection", "Aspergillus infection", "Pneumocystis Jirovecii Pneumonia infection", "Secondary myeloid malignancies", and "Graft-versus-host disease (GVHD)". If no adverse event is identified, fill "Adverse event" with "No" and "NA" for "Date of Adverse Event" and "Clinical intervention". If an adverse event is identified but no date or clinical intervention is provided, note "NA" for "Date of Adverse Event" or "Clinical intervention". Information mentioned in the transfusion threshold is a general guideline for clinicians and might not reflect the occurrence of an adverse event or a clinical intervention."""

The **Prompt1_*'Xnote_keyX'*.csv** is where you will record your annotations, and contains the following columns. The columns highlighted in green are ones for you to fill out.

Column	Description
**note_key**	A unique identifier for each note
**Adverse event prespecified**	An adverse event (AE) identified in the note and among the AE prespecified in the prompt
**Date of Adverse Event**	Date

**Clinical intervention to prespecified AE**	A clinical intervention identified as corresponding to the AE identified
**AE is correct and among prespecified**	Needs annotation **Y** or **N**
**If previous column is Y, Is the date correct?**	Needs annotation **Y** or **N**
**AE is correct but not among prespecified**	Needs annotation **Y** or **N**
**AE is not an AE but a comorbidity**	Needs annotation **Y** or **N**
**AE is in the note but neither an AE nor a comorbidity (**e.g an adverse event but not related to CAR-T, a clinical observations not being a comorbidity)	Needs annotation **Y** or **N**
**AE is hallucination** (meaning that there is no mention of the identified adverse event in the note, regardless of specified or unspecified)	Needs annotation **Y** or **N**
**Clinical intervention is correct and related to the AE regardless of type of AE**	Needs annotation **Y** or **N**
**Clinical intervention is hallucination** (meaning that there is no mention of the identified clinical intervention in the note, regardless the appropriateness or clinical rationality of the intervention)	Needs annotation **Y** or **N**
**A prespecified AE is in the note and has not been recorded**	Needs only one annotation for the whole note: answer **Y** or **N** **for all rows**
**A clinical intervention for a prespecified adverse event (AE) is noted but has not been recorded, regardless of whether the AE has been identified.**	Needs only one annotation for the whole note: answer **Y** or **N** **for all rows**

Annotation overview

To be considered 'correct', an adverse event must be related to the CAR-T **and** mentioned in the note. Prespecified adverse event are the following:

- "Cytokine Release Syndrome (CRS)"
- "Immune Effector Cell-Associated Neurotoxicity Syndrome (ICANS)"
- "Hypogammaglobulinemia"
- "Neutropenic Fever"
- "Macrophage Activation Syndrome (MAS)"
- "Hemophagocytic Lymphohistiocytosis (HLH)"
- "Tumor Lysis Syndrome"
- "Movement disorder"
- "Cognitive impairment"
- "Personality changes"
- "B-cell aplasia"
- "thrombocytopenia"
- "anemia"
- "neutropenia"
- "leukopenia"
- "hypogamma-globulinemia"
- "CD4 lymphopenia"
- "Cranial Nerve Palsy"
- "Bacterial infection"
- "Viral Infection"
- "Zoster Infection"
- "Fungal infection"
- "Aspergillus infection"
- "Pneumocystis Jirovecii Pneumonia infection"
- "Secondary myeloid malignancies"
- "Graft-versus-host disease (GVHD)"

Prompt 2

Prompt 2 is:

"""Task: Extract adverse events due to CAR-T administration, their dates (format mm-dd-yyyy), and clinical interventions from the clinical note provided. Format the output in CSV format with the headers "Adverse event", "Date of Adverse Event", and "Clinical intervention". Exclude adverse events with the following categories: "Cytokine Release Syndrome (CRS)", "Immune Effector Cell-Associated Neurotoxicity Syndrome (ICANS)", "Hypogammaglobulinemia", "Neutropenic Fever", "Macrophage Activation Syndrome (MAS)", "Hemophagocytic Lymphohistiocytosis (HLH)", "Tumor Lysis Syndrome", "Movement disorder", "Cognitive impairment", "Personality changes", "B-cell aplasia", "Thrombocytopenia", "Anemia", "Neutropenia", "Leukopenia", "Hypogammaglobulinemia", "CD4 lymphopenia", "Cranial Nerve Palsy", "Bacterial infection", "Viral Infection", "Zoster Infection", "Fungal infection", "Aspergillus infection", "Pneumocystis Jirovecii Pneumonia infection", "Secondary myeloid malignancies", and "Graft-versus-host disease (GVHD)". If no adverse event is identified, fill "Adverse Event" with "No" and "NA" for "Date of Adverse Event" and "Clinical intervention". If an adverse event is identified but no date or clinical intervention is provided, note "NA" for "Date of Adverse Event" or "Clinical intervention". Information mentioned in the transfusion threshold is a general guideline for clinicians and might not reflect the occurrence of an adverse event or a clinical intervention."""

The **Prompt2_*'Xnote_keyX'*.csv** is where you will record your annotations, and contains the following columns. The columns highlighted in green are ones for you to fill out.

**Column**	**Description**
**note_key**	A unique identifier for each note
**Adverse event not prespecified**	An adverse event (AE) identified in the note and not among the AE prespecified in the prompt
**Date of Adverse Event**	Date

**Clinical intervention to non prespecified AE**	A clinical intervention identified as corresponding to the AE identified
**AE is correct and among non prespecified**	Needs annotation **Y** or **N**
**If previous column is Y, Is the date correct?**	Needs annotation **Y** or **N**
**AE is correct but among prespecified**	Needs annotation **Y** or **N**
**AE is not an AE but a comorbidity**	Needs annotation **Y** or **N**
**AE is in the note but neither an AE nor a comorbidity (**e.g an adverse event but not related to CAR-T, a clinical observations not being a comorbidity)	Needs annotation **Y** or **N**
**AE is hallucination** (meaning that there is no mention of the identified adverse event in the note, regardless of specified or unspecified)	Needs annotation **Y** or **N**
**Clinical intervention is correct and related to the AE regardless of type of AE**	Needs annotation **Y** or **N**
**Clinical intervention is hallucination** (meaning that there is no mention of the identified clinical intervention in the note, regardless the appropriateness or clinical rationality of the intervention)	Needs annotation **Y** or **N**
**A non prespecified AE is in the note and has not been recorded**	Needs only one annotation for the whole note: answer **Y** or **N for all rows**
**A clinical intervention for a non prespecified AE is noted but has not been recorded, regardless of whether the AE has been identified**	Needs only one annotation for the whole note: answer **Y** or **N for all rows**

<u>Annotation overview</u>

To be considered 'correct', an adverse event must be related to the CAR-T **and** mentioned in the note

Prespecified adverse event are listed in prompt 1

Prompt 3

Prompt 3 is:

"""Task: Extract the occurrence of Cytokine Release Syndrome (CRS), the grade of CRS, and clinical interventions from the provided clinical note. Format the output in CSV format with the headers "CRS", "CRS grade", "Date of occurrence of CRS", and "Clinical intervention". If CRS is identified, fill "CRS" with "Yes", "CRS grade" with the corresponding grade (format Grade X, Grade could be between 0 to 4), "Date of occurrence of CRS" with the date (format mm-dd-yyyy) of CRS, and "Clinical intervention" with the clinical intervention performed to treat the CRS. If there is no CRS, fill "CRS" with "No" and "NA" for the "CRS grade", "Date of occurrence of CRS", and "Clinical intervention". If CRS is identified but no CRS grade, no date, or no clinical intervention is identified, note "Yes" for "CRS" and "NA" for the "CRS grade", "Date of occurrence of CRS", or "Clinical intervention". Information mentioned in the transfusion threshold is a general guideline for clinicians and might not reflect the occurrence of CRS or a clinical intervention."""

The **Prompt3_*'Xnote_keyX'*.csv** is where you will record your annotations, and contains the following columns. The columns highlighted in green are ones for you to fill out.

**Column**	**Description**
**note_key**	A unique identifier for each note
**CRS**	Identify the presence of CRS
**CRS grade**	Corresponding grade of the CRS identified
**Date of occurrence of CRS**	Corresponding date of the CRS identified
**Clinical intervention for CRS**	Corresponding Intervention for the CRS identified

**CRS is identified and the grade is correctly reported from the note** (If no grade is reported in the note and no grade is reported in the output, this is considered correct, so (Y). Grade can be between 0 to 4. If a Grade 0 is reported because mentioned in the note this is considered correct, so (Y))	Needs annotation **Y** or **N**
**CRS is identified but the grade is incorrectly reported from the note or guessed**	Needs annotation **Y** or **N**
**CRS date correspond to this grade and is correct**	Needs annotation **Y** or **N**
**There is no mention of CRS in this note - hallucination**	Needs only one annotation for the whole note: answer **Y** or **N for all rows**
**clinical intervention is correct and related to the CRS and its grade**	Needs annotation **Y** or **N**
**clinical intervention is noted but related to another grade of CRS**	Needs annotation **Y** or **N**
**clinical intervention is hallucination or guessed** (meaning that there is no mention of the identified clinical intervention in the note, regardless the appropriateness or clinical rationality of the intervention)	Needs annotation **Y** or **N**
**A CRS is in the note and has not been recorded**	Needs only one annotation for the whole note: answer **Y** or **N for all rows**
**A CRS grade is in the note and has not been recorded**	Needs only one annotation for the whole note: answer **Y** or **N for all rows**

**A CRS date is in the note and has not been recorded**	Needs only one annotation for the whole note: answer **Y** or **N for all rows**
**A clinical intervention for a CRS is noted but has not been recorded, regardless of whether the CRS has been identified**	Needs only one annotation for the whole note: answer **Y** or **N for all rows**

Prompt 4

Prompt 4 is:

"""Task: Extract the occurrence of Immune Effector Cell-Associated Neurotoxicity Syndrome (ICANS), the grade of ICANS, and clinical interventions from the provided clinical note. Format the output in CSV format with the headers "ICANS", "ICANS grade", "Date of occurrence of ICANS", and "Clinical intervention". If ICANS is identified, fill "ICANS" with "Yes", "ICANS grade" with the corresponding grade (format Grade X, Grade could be between 0 to 4*), "Date of occurrence of ICANS" with the date (format mm-dd-yyyy) of ICANS, and "Clinical intervention" with the clinical intervention performed to treat the ICANS. If there is no ICANS, fill "ICANS" with "No" and "NA" for the "ICANS grade", "Date of occurrence of ICANS", and "Clinical intervention". If ICANS is identified but no ICANS grade, no date, or no clinical intervention is identified, note "Yes" for "ICANS" and "NA" for the "ICANS grade", "Date of occurrence of ICANS", and "Clinical intervention". Information mentioned in the transfusion threshold is a general guideline for clinicians and might not reflect the occurrence of ICANS or a clinical intervention."""*

The **Prompt4_'*Xnote_keyX*'.csv** is where you will record your annotations, and contains the following columns. The columns highlighted in green are ones for you to fill out.

Column	Description
**note_key**	A unique identifier for each note
**ICANS**	Identify the presence of ICANS
**ICANS grade**	Corresponding grade of the ICANS identified
**Date of occurrence of ICANS**	Corresponding date of the ICANS identified

**Clinical intervention for ICANS**	Corresponding Intervention for the ICANS identified
**ICANS is identified and the grade is correctly reported from the note** (If no grade is reported in the note and no grade is reported in the output, this is considered correct, so (Y). Grade can be between 0 to 4. If a Grade 0 is reported because mentioned in the note this is considered correct, so (Y))	Needs annotation **Y** or **N**
**ICANS is identified but the grade is incorrectly reported from the note or guessed.**	Needs annotation **Y** or **N**
**ICANS date correspond to this grade and is correct**	Needs annotation **Y** or **N**
**There is no mention of ICANS in this note - hallucination**	Needs only one annotation for the whole note: answer **Y** or **N for all rows**
**clinical intervention is correct and related to the ICANS and its grade**	Needs annotation **Y** or **N**
**clinical intervention is noted but related to another grade of ICANS**	Needs annotation **Y** or **N**
**clinical intervention is hallucination or guessed** (meaning that there is no mention of the identified clinical intervention in the note, regardless the appropriateness or clinical rationality of the intervention)	Needs annotation **Y** or **N**
**A ICANS is in the note and has not been recorded**	Needs only one annotation for the whole note: answer **Y** or **N for all rows**

**A ICANS grade is in the note and has not been recorded**	Needs only one annotation for the whole note: answer **Y** or **N for all rows**
**A ICANS date is in the note and has not been recorded"**	Needs only one annotation for the whole note: answer **Y** or **N for all rows**
**A clinical intervention for a ICANS is noted but has not been recorded, regardless of whether the ICANS has been identified**	Needs only one annotation for the whole note: answer **Y** or **N for all rows**

Thank you for helping with annotations!
