# Supplementary material for: Extracting adverse event nature, severity, timelines and resulting interventions from clinical notes of patients receiving CAR-T cell therapy using large language models
Source: PLOS Digit Health. 2026 Aug 27;5(8):e0001426. doi: 10.1371/journal.pdig.0001426 (PMC13521353; doi:10.1371/journal.pdig.0001426)
Supplement: Supplementary file 2 [file pdig.0001426.s004.docx]

S1 Table Prompts used for extracting: (i) prespecified adverse events, dates of occurrence, and clinical interventions; (ii) non-prespecified adverse events, their dates of occurrence, and clinical interventions; (iii) CRS occurrences, their grades, dates, and clinical interventions; and (iv) ICANS occurrences, their grades, dates, and clinical interventions.

prompt1 = """Task: Extract adverse events due to CAR-T administration, their dates (format mm-dd-yyyy), and the clinical interventions performed from the clinical note provided. Format the output in CSV format with the headers “Adverse event”, “Date of Adverse Event”, and “Clinical intervention”. Ensure that the “Adverse event” is one of the following values: “Cytokine Release Syndrome (CRS)”, “Immune Effector Cell-Associated Neurotoxicity Syndrome (ICANS)”, “Hypogammaglobulinemia”, “Neutropenic Fever”, “Macrophage Activation Syndrome (MAS)”, “Hemophagocytic Lymphohistiocytosis (HLH)”, “Tumor Lysis Syndrome”, “Movement disorder”, “Cognitive impairment”, “Personality changes”, “B-cell aplasia”, “Thrombocytopenia”, “Anemia”, “Neutropenia”, “Leukopenia”, “Hypogammaglobulinemia”, “CD4 lymphopenia”, “Cranial Nerve Palsy”, “Bacterial infection”, “Viral Infection”, “Zoster Infection”, “Fungal infection”, “Aspergillus infection”, “Pneumocystis Jirovecii Pneumonia infection”, “Secondary myeloid malignancies”, and “Graft-versus-host disease (GVHD)”. If no adverse event is identified, fill “Adverse event” with “No” and “NA” for “Date of Adverse Event” and “Clinical intervention”. If an adverse event is identified but no date or clinical intervention is provided, note “NA” for “Date of Adverse Event” or “Clinical intervention”. Information mentioned in the transfusion threshold is a general guideline for clinicians and might not reflect the occurrence of an adverse event or a clinical intervention."""

prompt2 = """Task: Extract adverse events due to CAR-T administration, their dates (format mm-dd-yyyy), and clinical interventions from the clinical note provided. Format the output in CSV format with the headers “Adverse event”, “Date of Adverse Event”, and “Clinical intervention”. Exclude adverse events with the following categories: “Cytokine Release Syndrome (CRS)”, “Immune Effector Cell-Associated Neurotoxicity Syndrome (ICANS)”, “Hypogammaglobulinemia”, “Neutropenic Fever”, “Macrophage Activation Syndrome (MAS)”, “Hemophagocytic Lymphohistiocytosis (HLH)”, “Tumor Lysis Syndrome”, “Movement disorder”, “Cognitive impairment”, “Personality changes”, “B-cell aplasia”, “Thrombocytopenia”, “Anemia”, “Neutropenia”, “Leukopenia”, “Hypogammaglobulinemia”, “CD4 lymphopenia”, “Cranial Nerve Palsy”, “Bacterial infection”, “Viral Infection”, “Zoster Infection”, “Fungal infection”, “Aspergillus infection”, “Pneumocystis Jirovecii Pneumonia infection”, “Secondary myeloid malignancies”, and “Graft-versus-host disease (GVHD)”. If no adverse event is identified, fill “Adverse Event” with “No” and “NA” for “Date of Adverse Event” and “Clinical intervention”. If an adverse event is identified but no date or clinical intervention is provided, note “NA” for “Date of Adverse Event” or “Clinical intervention”. Information mentioned in the transfusion threshold is a general guideline for clinicians and might not reflect the occurrence of an adverse event or a clinical intervention."""

prompt3 = """Task: Extract the occurrence of Cytokine Release Syndrome (CRS), the grade of CRS, and clinical interventions from the provided clinical note. Format the output in CSV format with the headers “CRS”, “CRS grade”, “Date of occurrence of CRS”, and “Clinical intervention”. If CRS is identified, fill “CRS” with “Yes”, “CRS grade” with the corresponding grade (format Grade X, Grade could be between 0 to 4), “Date of occurrence of CRS” with the date (format mm-dd-yyyy) of CRS, and “Clinical intervention” with the clinical intervention performed to treat the CRS. If there is no CRS, fill “CRS” with “No” and “NA” for the “CRS grade”, “Date of occurrence of CRS”, and “Clinical intervention”. If CRS is identified but no CRS grade, no date, or no clinical intervention is identified, note “Yes” for “CRS” and “NA” for the “CRS grade”, “Date of occurrence of CRS”, or “Clinical intervention”. Information mentioned in the transfusion threshold is a general guideline for clinicians and might not reflect the occurrence of CRS or a clinical intervention."""

prompt4 = """Task: Extract the occurrence of Immune Effector Cell-Associated Neurotoxicity Syndrome (ICANS), the grade of ICANS, and clinical interventions from the provided clinical note. Format the output in CSV format with the headers “ICANS”, “ICANS grade”, “Date of occurrence of ICANS”, and “Clinical intervention”. If ICANS is identified, fill “ICANS” with “Yes”, “ICANS grade” with the corresponding grade (format Grade X, Grade could be between 0 to 4), “Date of occurrence of ICANS” with the date (format mm-dd-yyyy) of ICANS, and “Clinical intervention” with the clinical intervention performed to treat the ICANS. If there is no ICANS, fill “ICANS” with “No” and “NA” for the “ICANS grade”, “Date of occurrence of ICANS”, and “Clinical intervention”. If ICANS is identified but no ICANS grade, no date, or no clinical intervention is identified, note “Yes” for “ICANS” and “NA” for the “ICANS grade”, “Date of occurrence of ICANS”, and “Clinical intervention”. Information mentioned in the transfusion threshold is a general guideline for clinicians and might not reflect the occurrence of ICANS or a clinical intervention."""
