# Supplementary material for: Extracting adverse event nature, severity, timelines and resulting interventions from clinical notes of patients receiving CAR-T cell therapy using large language models
Source: PLOS Digit Health. 2026 Aug 27;5(8):e0001426. doi: 10.1371/journal.pdig.0001426 (PMC13521353; doi:10.1371/journal.pdig.0001426)
Supplement: Supplementary file 3 [file pdig.0001426.s005.docx]

S2 Table Frequencies of note types and corresponding patient counts in the full cohort.

| **note_type** | **note count** | **note percent** | **patients count** | **patients percent** |
| --- | --- | --- | --- | --- |
|  | **n=18808** |  | **n=293** |  |
| Progress Notes | 4762 | 25.32 | 293 | 100 |
| Imaging | 2503 | 13.31 | 263 | 89.76 |
| Consults | 2212 | 11.76 | 281 | 95.9 |
| Plan of Care | 1986 | 10.56 | 273 | 93.17 |
| RN Note | 1475 | 7.84 | 248 | 84.64 |
| Interdisciplinary | 1359 | 7.23 | 284 | 96.93 |
| Procedures | 588 | 3.13 | 265 | 90.44 |
| ECG | 410 | 2.18 | 115 | 39.25 |
| Patient Instructions | 370 | 1.97 | 194 | 66.21 |
| Assessment & Plan Note | 315 | 1.67 | 38 | 12.97 |
| Telephone Encounter | 311 | 1.65 | 146 | 49.83 |
| Discharge Summary | 266 | 1.41 | 239 | 81.57 |
| HNO_MYC_LET_INFO\|NOTE_ID | 257 | 1.37 | 79 | 26.96 |
| Discharge Instr - Other Orders | 225 | 1.2 | 206 | 70.31 |
| H&P | 143 | 0.76 | 93 | 31.74 |
| Discharge Instructions | 134 | 0.71 | 127 | 43.34 |
| Pathology and Cytology | 119 | 0.63 | 53 | 18.09 |
| Problem Overview | 107 | 0.57 | 33 | 11.26 |
| <NA> | 103 | 0.55 | 58 | 19.8 |
| Sticky Note | 94 | 0.5 | 61 | 20.82 |
| Consult | 93 | 0.49 | 70 | 23.89 |
| Letter | 82 | 0.44 | 54 | 18.43 |
| Echocardiography | 77 | 0.41 | 35 | 11.95 |
| Note associated with ED Event | 70 | 0.37 | 35 | 11.95 |
| Significant Event | 57 | 0.3 | 42 | 14.33 |
| ED Provider Notes | 47 | 0.25 | 41 | 13.99 |
| Neurology | 45 | 0.24 | 27 | 9.22 |
| Anesthesia Preprocedure Evalua | 45 | 0.24 | 36 | 12.29 |
| Lab | 44 | 0.23 | 18 | 6.14 |
| Anesthesia Post-Op Note | 43 | 0.23 | 37 | 12.63 |
| Care Coordination | 39 | 0.21 | 39 | 13.31 |
| PAT_SPEC_CMTS\|SPECIALTY_COM_ID | 34 | 0.18 | 34 | 11.6 |
| ED Information Exchange | 32 | 0.17 | 12 | 4.1 |
| Pre-Procedure Instructions | 32 | 0.17 | 28 | 9.56 |
| Imaging-Vasc | 31 | 0.16 | 10 | 3.41 |
| Face to Face | 29 | 0.15 | 27 | 9.22 |
| Anesthesia Procedure Notes | 28 | 0.15 | 25 | 8.53 |
| eConsult Inpatient | 26 | 0.14 | 12 | 4.1 |
| Operative Report | 25 | 0.13 | 22 | 7.51 |
| Interfacility | 24 | 0.13 | 9 | 3.07 |
| Consents | 17 | 0.09 | 17 | 5.8 |
| Nursing | 14 | 0.07 | 14 | 4.78 |
| Anesthesia Transfer of Care | 11 | 0.06 | ≤10 | ≤3.41 |
| ACP (Advance Care Planning) | ≤10 | ≤0.05 | ≤10 | ≤3.41 |
| IV | ≤10 | ≤0.05 | ≤10 | ≤3.41 |
| Group Note | ≤10 | ≤0.05 | ≤10 | ≤3.41 |
| Interval H&P Note | ≤10 | ≤0.05 | ≤10 | ≤3.41 |
| Discharge Instr - Meds | ≤10 | ≤0.05 | ≤10 | ≤3.41 |
| ED Triage Notes | ≤10 | ≤0.05 | ≤10 | ≤3.41 |
| Ophthalmology | ≤10 | ≤0.05 | ≤10 | ≤3.41 |
| H&P (View-Only) | ≤10 | ≤0.05 | ≤10 | ≤3.41 |
| Care Plan | ≤10 | ≤0.05 | ≤10 | ≤3.41 |
| Student Note | ≤10 | ≤0.05 | ≤10 | ≤3.41 |
| Point-of-Care Imaging | ≤10 | ≤0.05 | ≤10 | ≤3.41 |
| Brief Op Note | ≤10 | ≤0.05 | ≤10 | ≤3.41 |
| Letter that was edited | ≤10 | ≤0.05 | ≤10 | ≤3.41 |
| PFT | ≤10 | ≤0.05 | ≤10 | ≤3.41 |
| ECONSULT | ≤10 | ≤0.05 | ≤10 | ≤3.41 |
| Case Communication | ≤10 | ≤0.05 | ≤10 | ≤3.41 |
| Dialysis | ≤10 | ≤0.05 | ≤10 | ≤3.41 |
| Unmapped External Note | ≤10 | ≤0.05 | ≤10 | ≤3.41 |
| Imaging Consult | ≤10 | ≤0.05 | ≤10 | ≤3.41 |
| Falls | ≤10 | ≤0.05 | ≤10 | ≤3.41 |
| Microbiology | ≤10 | ≤0.05 | ≤10 | ≤3.41 |
| Discharge Instr - Activity | ≤10 | ≤0.05 | ≤10 | ≤3.41 |
| Code Documentation | ≤10 | ≤0.05 | ≤10 | ≤3.41 |
| PR Charge | ≤10 | ≤0.05 | ≤10 | ≤3.41 |
| GI | ≤10 | ≤0.05 | ≤10 | ≤3.41 |
| Outpatient Referral | ≤10 | ≤0.05 | ≤10 | ≤3.41 |
| Nursing Note | ≤10 | ≤0.05 | ≤10 | ≤3.41 |
| Transcription had in basket re | ≤10 | ≤0.05 | ≤10 | ≤3.41 |
| Treatment Summary | ≤10 | ≤0.05 | ≤10 | ≤3.41 |
| Note was attached to an IB Mes | ≤10 | ≤0.05 | ≤10 | ≤3.41 |
| Anesthesia Postprocedure Evalu | ≤10 | ≤0.05 | ≤10 | ≤3.41 |
| ED Notes | ≤10 | ≤0.05 | ≤10 | ≤3.41 |
