# Supplementary material for: Extracting adverse event nature, severity, timelines and resulting interventions from clinical notes of patients receiving CAR-T cell therapy using large language models
Source: PLOS Digit Health. 2026 Aug 27;5(8):e0001426. doi: 10.1371/journal.pdig.0001426 (PMC13521353; doi:10.1371/journal.pdig.0001426)
Supplement: Supplementary file 4 [file pdig.0001426.s006.docx]

S3 Table Performances of the Large Language Models for each prompt by reviewer Jordan Guillot (JG) and Arvind Suresh (AS)

|  | JG |  |  |  | AS |  |  |  | JG |  |  |  | AS |  |  |  | Cohen’s kappa |
| --- | --- | --- | --- | --- | --- | --- | --- | --- | --- | --- | --- | --- | --- | --- | --- | --- | --- |
|  | True Positive | True Negative | False Positive | False Negative | True Positive | True Negative | False Positive | False Negative | Accuracy | Precision | Recall | F1 Score | Accuracy | Precision | Recall | F1 Score |  |
| Prompt Prespecified AE |  |  |  |  |  |  |  |  |  |  |  |  |  |  |  |  |  |
| Nature | 114 | 8 | 46 | 10 | 85 | 10 | 75 | 3 | 0.69 | 0.71 | 0.92 | 0.80 | 0.55 | 0.53 | 0.97 | 0.69 | 0.401 |
| Date | 86 | 62 | 45 | 10 | 90 | 70 | 41 | 3 | 0.73 | 0.66 | 0.90 | 0.76 | 0.78 | 0.69 | 0.97 | 0.80 | 0.545 |
| Clinical intervention | 97 | 29 | 66 | 1 | 114 | 32 | 50 | 6 | 0.65 | 0.60 | 0.99 | 0.74 | 0.72 | 0.70 | 0.95 | 0.80 | 0.448 |
| Prompt Non Prespecified AE |  |  |  |  |  |  |  |  |  |  |  |  |  |  |  |  |  |
| Nature | 21 | 32 | 10 | 11 | 18 | 41 | 13 | 2 | 0.72 | 0.68 | 0.66 | 0.67 | 0.80 | 0.58 | 0.90 | 0.71 | 0.606 |
| Date | 12 | 42 | 10 | 11 | 13 | 45 | 9 | 2 | 0.72 | 0.55 | 0.52 | 0.53 | 0.84 | 0.59 | 0.87 | 0.70 | 0.634 |
| Clinical intervention | 16 | 46 | 11 | 1 | 18 | 45 | 9 | 2 | 0.84 | 0.59 | 0.94 | 0.73 | 0.85 | 0.67 | 0.90 | 0.77 | 0.681 |
| Prompt CRS |  |  |  |  |  |  |  |  |  |  |  |  |  |  |  |  |  |
| Presence | 26 | 19 | 5 | 0 | 28 | 19 | 3 | 0 | 0.90 | 0.84 | 1.0 | 0.91 | 0.94 | 0.90 | 1.0 | 0.95 | 0.857 |
| Grade | 36 | 19 | 1 | 2 | 36 | 19 | 0 | 1 | 0.95 | 0.97 | 0.95 | 0.96 | 0.98 | 1.0 | 0.97 | 0.99 | 0.961 |
| Date | 46 | 19 | 10 | 2 | 47 | 19 | 8 | 2 | 0.84 | 0.82 | 0.96 | 0.88 | 0.87 | 0.85 | 0.96 | 0.90 | 0.777 |
| Clinical intervention | 38 | 19 | 9 | 2 | 44 | 19 | 2 | 2 | 0.84 | 0.81 | 0.95 | 0.87 | 0.94 | 0.96 | 0.96 | 0.96 | 0.771 |
| Prompt ICANS |  |  |  |  |  |  |  |  |  |  |  |  |  |  |  |  |  |
| Presence | 7 | 39 | 3 | 1 | 6 | 38 | 4 | 2 | 0.92 | 0.70 | 0.88 | 0.78 | 0.88 | 0.60 | 0.75 | 0.67 | 0.792 |
| Grade | 10 | 39 | 2 | 1 | 8 | 38 | 4 | 2 | 0.94 | 0.83 | 0.91 | 0.87 | 0.88 | 0.67 | 0.80 | 0.73 | 0.862 |
| Date | 15 | 39 | 23 | 1 | 16 | 38 | 22 | 2 | 0.69 | 0.39 | 0.94 | 0.56 | 0.69 | 0.42 | 0.89 | 0.57 | 0.919 |
| Clinical intervention | 10 | 39 | 6 | 1 | 13 | 38 | 3 | 2 | 0.88 | 0.62 | 0.91 | 0.74 | 0.91 | 0.81 | 0.87 | 0.84 | 0.777 |
