# Supplementary material for: Extracting adverse event nature, severity, timelines and resulting interventions from clinical notes of patients receiving CAR-T cell therapy using large language models
Source: PLOS Digit Health. 2026 Aug 27;5(8):e0001426. doi: 10.1371/journal.pdig.0001426 (PMC13521353; doi:10.1371/journal.pdig.0001426)
Supplement: Supplementary file 5 [file pdig.0001426.s007.docx]

S4 Table Top 50 resulting clinical intervention for prespecified adverse events

| **Clinical intervention** | **Frequency (patients)** | **%** |
| --- | --- | --- |
| No transfusions needed today | 48 | 16.40% |
| Tocilizumab administration | 46 | 15.70% |
| Transfuse for Hb<7,plt<10k | 25 | 8.50% |
| Cefepime administration | 18 | 6.10% |
| Assess and record max CRS twice/day. If CRS develops,increase lab frequency to daily. | 17 | 5.80% |
| Continue levaquin and fluconazole until no longer neutropenic | 17 | 5.80% |
| Assess and record max ICANS score daily and prn. RN neuro checks q shift. | 17 | 5.80% |
| Transfusion Threshold: Hgb < 7,Plt < 10 | 16 | 5.50% |
| Administered ***** | 14 | 4.80% |
| Administered cefepime | 14 | 4.80% |
| Administered ***** x 1 | 13 | 4.40% |
| Transfuse to keep hgb>7 | 13 | 4.40% |
| Transfuse to keep plt >10 | 13 | 4.40% |
| Received ***** x 1 | 12 | 4.10% |
| Transfusion: Hgb >8,Platelet >10 | 12 | 4.10% |
| Administration of tocilizumab | 11 | 3.80% |
| Dexamethasone administration | 11 | 3.80% |
| No clinical intervention mentioned | 11 | 3.80% |
| No transfusions or cytokines needed today | 11 | 3.80% |
| No transfusions or cytokines needed today. | ≤10 | ≤3.4% |
| s/p IV Zosyn XX/XX/XX - XX/XX/XX in setting of neutropenic fevers | ≤10 | ≤3.4% |
| IV Zosyn XX/XX/XX - XX/XX/XX | ≤10 | ≤3.4% |
| No transfusions needed today. | ≤10 | ≤3.4% |
| Monitoring for signs/symptoms of CRS/Neurotoxicity | ≤10 | ≤3.4% |
| No specific intervention mentioned | ≤10 | ≤3.4% |
| Monitor for signs/symptoms of CRS/Neurotoxicity | ≤10 | ≤3.4% |
| Administered ***** x1 | ≤10 | ≤3.4% |
| Administration of ***** x1 | ≤10 | ≤3.4% |
| Platelet transfusion | ≤10 | ≤3.4% |
| Monitoring for fever,hypotension | ≤10 | ≤3.4% |
| Ceftriaxone administration | ≤10 | ≤3.4% |
| Transfuse PRBCs for Hgb<7 (or sx) or with bleeding using CMV negative,irradiated,and leukoreduced products. Premed with tylenol and atarax 10mg po | ≤10 | ≤3.4% |
| Patient at high risk for developing CRS or neurotoxicity | ≤10 | ≤3.4% |
| Started on cefepime | ≤10 | ≤3.4% |
| Started cefepime | ≤10 | ≤3.4% |
| Administered ***** and dexamethasone | ≤10 | ≤3.4% |
| Tocilizumab | ≤10 | ≤3.4% |
| Transfuse to keep hgb>7,plt >10 | ≤10 | ≤3.4% |
| Given ***** x1 | ≤10 | ≤3.4% |
| No transfusions today | ≤10 | ≤3.4% |
| Clinical monitoring | ≤10 | ≤3.4% |
| tocilizumab | ≤10 | ≤3.4% |
| No transfusions needed | ≤10 | ≤3.4% |
| Started on ***** 10mg IV Q6h on XX/XX/XX received a total of 3 doses then increased to 20mg IV Q6h doses on XX/XX/XX-> switched to Solumedrol 1000mg due to severe bradycardia XX/XX/XX dex (XX/XX/XX,XX/XX/XX,XX/XX/XX) | ≤10 | ≤3.4% |
| Transfuse with CMV negative,LR,IR products. For platelet transfusion needs acetaminophen,diphenhydramine,and famotidine for premedication; has history of hives with transfusions. No premeds with blood. Avoid hydrocortisone. | ≤10 | ≤3.4% |
| Heme goals HgB >7,plts >10K | ≤10 | ≤3.4% |
| Tylenol given | ≤10 | ≤3.4% |
| Cefepime 2g IV Q8h | ≤10 | ≤3.4% |
| Monitor for signs/symptoms of CRS/Neurotoxicity - Fever/Hypotension/Tachycardia/Hypoxia - Mental status changes - RN to cont BID neuro checks per study protocol - Oncologist to determine steroids vs ***** admin - Page fellow *****-***** with CRS/Neurotox symptoms | ≤10 | ≤3.4% |
| Cefepime 2g IV q8h | ≤10 | ≤3.4% |
