# Supplementary material for: Extracting adverse event nature, severity, timelines and resulting interventions from clinical notes of patients receiving CAR-T cell therapy using large language models
Source: PLOS Digit Health. 2026 Aug 27;5(8):e0001426. doi: 10.1371/journal.pdig.0001426 (PMC13521353; doi:10.1371/journal.pdig.0001426)
Supplement: Supplementary file 6 [file pdig.0001426.s008.docx]

S5 Table Category of Non-Prespecified Adverse Events as describe in the results

ae_categories = {

"Fever & Inflammatory Responses": [

"Fever", "Febrile", "Intermittent fevers", "Persistent fevers",

"Rigors", "Mild chills", "Fevers and tachycardia",

"Cytokine Release Syndrome (CRS)", "Grade 4 CRS",

"Recurrent fever", "Transient fever", "Febrile overnight",

"Low grade temp 99.3", "Fever and neutropenia",

"Febrile and tachycardic", "Transient rigors",

"Neutropenic fever", "Elevated inflammatory markers",

"Febrile to Tmax 39.3C overnight",

"Mild fever",

"Shivering",

"Rising CRP",

"Intermittent fevers + hypotension to 72/46"

],

"Gastrointestinal Issues": [

"Nausea", "Nausea and vomiting", "Diarrhea", "Watery diarrhea",

"Loose stool", "Toxic enterocolitis", "Esophagitis",

"Dyspepsia/bloating/gas", "Severe constipation",

"C difficile infection", "Toxic Gastroenteritis", "Proctitis",

"Abdominal pain", "Diffuse abdominal pain", "GI bleed",

"Lower GI bleed", "Acute diarrhea",

"Hemorrhagic UGIB", "Constipation", "Severe nausea",

"Persistent diarrhea", "Enterocolitis due to C-Diff infection",

"Toxic enteritis", "Bloody diarrhea ? hematuria",

"Diarrhea, resolved", "Intermittent diarrhea",

"Abdominal tenderness", "Toxic enterocolitis secondary to chemotherapy",

"Dysphagia", "Nausea/mild epigastric discomfort",

"Solid-food dysphagia", "Chronic diarrhea",

"Severe diarrhea", "Constipation-resolved", "Enterocolitis due to chemotherapy",

"Abdominal pain and rectal pain", "Toxic gastroenteritis",

"Diffuse abdominal tenderness", "Stercoral-colitis",

"Recurrent small bowel obstruction", "Acute pancreatitis",

"Dysgeusia", "Toxic Enteritis",

"Chronic Diarrhea",

"Abdominal Pain",

"Recurrent GI Bleed",

"Diarrhea",

"Diarrhea,resolved",

"Diarrhea/loose stools",

"Odynophagia",

"Ischemic colitis",

"Acalculous cholecystitis",

"Pelvic abscess",

"Prolapse",

"Contained perforation adjacent to the anastomosis",

"Small abscesses",

"Hemorrhagic cystitis",

"Rectal pain",

"Severe Constipation",

"Abd pain and rectal pain",

"LLQ abdominal pain",

"Mild constipation",

"New abdominal tenderness",

"Soft stools",

"Esophageal HSV",

"Altered bowel habits",

"Severe dysphagia and severe protein/calorie malnutrition",

"Hemorrhoids",

"Constipation,resolved",

"Acinetobacter UTI c/b Hematuria from R nephrostomy tube",

"Nausea & vomiting",

"Nausea/vomiting",

"Nausea and Vomiting",

"Nausea and emesis",

"Nausea/Vomitting",

"Emesis with scant blood tinge",

"Acute on chronic extremity swelling due to volume overload",

"Pneumoperitoneum",

"Possibly cholecystitis",

"Elevated LFT",

"Gingival bleeding",

"Vaginal bleeding",

"Non-gap metabolic acidosis, improving",

"Shock Liver",

"Poor PO intake",

"Malnutrition",

"Volume overload",

"*****: Hemorrhagic UGIB",

"Metabolic acidosis",

"Orbital cellulitis",

"Oral thrush",

"Hypofibrinogenemia",

"Acid reflux",

"Hyperbilirubinemia",

"Transaminitis",

"Cholestatic/hepatocellular liver injury",

"Nausea/Vomiting",

"Elevated Transaminases",

"Worsening LFTs",

"Low Fibrinogen",

"Abnormal brain CT",

"Aspiration event",

"Dyspepsia",

"Anorexia, Dysgeusia (taste alteration), Nausea/Vomiting",

'Elevated LFTs', 'abdominal Pain', 'diarrhea', 'Intermittent nausea', 'Severe abdominal pain',

'GERD', 'IBS/GERD', 'History of pancreatitis', 'Disorder of nutrition',

'Hemorrhagic Chemosis', 'Anorexia,Dysgeusia (taste alteration),Nausea/Vomitting',

'Glucose trending up as steroids restarted', 'UGIB - resolved', 'UGIB',

"Hiccups",

"Emesis",

"N/V",

'transaminitis' ,

"LFT elevation",

],

"Neurological Issues and Dizziness": [

"Acute encephalopathy", "Neurotoxicity", "Altered mental status",

"Confusion", "Headache", "Persistent chronic headache", "Seizure activity",

"Status epilepticus", "Migraine", "Mild neuropathy", "Neuropathy",

"Grade 4 Neurotoxicity", "Cognitive impairment", "Personality changes",

"Movement disorder", "Visual disturbances", "Retinal Hemorrhages",

"Migraine and blackout vision", "Optic nerve edema",

"Memory loss", "Unsteady gait", "Mild neurotoxicity",

"Acute encephalopathy, possible seizure", "Acute visual disturbance",

"Brain fog", "New AMS overnight", "Acute Encephalopathy 2/2 Grade 2 Neurotoxicity",

"Seizure", "Seizure like activity", "Essential Tremor vs myoclonus",

"Hypnopompic hallucinations", "Slow speech and takes time to find words",

"Difficulty choosing the right words", "Transient vertigo",

"Facial neuro deficits", "Acute stroke", "New ischemic stroke",

"Migraine and black out vision", "Acute metabolic encephalopathy",

"Acute stroke, small", "Worsening mental status",

"Severe headache", "Hand tremor", "Dizziness,Hypotension",

"Brain AVF", "Acute encephalopathy,hand tremor",

"Acute encephalopathy,possible seizure", "Acute Retinal Hemorrhages",

"Persistent headache", "New hallucinations", "Hallucination",

"Seizures", "Symptomatic orthostasis", "Neuropathic pain",

"Visual floaters in bilateral eyes", "Binocular Diplopia",

"Headache,progressive", "Numbness/Twitching in hands and feet",

"Blurred vision", "Acute distress","Confusion,gait changes,and mild bilateral hand tremors",

"Impaired Vision",

"Decrease in hearing",

"Encephalopathy: Metabolic evidenced by delirium",

"Encephalopathy and BLE weakness",

"Encephalopathy due to *****-T neurotoxicity,delirium",

"Toxic Encephalopathy",

"At risk for CRS/Neurotoxicity",

"Grade 2 Encephalopathy",

"Neurotoxicity req steroids",

"Developing hydrocephalus",

"Obstructive hydrocephalus",

"Chronic trichiasis",

"Episode of blacked out vision with headache",

"AMS",

"Persistent nausea",

"Seizure like activity/encephalopathy",

"Grade ***** *****",

"Chorioretinopathy,bilateral",

"Vision loss",

"Permanent optic nerve damage",

"Right sided facial droop",

"R sided facial droop",

"Minor tremor in hands",

"Essential tremor",

"Stable hand tremors",

"Increased tremor",

"Hand tremors",

"Headaches",

"Mild headache",

"Ocular headache",

"Somnolence",

"Late morning drowsiness",

"Sleeping trouble",

"Syncopal episode",

"Syncopal event",

"Dizziness",

"Dizziness with ambulation",

"Orthostatic dizziness",

"Dizziness,risk of *****",

"Dizziness with standing",

"Symptomatic orthostatic hypotension",

"Orthostatic hypotension/dizziness",

"Deconditioned",

"Deconditioned from prolonged/multiple hospital stays",

"Hypoxemia - intermittent",

"High risk for falls",

"Blacked out vision and headache",

"Left chin numbness",

"L chin numbness",

"Pathologic L humeral fracture",

"Splenic infarct",

"Intermittent shin pain",

"Dizziness", "Lightheadedness", "Loss of consciousness",

"Dizziness with ambulation", "Orthostatic dizziness", "Dizziness & orthostasis",

"Lightheadedness after standing", "Dizziness and feeling high after low dose marinol",

"Symptomatic orthostatics",

"Orthostatic dizziness",

"Symptomatic orthostatic hypotension",

"Syncope",

"Delirium",

"At risk for CRS/Neurotoxicity",

"Tinnitus",

"Possible seizure",

"Acute stroke, small",

"Encephalopathy",

"Peripheral neuropathy",

"Severe agitation",

"Encephalitis",

"CRS/neurotoxicity",

"Vertigo",

"Metabolic Encephalopathy",

"Treating encephalopathy",

"Numbness in feet",

"Left shoulder and bicep pain",

"Syncopal episode with head trauma",

"Pain: R neck and shoulder pain from neck mass",

"Cognition",

"Generalized body aches",

"Full body aches",

"Restless leg syndrome",

"Pain of left thigh",

"Peripheral neuropathy due to chemotherapy",

"Chronic neuropathic pain",

"Left Upper Extremity Pain",

"Throat tightness and swelling under tongue",

"Intermittent hematuria",

'Acute stroke,small', 'Left foot drop', 'Subdural hematoma', 'Chronic cancer-related pain',

'Vocal cord atrophy/paralysis of LEFT true vocal cord', 'Post-herpetic neuralgia', 'Cauda equina syndrome',

'Seizure XX/XX/XX lymphoma\xa0in ***** XX/XX/XX, 'Mild occipital headache',

'Encephalopathy: Toxic Encephalopathy', 'Non-infectious SIRS due to *****-T cell therapy with End ***** Damage present (toxic encephalopathy)',

'Patient passed out', 'Bilateral visual loss from optic nerve atrophy due to leukemic involvement',

'Back pain', 'Restless Legs', 'Pain at port site', 'Chronic cancer pain', 'R>L shoulder pain',

"Vision loss of right eye",

"Blurry vision",

"Right frontal extra-axial collection",

"Mild PN",

"***** changes",

"Hx SDH",

"SDH"

],

"Cardiovascular Issues": [

"Hypertension", "Hypotension", "Orthostatic hypotension",

"Orthostatic tachycardia", "Bradycardia", "Sinus bradycardia",

"Tachycardia", "Prolonged QTC", "Afib with RVR",

"Paroxysmal Atrial Fibrillation", "VTach overnight",

"Saddle Pulmonary Embolus", "Orthostatic HoTN", "Sinus tachycardia",

"Atrial Tachycardia", "Transient Afib w/ RVR", "Paroxysmal SVT",

"A flutter", "Prolonged QTc", "Mild persistent neuropathy",

"Transient ***** potentially c/w grade 2 neurotoxicity",

"Tachy-brady", "Sinus Tachycardia", "New onset Afib",

"Paroxysmal Atrial Fibrillation c/b RVR",

"Low Blood Pressure",

"Hypotension",

"Orthostatic hypotention",

"Orthostatic Hypotension",

"Hypotensive to 82/49",

"hypotensive to 72/46",

"Sinus Bradycardia",

"A fib with RVR",

"Afib w/ RVR",

"Atrial tachycardia",

"VTach overnight and 6 beats this AM",

"Multiple PVCs",

"4 seconds atrial tach",

"QTc prolonged",

"Risk of QTc prolongation",

"Prolonged Qtc",

"Prolonged PT",

"High D-dimer",

"Elevated d-dimer",

"Tachycardia up to 130 with ambulation",

"Tachycardia / anxiety",

"Heartburn",

"Chest pain",

"Chest pain during ***** infusion",

"Chest Pain/SOB",

"Chest Pain/SOB/Orthostasis",

"Chest Pain/SOB/*****",

"Acute Saddle PE",

"PE",

"Incidental PE",

"Hx of Pulmonary embolism/SMV thrombus",

"Hx of Pulmonary embolism/SMV thrombus s/p anticoagulation",

"Saddle PE",

"Multiple pulmonary emboli",

"Pericardial effusion",

"Pericardial effusion ?malignant",

"Possibly left ovarian vein thrombus",

"Nonocclusive right jugular venous thrombosis",

"Non-occlusive right axillary DVT",

"Non-occlusive DVT in R axillary vein",

"Non-occlusive DVT of RUE",

"DVT",

"DVT of RLE",

"LLE DVT",

"RUE DVT",

"R IJ Port associated thrombus",

"R malignant pleural effusion",

"Bilateral pleural effusions",

"Bilateral pleural effusions R>L ?malignant",

"CKD stage III",

"Hx of incidental PE",

"hx of incidental PE",

"Hx of ARF",

"HTN",

"OSA",

"Septic shock",

"Mild pulmonary edema",

"Risk for vision loss",

"Hemoptysis",

"Hypoxemia",

"Hypercarbia",

"R pleural effusion",

"Hx of HTN",

"Hx of paroxysmal SVT",

"Hx of AT",

"Bradycardia during ***** infusion",

"Non ischemic cardiomyopathy",

"HTN established, controlled",

"Hypotensive",

"Orthostasis",

"Aflutter",

"Paroxysmal Atrial Fibrillation c/b RVR now resolved",

"Chronic Sinus bradycardia",

"Asymptomatic Baseline Hypotension",

"Prior CDI",

'hx of HTN', 'Rule out PE', 'At risk for CRS/ Neurotoxicity', 'Hx of possible pericardial involvement by lymphoma',

'VTE', 'Hypertension,controlled', 'HTN established,controlled', 'HTN,Established,uncontrolled',

'Chronic RIJ Occlusive thrombus', 'H/o Splenic infarct', 'Bilateral pleural effusions R>L\xa0?malignant',

'Pericardial effusion\xa0?malignant', 'Low BP', 'VTE PPx: Contraindicated: CNS mets',

'Asymptomatic Bradycardia', 'intermittent fevers + hypotension to 72/46', 'At risk for CRS/Neurotoxicity after *****-T infusion',

'HCM', 'Acute on chronic subdural bleed', 'Capillary leak', 'R IJ/R Subclavian DVT related to ***** catheter',

'LLE DVT s/p IVC filter', 'Deep Vein Thrombosis', 'Methemoglobinemia',

'Chronic RIJ Occlusive thrombus', 'H/o Splenic infarct',

"***** w/ RVR overnight",

"***** w/ RVR",

"Hx of HFpEF",

"Hx DVT",

"Hx of incidental PE",

],

"Respiratory Issues": [

"Dyspnea", "Hypoxia", "Acute hypoxic respiratory failure",

"Respiratory distress", "Pulmonary edema", "Asthma",

"Obstructive Hydrocephalus", "Pneumocystis Jirovecii Pneumonia infection",

"Pulmonary aspergillosis", "Hypoxic respiratory failure",

"Acute dyspnea", "Acute hypoxia", "Respiratory failure",

"Respiratory failure: Acute with hypoxemia", "Multifocal GGOs, C/f PNA",

"Pulmonary infection", "Oxygen requirement",

"Increased dyspnea on exertion and increased fatigue",

"Respiratory distress in setting of fluid overload",

"Cough,Rhinovirus +", "Dyspnea on exertion",

"Shortness of breath",

"Hypoxia - intermittent",

"Acute on chronic hypoxic respiratory failure",

"***** hypoxic respiratory failure",

"Acute on chronic hypoxic respiratory failure",

"Acute Saddle PE","Cough",

"OSA treated with BiPAP",

"Obstructive sleep apnea",

"Bilateral pleural effusions R>L ?malignant",

"Pericardial effusion ?malignant",

"Multifocal GGOs, C/f PNA",

"c/f PNA",

'Increased dyspnea (now at rest) and ***** ***** ***** ***** *****% while sitting',

'Obstructive sleep apnea on CPAP', 'Severe OSA', 'Multifocal GGOs,C/f PNA', 'Sinus infection',

'Sinus headaches', 'Bilateral conjunctivitis', 'Bilateral conjunctivitis/Hemorrhagic Chemosis',

'Mild edema of epiglottis and arytenoids',

"Sore throat"

],

"Renal & Electrolyte Issues": [

"Acute kidney injury (AKI)", "AKI on CKD",

"Acute kidney injury on CKD III", "Microscopic hematuria",

"Hyperkalemia", "Hypokalemia", "Hyponatremia", "Hypercalcemia",

"Low Urea Nitrogen", "Elevated Creatinine", "Renal function impairment",

"Hematuria", "Persistent lactic acidosis", "Non-gap metabolic acidosis",

"Non-gap metabolic acidosis, improving", "AKI due to postobstructive uropathy",

"Hypomagnesemia", "Low Potassium", "High Chloride", "Low CO2",

"Low Calcium", "Low Albumin", "Low Phosphorus",

"Acute kidney injury on CKD III", "Acute hyponatremia",

"Renal Failure", "AKI,due to postobstructive uropathy",

"Increased Creatinine", "Acute Kidney Injury",

"Acute kidney injury (AKI)", "AKI (Established,improved)",

"Renal abscess", "AKI due to interval worsening B hydronephrosis due to malignancy",

"Recurrent AKI","AKI",

"Acute Kidney Injury",

"Acute Kidney Injury (AKI)",

"Acute kidney injury",

"Hx of ARF",

"Non-gap metabolic acidosis, improving",

"Hyperkalemia",

"Mild Hyponatremia",

"Polyuria",

"Urinary urgency",

"Urinary hesitancy",

"AKI (baseline Cr ~1)",

"Cr elevated to 1.50",

"Hypoalbuminemia",

"R ureteral obstruction",

"AKI, R ureteral obstruction",

'ARF', 'Cr elevated-1L NS IVF bolus given', 'HYPERkalemia', 'Urinary retention', 'CKD',

'Low Uric Acid', 'Low Creatine kinase', 'Low ALT', 'High Direct Bilirubin', 'High Ferritin',

'Low Abs Neutrophils', 'Low Abs Lymphocytes', 'Low Abs Monocytes', 'Low Fibrinogen,Functional',

'High Fibrin D-Dimers', 'Hypernatremia', 'Urinary frequency', 'CKD XX/XX/XX renal lymphoma involvement',

'Chronic Kidney Disease: CKD Stage IIIa', 'AKI,R ureteral obstruction', 'Cystitis symptoms',

'Non-gap metabolic acidosis,improving',

"CKD stage",

"Hyperlipidemia",

"Hyperlipidemia, controlled",

'Hyperlipidemia,controlled',

"Hx of hyperuricemia",

"Incontinence",

'Urology - increased nocturia'

],

"Infections": [

"Bacterial infection", "Viral infection", "Fungal infection",

"Zoster Infection", "Staph epi bacteremia", "Pseudomonas bacteremia",

"Strep Mitis bacteremia", "Acinetobacter UTI", "C diff colitis",

"Vaginitis", "Urine Cx: >100,000 Staph Epi", "Odontogenic infection",

"COVID infection", "COVID Viral infection", "COVID-19 infection",

"COVID-19 infection, recovered", "RSV", "HHV-6 infection",

"Ecoli bacteremia", "ESBL UTI", "Stomatitis, presumed fungal",

"Vaginitis with vaginal white papules", "Sepsis", "Septal resection site with fungal elements",

"Skin infection at PICC site", "Fungemia", "Septic embolization",

"Achromobacter bacteremia", "Acute COVID infection",

"Non-neutropenic fever", "Non-neutropenic fever,suspected pulmonary source (resolved)",

"COVID + infection", "Strep mitis bacteremia",

"Rhino positive", "Septic shock due to GNR bacteria",

"Rhinovirus infection",

"COVID Infection",

"COVID Viral infection",

"COVID-19 infection,recovered",

"Staph epi central line infection/colonization",

"C. diff",

"C. Diff colitis",

"CDiff colitis",

"Recurrence of C. Diff colitis",

"Odontogenic infection",

"MDR pseudomonas and E faecium complicated UTI",

"C.diff",

"Klebsiella CLABSI/bacteroides fragilis bacteremia",

"Orbital cellulitis",

"UTI",

"GPC bacteremia",

"Cervical adenopathy (Left cervical neck mass)",

"Stomatitis, presumed fungal",

"External otitis media",

"Cytomegalovirus (CMV) viremia",

"Hx Osteomyelitis s/p resection and abx spacers",

"Osteomyelitis",

"Hx of COVID Viral infection",

"L axillary wound",

'Orbital cellulitis R>L', 'hx of Oral thrush', 'c/f SSTI / superinfection of chest wall mass',

'Reported hx of PSC,Established,controlled', 'H/o COVID Infection', 'Treated for sepsis',

'Hematuria,resolved', 'Stomatitis,presumed fungal', 'Sinus infection',

"Prostatic abscess",

"Hepatitis B core antibody positive",

"Complex liver cyst with hemorrhage"

],

"Hematologic & Oncologic Issues": [

"Pancytopenia", "Cytopenias", "Neutropenia",

"Hemophagocytic Lymphohistiocytosis (HLH)",

"Macrophage Activation Syndrome (MAS)", "Tumor Lysis Syndrome",

"Refractory B-Cell Lymphoma", "Malignancy associated fatigue",

"Secondary myeloid malignancies", "Anemia associated with malignancy",

"Neoplasm/malignancy associated pain", "Pain due to leukemia",

"Malignancy-associated pain", "Cancer Related Pain", "Relapsed diffuse large B-cell lymphoma",

"Thrombocytopenia secondary to malignancy", "Bone disease",

"Advanced Care Planning", "Relapsed DLBCL",

"B-cell ALL,s/p XX/XX/XX MUD BMT XX/XX/XX",

"Refractory *****-Cell Lymphoma", "Pancytopenia due to chemotherapy",

"Functional SBO due to abdominal lymphomatosis",

"Recurrent malignant ascites", "Primary refractory high-grade B-cell lymphoma (double hit) with CNS involvement",

"Large B-cell Lymphoma,Stage IV,Refractory",

"Disease associated pancytopenia", "Profound thrombocytopenia",

"Pancytopenia XX/XX/XX disease", "Multiple Myeloma",

"Chronic Neuropathy", "Neoplasm/malignancy associated fatigue",

"Pain,Malignancy-associated",

"pancytopenia",

"Cytopenia",

"Anemia due to malignancy",

"Cancer-related pain",

"Malignancy associated fatigue on admission",

"Relapsed DLBCL,non-GCB",

"Refractory DLBCL",

"B-cell ALL",

"Coagulopathy",

"At risk of tumor lysis syndrome",

"Possible pericardial involvement by lymphoma",

"Adrenal insufficiency (CMS code)",

"Acute lymphoblastic leukemia",

"Possible MDS",

"Proliferative Diabetic Retinopathy",

"Platelet refractory",

"Pancytopenia due to lymphodepleting chemotherapy",

"Profound leukocytosis",

"Thyroid nodule",

"Cancer Pain",

"Severe protein-calorie malnutrition",'Low WBC Count', 'Low Hemoglobin', 'Low Hematocrit', 'Low RBC Count', 'Low Platelet Count',

'Low ***** Count,Flow Cytometry', 'High PT', 'High INR', 'High aPTT', 'Athlete\'s foot',

'***** ALL (acute lymphoblastic leukemia)', 'ALL (acute lymphoblastic leukemia)',

'Non-infectious SIRS due to *****-T cell therapy', 'Throat itching', 'Failure to thrive,malnutrition',

'Bilateral foveal subretinal fluid/serous detachment,consistent with very mild central serous retinopathy',

'Abnormal mandibular marrow signal adjacent to left trigeminal nerve in the mandibular foramen',

'Lytic bone disease',

"LT groin ulcer",

"Hx R humerus fracture"

],

"Skin & Soft Tissue Issues": [

"Rash", "Papular rash", "Skin Rash", "Pruritis", "Conjunctivitis",

"Diffuse Body Erythema", "RUE swelling and pruritis",

"Upper back erythema", "Intertrigo", "Diffuse Erythema",

"Erythematous papular rash to face", "Rash on back",

"Pruritic back rash", "Rash on arms, back", "Skin changes over hands",

"Maculopapular rashes", "Full body rash", "Bleeding from back",

"Radiation dermatitis", "Non-infectious SIRS due to supraglottic arytenoid and epiglottic edema",

"New rash", "Skin lesions", "Rash present",

"Maculopapular rashes on the chest and forehead",

"Rash associated with CAR-T infusion", "Rash on legs and hands",

"Diffuse skin changes", "Erythematous follicular papules","Rash on arms,back",

"Skin rash",

"Diffuse skin changes",

"Pruritus",

"Dry skin over hands/feet",

"Dry skin/dry mouth",

"Dry eye",

"Abrasion on the right chest from previous dressing",

"Dried blood noted",

"Facial/eye swelling",

"Submandibular swelling",

"Submandibular gland swelling",

"Ingrown toenail",

"Rash, resolving",

"Gluteal muscle soreness",

"Bilateral eye swelling",

"Perirectal abrasions",

"L axillary wound",

"Right first toe injury with scar formation",'***** lower extremity pain', 'L groin pain', 'Achy', 'Hypothermia', 'Rash,resolving',

'Swelling (right hand is swollen,not erythematous,non tender. R arm with edema that is also not tender.)',

'Cervical adenopathy ( Left cervical neck mass)', 'Superficial venous thrombosis', 'Left upper chest pain',

'Small amount of bleeding at port site',

"***** swelling",

"LT groin ulcer"

],

"Musculoskeletal Issues": [

"Bone pain", "Back pain", 'back pain', "Chronic Back pain", "Shoulder pain",

"L Ankle pain", "LLE weakness/pain", "Sacral pain due to herniated disc",

"Pain in legs and hips", "New onset back pain", "Diffuse Cerebral Microhemorrhages",

"Pathologic right femoral neck fracture", "Impending Left humeral fracture",

"Painful sensory neuropathy and tremors", "Severe sinus Bradycardia",

"Left sided TMJ pain", "R hip replacement", "Shoulder pain",

"Severe R temporal pain", "Pain management", "Left shoulder pain",

"Neck and R hip pain", "L Groin pain", "Back/left hip pain",

"Bilateral LE neuropathy", "RUE pain", "R lower cheek melanoma",

"Pain (primarily back and leg)", "Right shoulder pain",

"Acute back pain", "Bone pain: low back,hips,legs","Musculoskeletal Issues",

"Chronic pain",

"Chronic back pain",

"Low back pain",

"Back pain due to radiculopathy",

"Muscle cramping",

"Muscle weakness",

"Generalized pain to chest wall",

"Weakness in the legs",

"Right foot 5th metatarsal fracture",

"Hand cramping",

"Pain of left thigh",

"Joint pain",

"Left shoulder strain",

"Supraspinatus tear and tendinosis",

"Multiple vertebral osseous lesions",

"Frequent falls",

"Physical Deconditioning",

"Back pain",

"Bilateral foot neuropathy",

"Fall",

'L lower extremity pain', 'Crampy muscle spasms', 'Persistent left shoulder and bicep pain',

'Bilateral feet neuropathy', 'Right hand index finger fracture',

'Impaired mobility',

"Gout flair",

"Gout flair R hallux",

"Back pain",

"Hx of R humerus fracture"

],

"Psychiatric & Behavioral Issues": [

"Anxiety", "Depression", "Agitation", "Panic attack", "Insomnia",

"Increased somnolence", "Hallucinations", "Personality changes",

"Depression/anxiety", "Adjustment disorder w/ anxiety",

"Severe Depression", "Depressed mood and affect",

"Significant fatigue", "Anxiety about health",

"Major depressive disorder,single episode,moderate",

"H/o Passive Suicidal Ideation-resolved", "Anxiety/insomnia",

"Insomnia; Adjustment disorder w/ anxiety","Psych",

"Anxiety",

"Agitation",

"Depression",

"Adjustment disorder",

"Anxiety/Insomnia",

"Hx of Depression",

"Severe agitation",

"Restless leg syndrome",

'Pain', 'Drenching night sweats', 'Chills', 'Vasovagal episode',

"*****'s Disease"

],

"Endocrine Issues": [

"Adrenal insufficiency", "Diabetes Mellitus (T2DM)", "Hyperglycemia",

"Hypoglycemia", "Steroid-induced hyperglycemia", "Steroid-induced hypertension",

"Partial adrenal insufficiency", "Steroid induced hyperglycemia",

"Fasting hyperglycemia", "Secondary adrenal insufficiency",

"Steroid-induced hypertension", "Not well controlled DMII","Diabetes mellitus",

"Refractory hypoglycemia",

"Adrenal Insufficiency",

"Steroid-induced side effects",

"Hypothyroidism",

"Hx of Hypoglycemia, DM2",

"Adrenal *****",

"Hypoalbuminemia",

'Hypothyroid', 'H/o Hypoglycemia,DM2', 'T2DM', 'DM: Established,controlled'

],

"Immune System Issues": [

"Immunocompromised", "B-cell aplasia", "Graft-versus-host disease (GVHD)",

"CD4 lymphopenia", "Hypogammaglobulinemia", "Immunodeficiency",

"Functional immunosuppression", "Anaphylactic shock",

"Immunocompromised state", "Immune-related fatigue","Immunocompromised State",

"Neutropenic precautions",

"High C-Reactive Protein",

"Cancer related pain",

"Perineural spread of tumor",

'At risk for CRS/ Neurotoxicity', 'At risk of CRS/Neurotoxicity', '*****-T therapy requiring close monitoring for CRS/neurotoxicity',

"Allergies"

],

"Medication Side Effects": [

"Cefepime toxicity", "Steroid-induced side effects",

"Chemotherapy-related pancytopenia", "Toxic enteritis secondary to chemotherapy",

"Anaphylaxis", "Allergic reaction to Cefepime",

"Toxic gastroenteritis of chemotherapy", "Chemotherapy induced pancytopenia",

"Chemotherapy induced nausea and vomiting", "Toxic Enteritis","Methotrexate toxicity",

"Opioid induced constipation",

"Treating encephalopathy with increased dex",

"Decreased mealtime insulin",

'Glucose trending up as steroids restarted', 'Methemoglobinemia'

],

"Fatigue and weakness": [

"Fatigue", "Weakness", "Generalized weakness, fatigue and nausea",

"Exhaustion", "Chronic fatigue", "Fatigue/Dizziness",

"Deconditioning", "Fatigue/weakness", "Profound fatigue",

"Fatigue and Lethargy", "Extreme fatigue with cytoxan",

"General fatigue", "Muscle aches,profound fatigue,insomnia,bruising and anxiety",

"Acute on Chronic Fatigue,Generalized Weakness", "Low energy",

"Low energy level",

"Generalized weakness,fatigue and nausea",

"Fatigue",

"Generalized body aches",

"Full body aches",

"Ill-appearing",

'Fatigue and weakness', 'Weight loss', 'Weight gain',

"Mild pain near the kidney regions",

"Mild *****"

],

"Appetite loss and poor oral intake": [

"Appetite loss", "Poor oral intake",

"Decreased appetite", "Anorexia", "Early satiety", "Loss of appetite",

"Low appetite", "Poor appetite", "Decreased oral intake",

"Low appetite,weight loss",

"Decreased appetite/weight loss",

"Poor PO intake",

"Malnutrition",

"Lower appetite",

"Failure to thrive, malnutrition",

"Nutritional assessment",

'Insufficient calorie intake', 'Failure to thrive,malnutrition'

],

"Edema": [

"Edema", "Bilateral LE edema", "LLE Edema", "Trace edema to BLE's with L >R",

"Bilateral pleural effusions R>L", "Orbital edema", "Scrotal Edema",

"Anasarca", "Anasarca RUE", "Anasarca RUE with pitting edema",

"Edema in Right lower leg", "R hand swelling", "R arm swelling",

"Bilateral pleural effusions R>L",

"Scrotal,LE edema",

"Anasarca",

"Anasarca RUE with pitting edema but other extremities with no pitting edema but do appear to gradually swell",

"Bilateral edema",

"Mild fluid overload",

"Swelling (right hand is swollen, not erythematous, non tender. R arm with edema that is also not tender.)",

"Leg edema",

"Acute on chronic extremity swelling", 'RUE swelling', 'L arm swelling and discomfort distal to PICC', 'Bilateral pleural effusions R>L\xa0?malignant'

],

"Other AEs": [

"Elderly", "Hx weight loss", "Social", "Advanced Care Planning",

"Complex sx management", "Requires assistance with activities of rehabilitation",

"Developmental delay", "Distributive shock",

"Disease progression", "Nutritional Assessment",

"Fluid/Electrolytes", "Bone health", "Obesity",

"Fluid overload",

"Fluid status: Volume overload",

"Volume overload requiring IV diuresis",

"Protein calorie malnutrition due to SBO",

"No",

"Glaucoma",

"HLD",

"S/p cataract surgery L eye",

"Hair thinning",

"Sensitivity at tongue",

"Bowel and bladder incontinence",

"Episode of fecal incontinence",

'??cecal mass', 'Parotitis', 'BPH established,controlled', 'BPH', 'Hearing Changes', 'Tooth pain',

'Pain at port site',

"Nan",

"nan",

np.nan

]

}
