# Supplementary material for: Extracting adverse event nature, severity, timelines and resulting interventions from clinical notes of patients receiving CAR-T cell therapy using large language models
Source: PLOS Digit Health. 2026 Aug 27;5(8):e0001426. doi: 10.1371/journal.pdig.0001426 (PMC13521353; doi:10.1371/journal.pdig.0001426)
Supplement: Supplementary file 7 [file pdig.0001426.s009.docx]

S6 Table Top 50 Clinical Intervention for Non-Prespecified Adverse Events

| **Clinical intervention** | **Frequency (patients)** | **Percent** |
| --- | --- | --- |
| CTM | ≤ 10 | ≤ 3.4% |
| Correct with bicarbonate supplementation in ***** | ≤ 10 | ≤ 3.4% |
| Received third-line R-***** C1D1 XX/XX/XX | ≤ 10 | ≤ 3.4% |
| Continuous tele | ≤ 10 | ≤ 3.4% |
| Zofran RTC,compazine,Ativan PRN | ≤ 10 | ≤ 3.4% |
| Lidocaine patch to R shoulder,Oxycodone 2.5mg q6h PRN,PT/OT | ≤ 10 | ≤ 3.4% |
| Currently on IV abx | ≤ 10 | ≤ 3.4% |
| Zofran,compazine,Ativan PRN | ≤ 10 | ≤ 3.4% |
| Senna 17.2mg PO BID,Imodium prn,Miralax prn | ≤ 10 | ≤ 3.4% |
| Started on H1/H2 blockade,unasyn,vancomycin | ≤ 10 | ≤ 3.4% |
| Hospitalization | ≤ 10 | ≤ 3.4% |
| Cardiology consult,Monitor closely on telemetry,Stop offending medications,including zofran and dex,Steroids now switched from ***** to Solumedrol,although solumedrol can also cause bradycardia,Changed Fluc to caspo on XX/XX/XX | ≤ 10 | ≤ 3.4% |
| Empiric treatment for esophageal HSV,Fluconazole,Nystatin | ≤ 10 | ≤ 3.4% |
| dex 10mg q 6 hrs ( XX/XX/XX-XX/XX/XX)--> 10mg q 8hr (XX/XX/XX) --> 10mg q 12 (XX/XX/XX- ) | ≤ 10 | ≤ 3.4% |
| Now off ***** | ≤ 10 | ≤ 3.4% |
| Senna,Imodium,Miralax | ≤ 10 | ≤ 3.4% |
| CTM,Avoiding zofran,Use steroids cautiously if needed | ≤ 10 | ≤ 3.4% |
| Increased levetiracetam to 1500 mg BID | ≤ 10 | ≤ 3.4% |
| Midodrine 5 mg PO TID,1L NS bolus x1,mIVF @ 100ml/hr | ≤ 10 | ≤ 3.4% |
| Dexamethasone administration | ≤ 10 | ≤ 3.4% |
| Sent to ED for infectious workup | ≤ 10 | ≤ 3.4% |
| Increased levetiracetam 1500 mg BID | ≤ 10 | ≤ 3.4% |
| Apply a thin layer of Mupirocin to purulent areas,other areas please cover with vaseline,then apply ABD pad,then secure with paper tape BID | ≤ 10 | ≤ 3.4% |
| Urgent rad onc consult scheduled for XX/XX/XX at ***** | ≤ 10 | ≤ 3.4% |
| continue Gabapentin 400mg qAM and qafternoon,800mg qHS | ≤ 10 | ≤ 3.4% |
| Started dex 10mg IV BID | ≤ 10 | ≤ 3.4% |
| Completed dex 10mg IV BID until XX/XX/XX PM and switched to 5mg IV BID starting XX/XX/XX | ≤ 10 | ≤ 3.4% |
| Continue dex 10mg IV BID | ≤ 10 | ≤ 3.4% |
| Continue dex 10mg IV TID | ≤ 10 | ≤ 3.4% |
| Removal of PICC,Hold further AC given thrombocytopenia | ≤ 10 | ≤ 3.4% |
| CMP daily,consider renal consult if worsens | ≤ 10 | ≤ 3.4% |
| Start Solumedrol 1gram IV daily x3 days | ≤ 10 | ≤ 3.4% |
| Continue physiologic replacement: -cortef 10mg qAM - 5mg q Afternoon - 5mg qPM -stress dose: hydrocortisone 50 mg/m2 = 100 mg,followed by 12.5 mg/m2 (26mg) q6 hours x 24 hours | ≤ 10 | ≤ 3.4% |
| Encourage PO intake | ≤ 10 | ≤ 3.4% |
| Hold fluconazole,using micafungin for ppx | ≤ 10 | ≤ 3.4% |
| Stopped metop on XX/XX/XX | ≤ 10 | ≤ 3.4% |
| Increase Midodrine to 5mg PO TID | ≤ 10 | ≤ 3.4% |
| Added flomax | ≤ 10 | ≤ 3.4% |
| Hold fluconazole,using micafungin for ppx,d/c-ed tylenol on XX/XX/XX | ≤ 10 | ≤ 3.4% |
| Stop metformin | ≤ 10 | ≤ 3.4% |
| Lung expansion pathway with RT | ≤ 10 | ≤ 3.4% |
| Developed acute hypoxia on XX/XX/XX,requiring 6L NC. Hypotension on XX/XX/XX responsive to IV fluids. CT chest XX/XX/XX consistent with pulmonary edema. | ≤ 10 | ≤ 3.4% |
| Start Midodrine 2.5mg PO TID | ≤ 10 | ≤ 3.4% |
| Started on dex,received 10mg IV @ 1839 | ≤ 10 | ≤ 3.4% |
| Cont vimpat 100mg BID for seizure ppx,continue salt tabs 3g TID,further adjustments pending sodium trend; fluid restriction lifted as it was limiting his solute intake | ≤ 10 | ≤ 3.4% |
| Received 1 dose of ***** | ≤ 10 | ≤ 3.4% |
| Foley placement | ≤ 10 | ≤ 3.4% |
| Continues on ***** 10mg IV TID | ≤ 10 | ≤ 3.4% |
| Cardiology consult,Atropine at bedside,Monitor closely on telemetry,Stop offending medications,including zofran and dex,Steroids now switched from ***** to Solumedrol,Changed Fluc to caspo,daily ECG | ≤ 10 | ≤ 3.4% |
| Added flomax XX/XX/XX | ≤ 10 | ≤ 3.4% |
