# Supplementary material for: Extracting adverse event nature, severity, timelines and resulting interventions from clinical notes of patients receiving CAR-T cell therapy using large language models
Source: PLOS Digit Health. 2026 Aug 27;5(8):e0001426. doi: 10.1371/journal.pdig.0001426 (PMC13521353; doi:10.1371/journal.pdig.0001426)
Supplement: Supplementary file 8 [file pdig.0001426.s010.docx]

S7 Table Top 30 Clinical Intervention for Grade 0 CRS

| **Clinical intervention** | **Frequency (patients)** |
| --- | --- |
| Monitor for signs/symptoms of CRS/Neurotoxicity | ≤ 10 |
| Decreased to dex 10mg IV BID | ≤ 10 |
| Decreased to ***** 10mg IV BID | ≤ 10 |
| Continue ***** 10mg IV BID | ≤ 10 |
| No intervention | ≤ 10 |
| Notify heme fellow or attending for fevers,hemodynamic instability,hypoxia,or neuro changes. Heme attending to decide if tocilizumab is appropriate if c/f CRS. Infectious work up and treatment PRN for fevers (including any neutropenic fever). Monitor ICE score per protocol. | ≤ 10 |
| No new interventions | ≤ 10 |
| No signs of CRS or neurotoxicity since. | ≤ 10 |
| No steroids | ≤ 10 |
| None so far | ≤ 10 |
| Notify heme fellow or attending for fevers,hemodynamic instability,hypoxia,or neuro changes. Heme attending to decide if tocilizumab is appropriate if c/f CRS. Infectious work up and treatment PRN for fevers (including any neutropenic fever). Monitor ICE score per protocol CRS. | ≤ 10 |
| ***** 10mg IV BID | ≤ 10 |
| Now off ***** | ≤ 10 |
| Now off dex | ≤ 10 |
| Off dex | ≤ 10 |
| RN to cont BID neuro checks per study protocol | ≤ 10 |
| Received dex 10 mg IV | ≤ 10 |
| Received dex 10mg | ≤ 10 |
| Received dex 10mg IV | ≤ 10 |
| Received tocilizumab #2,dex 10mg x1 | ≤ 10 |
| Notify heme fellow or attending for fevers,hemodynamic instability,hypoxia,or neuro changes; Heme attending to decide if tocilizumab is appropriate if c/f CRS; Infectious work up and treatment PRN for fevers (including any neutropenic fever); Monitor ICE score per protocol | ≤ 10 |
| No decadron | ≤ 10 |
| Received tylenol x3 | ≤ 10 |
| Increased dexamethasone to 10mg q6H | ≤ 10 |
| Heme attending ***** ***** ***** tocilizumab is appropriate if c/f CRS | ≤ 10 |
| ICE XX/XX/XX | ≤ 10 |
| ICE pending admission | ≤ 10 |
| Increased ***** to 10mg q6H | ≤ 10 |
| Increased ***** to 10mg q6H (XX/XX/XX-XX/XX/XX) | ≤ 10 |
| Increased dex 10mg IV to q8h | ≤ 10 |
