# Supplementary material for: Extracting adverse event nature, severity, timelines and resulting interventions from clinical notes of patients receiving CAR-T cell therapy using large language models
Source: PLOS Digit Health. 2026 Aug 27;5(8):e0001426. doi: 10.1371/journal.pdig.0001426 (PMC13521353; doi:10.1371/journal.pdig.0001426)
Supplement: Supplementary file 9 [file pdig.0001426.s011.docx]

S8 Table Top 30 Clinical intervention for Grade 1 CRS

| **Clinical intervention** | **Frequency (patients)** |
| --- | --- |
| ***** x 1 | 18 |
| ***** | ≤ 10 |
| Tocilizumab | ≤ 10 |
| Received ***** x 1 | ≤ 10 |
| Tylenol | ≤ 10 |
| ***** x1 | ≤ 10 |
| s/p ***** x 1 | ≤ 10 |
| Oncologist to determine steroids vs ***** admin | ≤ 10 |
| tocilizumab | ≤ 10 |
| 2nd dose of ***** | ≤ 10 |
| Tocilizumab x 1 | ≤ 10 |
| ***** x 1 XX/XX/XX at 0309 then second dose XX/XX/XX at 1734 | ≤ 10 |
| One dose of ***** | ≤ 10 |
| Resolved with ***** | ≤ 10 |
| ***** given x 1 | ≤ 10 |
| ***** given x1 | ≤ 10 |
| Given ***** | ≤ 10 |
| tocilizumab x 1 | ≤ 10 |
| Received ***** | ≤ 10 |
| Tocilizumab administration | ≤ 10 |
| s/p ***** | ≤ 10 |
| Monitor for signs/symptoms of CRS/Neurotoxicity,RN to cont BID neuro checks per study protocol,Oncologist to determine steroids vs ***** admin | ≤ 10 |
| Monitor for signs/symptoms of CRS/Neurotoxicity | ≤ 10 |
| Received ***** x 1 on XX/XX/XX | ≤ 10 |
| Administered ***** and dex | ≤ 10 |
| ICE XX/XX/XX | ≤ 10 |
| Tylenol given | ≤ 10 |
| Tocilizumab #1 | ≤ 10 |
| Tocilizumab dose #1 | ≤ 10 |
| Tocilizumab administration on XX/XX/XX,Supportive care with Tylenol q6h PRN,Continuous pulse ox | ≤ 10 |
