# Supplementary material for: Extracting adverse event nature, severity, timelines and resulting interventions from clinical notes of patients receiving CAR-T cell therapy using large language models
Source: PLOS Digit Health. 2026 Aug 27;5(8):e0001426. doi: 10.1371/journal.pdig.0001426 (PMC13521353; doi:10.1371/journal.pdig.0001426)
Supplement: Supplementary file 10 [file pdig.0001426.s012.docx]

S9 Table Top 30 clinical intervention for Grade 1-2 CRS

| **Clinical intervention** | **Frequency (patients)** |
| --- | --- |
| 1 Tocilizumab x 1 on XX/XX/XX and dex 10 mg (XX/XX/XX-XX/XX/XX) | ≤ 10 |
| Tocilizumab x 1 and dex 10 mg (XX/XX/XX to XX/XX/XX) | ≤ 10 |
| Started dex 10 q12,plan for 6 total doses | ≤ 10 |
| Started on Anakinra 200 mg BID | ≤ 10 |
| Started on Anakinra 200 mg BID,continue dexamethasone | ≤ 10 |
| Started on Anakinra 200 mg BID,increased dex to 10mg Q6H | ≤ 10 |
| Tocilizumab administration,Dex administration | ≤ 10 |
| Tocilizumab administration,Dexamethasone administration | ≤ 10 |
| Tylenol given for persistent fevers & tachycardia,developing pain | ≤ 10 |
| 1 dose of tocilizumab,dex 10 mg Q12h,zosyn,keppra for prophy | ≤ 10 |
| Tylenol given. ***** given for persistent fevers & tachycardia,developing pain | ≤ 10 |
| Tylenol given. ***** given for persistent fevers & tachycardia,developing pain. EKG,echo,CXR done for c/o lower sternal pain | ≤ 10 |
| Tylenol,***** | ≤ 10 |
| s/p ***** | ≤ 10 |
| s/p *****,amiodarone gtt,metoprolol IV pushes | ≤ 10 |
| tocilizumab (ACTEMRA) IVPB (adult),steroids/***** per CRI,STOP abx/carbapenem,management for sepsis | ≤ 10 |
| Started dex 10 q12 | ≤ 10 |
| Spiked a fever,hypertension. Cultures,VBG,viral w/u ordered. Tylenol given. ***** given for persistent fevers & tachycardia,developing pain. EKG,echo,CXR done for c/o lower sternal pain. C/o headaches and lower back pain (respond to tylenol and dilaudid). ALC 90. | ≤ 10 |
| Spiked a fever,hypertension. Cultures,VBG,viral w/u ordered. Tylenol given | ≤ 10 |
| S/p ***** x 1 (XX/XX/XX,XX/XX/XX),dex 10mg x1 (XX/XX/XX),then dex 10mg q6H (XX/XX/XX-XX/XX/XX),10 mg q12 (XX/XX/XX-XX/XX/XX),then stopped | ≤ 10 |
| Received 2 doses of ***** and started on Decadron 10mg PO BID | ≤ 10 |
| Increased to 10mg Q6H | ≤ 10 |
| Increased dex to 10mg Q6H | ≤ 10 |
| Dexamethasone treatment | ≤ 10 |
| Decadron 10mg PO BID,***** x2 | ≤ 10 |
| Continued Anakinra 200 mg BID,increased dex to 10mg Q6H | ≤ 10 |
| Continued Anakinra 200 mg BID,continued dex | ≤ 10 |
| CTM,Page CRI for any new concerns for CRS | ≤ 10 |
| Amio load,steroids/***** per CRI,management for sepsis,IVF,Afib control: Amio gtt to PO (XX/XX/XX- ) with goal to load 4-6g (will receive 2.5g by IV in first 24hr) Metop tartrate 25 q6h [ ] back up- esmolol gtt > dilt gtt,Pressors PRN: 1st line levophed,***** for CRS--> decadron started and to continue per CRI,APAP for fevers,***** repletions | ≤ 10 |
