# Supplementary material for: Extracting adverse event nature, severity, timelines and resulting interventions from clinical notes of patients receiving CAR-T cell therapy using large language models
Source: PLOS Digit Health. 2026 Aug 27;5(8):e0001426. doi: 10.1371/journal.pdig.0001426 (PMC13521353; doi:10.1371/journal.pdig.0001426)
Supplement: Supplementary file 11 [file pdig.0001426.s013.docx]

S10 Table Top 30 Clinical Intervention for Grade 2 CRS

| **Clinical intervention** | **Frequency (patients)** |
| --- | --- |
| ***** x 1 | ≤ 10 |
| ***** | ≤ 10 |
| Received ***** x 1 | ≤ 10 |
| Tocilizumab x 1 | ≤ 10 |
| Tocilizumab administration | ≤ 10 |
| Tocilizumab and Dexamethasone administration | ≤ 10 |
| Dexamethasone 10mg IV BID (XX/XX/XX-) | ≤ 10 |
| Treated with ***** | ≤ 10 |
| ***** x2 | ≤ 10 |
| ***** x 1 on XX/XX/XX | ≤ 10 |
| ***** x1 | ≤ 10 |
| Tocilizumab | ≤ 10 |
| tocilizumab | ≤ 10 |
| Third dose of ***** administered,Restarted dexamethasone 10mg IV Q6H | ≤ 10 |
| Third dose of ***** administered on XX/XX/XX at 5:42 am | ≤ 10 |
| Oxygen and ***** x 2 | ≤ 10 |
| Sent infectious work up,started cefepime and received ***** x 1 | ≤ 10 |
| Second dose of Tocilizumab given | ≤ 10 |
| SBP <100,not responsive to fluids - given ***** x1 10am dose #1 - CRS G2 - ICE 10,no neurotox. 2100 - SBP <100,no further fevers,ordered ***** dose #2 - discussed with RN - discussed with night float - message heme fellow on call Monitor VS closely through the night. Consider Dose #3 around 0500 (8hrs from last dose) based upon VS. | ≤ 10 |
| S/p ***** x1 on XX/XX/XX @0730,s/p ***** x1 on XX/XX/XX@***** | ≤ 10 |
| S/p ***** x1 on XX/XX/XX @0730 | ≤ 10 |
| S/p ***** x 1 dose and dex 10 mg IV Q6H on XX/XX/XX. ***** deescalated to Q8H on XX/XX/XX. ***** deescalated to Q12H on XX/XX/XX. Received addition dose on XX/XX/XX. Last day of dex on XX/XX/XX. | ≤ 10 |
| S/p ***** on XX/XX/XX,dex 10mg x1 on XX/XX/XX,XX/XX/XX | ≤ 10 |
| Oxygen and ***** x 2 (XX/XX/XX & XX/XX/XX),Midodrine to 5mg PO TID (XX/XX/XX- ,last incr XX/XX/XX- ),Keppra 500mg BID through Day 30 (can stop after XX/XX/XX) | ≤ 10 |
| S/p ***** on XX/XX/XX,S/p ***** on XX/XX/XX,S/p dex x4 doses for CRS/neurotox | ≤ 10 |
| S/p ***** on XX/XX/XX,S/p ***** on XX/XX/XX,Monitor for symptoms of CRS/neurotoxicity,Keppra 500mg BID ppx | ≤ 10 |
| S/p ***** on XX/XX/XX and again on XX/XX/XX - Weaned off oxygen XX/XX/XX. | ≤ 10 |
| S/p ***** on XX/XX/XX and XX/XX/XX,dex 10mg x1 on XX/XX/XX,XX/XX/XX | ≤ 10 |
| Restarted dexamethasone 10mg IV Q6H | ≤ 10 |
| Restarted Dexamethasone 10mg IV BID on XX/XX/XX and now with somewhat improving mental status,and concern for underlying CRS/neurotoxicity. Decrease Dexamethasone to 10mg IV q12 (XX/XX/XX--> ) (previously on XX/XX/XX 10 BID,XX/XX/XX 10q6,XX/XX/XX 10 TID) | ≤ 10 |
