# Supplementary material for: Extracting adverse event nature, severity, timelines and resulting interventions from clinical notes of patients receiving CAR-T cell therapy using large language models
Source: PLOS Digit Health. 2026 Aug 27;5(8):e0001426. doi: 10.1371/journal.pdig.0001426 (PMC13521353; doi:10.1371/journal.pdig.0001426)
Supplement: Supplementary file 12 [file pdig.0001426.s014.docx]

S11 Table All Clinical Intervention for Grade 2-3 CRS

| **Clinical intervention** | **Frequency (patients)** |
| --- | --- |
| 1 mg/kg solumedrol q12h | ≤ 10 |
| Cultured,drew labs,started cefepime,gave a dose of tylenol | ≤ 10 |
| dexamethasone 10 mg IV Q6H and ICU transfer | ≤ 10 |
| currently on 1 mg/kg solumedrol q12h. With new onset facial tingling,MRI brain w/wo and ***** demonstrated multiple osseous lesions at calvarium and skull base with increased enhancement. Started solumedrol wean to 0.5mg/kg q12h on XX/XX/XX ***** if continues with clinical stability. | ≤ 10 |
| cefepime,tylenol,tocilizumab 800mg,PICU transfer | ≤ 10 |
| cefepime,tylenol,NS 1L bolus,***** 800mg | ≤ 10 |
| Tylenol,***** x1,NS bolus,albumin,methylpred started. Third spacing. Seen by PICU/RRT team but supportive care measures had good responses and vitals stabilized. Lasix 10mg given once vitals stabilize to diurese some fluid | ≤ 10 |
| Tylenol,***** x1,NS bolus,albumin,methylpred started. Lasix 10mg given once vitals stabilize to diurese some fluid | ≤ 10 |
| Tylenol,***** x1,NS bolus,albumin,methylpred started | ≤ 10 |
| Tylenol,***** x1,NS bolus,albumin,methylpred | ≤ 10 |
| On 1 mg/kg solumedrol q12h. Started solumedrol wean to 0.5mg/kg q12h on XX/XX/XX | ≤ 10 |
| On 1 mg/kg solumedrol q12h,started solumedrol wean to 0.5mg/kg q12h on XX/XX/XX | ≤ 10 |
| On 1 mg/kg solumedrol q12h | ≤ 10 |
| NS 1L bolus given followed by a dose of ***** 800mg and PICU made aware | ≤ 10 |
| Febrile,tachycardia,hypotensive,hypoxic. Tylenol,***** x1,NS bolus,albumin,methylpred started. Third spacing. Seen by PICU/RRT team but supportive care measures had good responses and vitals stabilized. Lasix 10mg given once vitals stabilize to diurese some fluid. Pain other than intermittent headaches resolved,using dilaudid prn. ALC 160. Discussed with Dr. *****. ***** dose repeated at 2230 for fevers and hypotension. | ≤ 10 |
| Febrile,tachycardia,hypotensive,hypoxic. Tylenol,***** x1,NS bolus,albumin,methylpred started | ≤ 10 |
| Dexamethasone 10 mg IV Q6H and ICU transfer | ≤ 10 |
| Dexamethasone 10 mg IV Q6H | ≤ 10 |
| Currently on 1 mg/kg solumedrol q12h. With new onset facial tingling,MRI brain w/wo and ***** demonstrated multiple osseous lesions at calvarium and skull base with increased enhancement. Started solumedrol wean to 0.5mg/kg q12h on XX/XX/XX ***** if continues with clinical stability. | ≤ 10 |
| Cultured,drew labs,started cefepime,gave a dose of tylenol. Transferred to PICU following 2nd bolus when BP dropped to 70s/40s | ≤ 10 |
| Cultured,drew labs,started cefepime,gave a dose of tylenol. Discussed with Dr. *****,IT attending. Pt was persistently and increasingly febrile and tachycardic with decreased BP XX/XX/XX and 1 episode of hypoxemia 95% at approx. 14:30 -> NS 1L bolus given followed by a dose of ***** 800mg and PICU made aware. Transferred to PICU following 2nd bolus when BP dropped to 70s/40s as well as previously receiving prbc. No pressors required overnight,***** not needing redose. | ≤ 10 |
| Cultured,drew labs,started cefepime,gave a dose of tylenol. Discussed with Dr. *****,IT attending. Pt was persistently and increasingly febrile and tachycardic with decreased BP XX/XX/XX and 1 episode of hypoxemia 95% at approx. 14:30 -> NS 1L bolus given followed by a dose of ***** 800mg and PICU made aware. | ≤ 10 |
| Cultured,drew labs,started cefepime,gave a dose of tylenol,NS 1L bolus given followed by a dose of ***** 800mg and PICU made aware | ≤ 10 |
| methylprednisolone | ≤ 10 |
