# Supplementary material for: Extracting adverse event nature, severity, timelines and resulting interventions from clinical notes of patients receiving CAR-T cell therapy using large language models
Source: PLOS Digit Health. 2026 Aug 27;5(8):e0001426. doi: 10.1371/journal.pdig.0001426 (PMC13521353; doi:10.1371/journal.pdig.0001426)
Supplement: Supplementary file 13 [file pdig.0001426.s015.docx]

S12 Table All Clinical Intervention for Grade 3 CRS

| **Clinical intervention** | **Frequency (patients)** |
| --- | --- |
| 1 dose of ***** on XX/XX/XX,Started on ***** 10mg IV Q6h on XX/XX/XX received a total of 3 doses then increased to 20mg IV Q6h x4 doses on XX/XX/XX | ≤ 10 |
| Administer dexamethasone 10 mg intravenously every 6 hours. Continue dexamethasone use until the event is Grade 1 or less,then taper over 3 days. | ≤ 10 |
| methylpred 1g qday followed by taper XX/XX/XX - XX/XX/XX | ≤ 10 |
| dex 10mg IV q6 hours | ≤ 10 |
| Transferred to the PICU for close neuro and hemodynamic monitoring. S/p tocilizumab x1 and started methylprednisolone on XX/XX/XX with improvement in symptoms and did not require vasopressin. | ≤ 10 |
| Tocilizumab | ≤ 10 |
| Started on Decadron 20mg IV q6h,Stat NCHCT,Start EEG monitoring,Increase Keppra to 1,000mg IV BID,Blood and urine culture,Neurology consult | ≤ 10 |
| Started on Decadron 20mg IV q6h stat after discussion with attending. See other neuro eval/interventions below. Approximately 1 hour after first dose of steroids administered (~1100) pt with dramatic improvement,ICE score 10,remains disoriented but now forming sentences. Throughout the day patient with ongoing intermittent,aphasia,but overall improving encephalopathy. | ≤ 10 |
| Started on Decadron 20mg IV q6h | ≤ 10 |
| Started on ***** 10mg IV Q6h on XX/XX/XX received a total of 3 doses then increased to 20mg IV Q6h doses on XX/XX/XX. Solumedrol 1000mg x1 given XX/XX/XX. | ≤ 10 |
| Started on ***** 10mg IV Q6h on XX/XX/XX received a total of 3 doses then increased to 20mg IV Q6h doses on XX/XX/XX. Solumedrol 1000mg x1 given XX/XX/XX,XX/XX/XX. Ordered to receive again on XX/XX/XX. Continue Anakinra 100mg SC daily (XX/XX/XX-) | ≤ 10 |
| Started on ***** 10mg IV Q6h on XX/XX/XX received a total of 3 doses then increased to 20mg IV Q6h doses on XX/XX/XX | ≤ 10 |
| Start Dexamethasone 20mg IV Q6H | ≤ 10 |
| Received both ***** and Methylprednisolone | ≤ 10 |
| Received Tocilizumab x3 (XX/XX/XX,XX/XX/XX,XX/XX/XX) and started on Dexamethasone 10 mg IV BID for 3 days (XX/XX/XX-XX/XX/XX) | ≤ 10 |
| Dexamethasone 10mg IV q6h,Tocilizumab 8mg/kg IV | ≤ 10 |
| Continued dexamethasone 10mg IV q6h,tocilizumab administered on XX/XX/XX | ≤ 10 |
| Continue decadron 10mg q6h | ≤ 10 |
| Administer methylprednisolone 1 mg/kg intravenously twice daily or equivalent dexamethasone (e.g.,10 mg intravenously every 6 hours). Continue corticosteroids use until the event is Grade 1 or less,then taper over 3 days. | ≤ 10 |
| s/p ***** 10mg IV x 1 and x3 on XX/XX/XX | ≤ 10 |
