# Supplementary material for: Extracting adverse event nature, severity, timelines and resulting interventions from clinical notes of patients receiving CAR-T cell therapy using large language models
Source: PLOS Digit Health. 2026 Aug 27;5(8):e0001426. doi: 10.1371/journal.pdig.0001426 (PMC13521353; doi:10.1371/journal.pdig.0001426)
Supplement: Supplementary file 14 [file pdig.0001426.s016.docx]

S13 Table All Clinical Intervention for CRS Grade 4

| **Clinical intervention** | **Frequency (patients)** |
| --- | --- |
| From dex to methylprednisone pulse. He improved starting XX/XX/XX and nearly cleared by XX/XX/XX but recurred on XX/XX/XX and treated with additional steroids (dex),tapered off by XX/XX/XX | ≤ 10 |
| Patient is at high risk for CRS. Developed acute hypoxia on XX/XX/XX,requiring 6L NC. Hypotension on XX/XX/XX responsive to IV fluids. CT chest XX/XX/XX consistent with pulmonary edema. - S/p ***** on XX/XX/XX and again on XX/XX/XX - Weaned off oxygen XX/XX/XX. - please contact the CRI fellow (*****-*****) if patient is febrile,hypotensive,tachycardic,hypoxic or has changes in ***** or neurotoxicity signs - Ordered CT Chest w/o contrast on XX/XX/XX given persistent oxygen requirement - IV Lasix 40 mg x1 ordered on XX/XX/XX given patient appears hypervolemic with abdominal distention,facial plethora. -PO lasix 40mg BID for persistent LE edema. | ≤ 10 |
| S/p ***** on XX/XX/XX and again on XX/XX/XX,***** oxygen prn,CPO monitoring,Ordered CT Chest w/o contrast on XX/XX/XX given persistent oxygen requirement,IV Lasix 40 mg x1 ordered on XX/XX/XX given patient appears hypervolemic with abdominal distention,facial plethora | ≤ 10 |
