# Supplementary material for: Extracting adverse event nature, severity, timelines and resulting interventions from clinical notes of patients receiving CAR-T cell therapy using large language models
Source: PLOS Digit Health. 2026 Aug 27;5(8):e0001426. doi: 10.1371/journal.pdig.0001426 (PMC13521353; doi:10.1371/journal.pdig.0001426)
Supplement: Supplementary file 15 [file pdig.0001426.s017.docx]

S14 Table Top 30 Clinical Intervention for Grade 0 ICANS

| **Clinical intervention** | **Frequency (patients)** |
| --- | --- |
| ***** 10mg daily (XX/XX/XX - ) | ≤ 10 |
| Now off ***** | ≤ 10 |
| Stopping steroids after morning dose XX/XX/XX | ≤ 10 |
| Stopped steroids,received final dose this morning | ≤ 10 |
| Stopped dex | ≤ 10 |
| Started on dexamethasone 10mg IV q12h | ≤ 10 |
| Start midodrine 2.5mg TID | ≤ 10 |
| S/p Solumedrol 1gram IV daily x3 days (XX/XX/XX-XX/XX/XX) given highest c/f neurotoxicity,now on taper to 500mg IV on XX/XX/XX,and 250mg IV on XX/XX/XX,then off - Decadron 10mg q12h (XX/XX/XX - ) - S/p decadron 10mg q12h (XX/XX/XX - XX/XX/XX),s/p dex 10mg q6h (XX/XX/XX - XX/XX/XX) - Appreciate Neurology consultation - S/p Keppra load with 3grams IV x1 dose on XX/XX/XX,and increase standing Keppra to 1gram BID - OK to deescalate abx and defer LP - TTE,tele for stroke w/u negative - Delirium precautions - PT/OT/ST eval. PT eval'd XX/XX/XX w/ rec for home with PT | ≤ 10 |
| Remains off ***** | ≤ 10 |
| Remained off dex | ≤ 10 |
| Received ***** x1 at 1640 | ≤ 10 |
| RN neuro checks q shift | ≤ 10 |
| Now off dex | ≤ 10 |
| Notify Immunotherapy Attending | ≤ 10 |
| Taper ***** 10mg IV q12h | ≤ 10 |
| None so far | ≤ 10 |
| No steroids | ≤ 10 |
| No new interventions | ≤ 10 |
| Neurology consulted for new myoclonic activity EEG XX/XX/XX. Consider imaging if symptoms progress or EEG is abnormal. Continue neuro checks Continue Keppra for seizure ppx,start with chemo and continue until day +28 | ≤ 10 |
| Monitoring for signs/symptoms of CRS/Neurotoxicity,Levetiracetam 500mg BID for seizure prophylaxis | ≤ 10 |
| Monitoring for early neurologic signs,RN aware and monitoring closely | ≤ 10 |
| Monitor for signs/symptoms of CRS/Neurotoxicity,RN to cont BID neuro checks per study protocol,Cont Keppra,Oncologist to determine steroids vs ***** admin | ≤ 10 |
| Monitor for signs/symptoms of CRS/Neurotoxicity | ≤ 10 |
| Levetiracetam for seizure ppx,through Day 30 | ≤ 10 |
| Keppra started on first day of ***** chemotherapy and will continue through Day 21 post ***** infusion. | ≤ 10 |
| Keppra for seizure ppx,start Day 0 with ***** chemo and continue until day +28 | ≤ 10 |
| Supportive care and monitor neurologic status | ≤ 10 |
| Taper ***** 20mg IV q12h | ≤ 10 |
| Keppra for seizure ppx | ≤ 10 |
| dex 10mg x1 | ≤ 10 |
