# Supplementary material for: Extracting adverse event nature, severity, timelines and resulting interventions from clinical notes of patients receiving CAR-T cell therapy using large language models
Source: PLOS Digit Health. 2026 Aug 27;5(8):e0001426. doi: 10.1371/journal.pdig.0001426 (PMC13521353; doi:10.1371/journal.pdig.0001426)
Supplement: Supplementary file 16 [file pdig.0001426.s018.docx]

S15 Table Top 30 Clinical Intervention for Grade 1 ICANS

| **Clinical intervention** | **Frequency (patients)** |
| --- | --- |
| Started dex 10mg IV BID | ≤ 10 |
| ***** x3 and dex | ≤ 10 |
| Received ***** x1 at 1640 | ≤ 10 |
| Started dex 10 mg bid for a planned three-course | ≤ 10 |
| Solumedrol 1gram IV daily x3 days (XX/XX/XX-XX/XX/XX),now on taper to 500mg IV on XX/XX/XX,and 250mg IV on XX/XX/XX,then off | ≤ 10 |
| S/p Solumedrol 1gram IV daily x3 days (XX/XX/XX-XX/XX/XX) given highest c/f neurotoxicity,now on taper to 500mg IV on XX/XX/XX,and 250mg IV on XX/XX/XX,then off - Decadron 10mg q12h (XX/XX/XX - ) - S/p decadron 10mg q12h (XX/XX/XX - XX/XX/XX),s/p dex 10mg q6h (XX/XX/XX - XX/XX/XX) - Appreciate Neurology consultation - S/p Keppra load with 3grams IV x1 dose on XX/XX/XX,and increase standing Keppra to 1gram BID - OK to deescalate abx and defer LP - TTE,tele for stroke w/u negative - Delirium precautions - PT/OT/ST eval. PT eval'd XX/XX/XX w/ rec for home with PT | ≤ 10 |
| S/p Solumedrol 1gram IV daily x3 days (XX/XX/XX-XX/XX/XX) given highest c/f neurotoxicity,now on taper to 500mg IV on XX/XX/XX,and 250mg IV on XX/XX/XX,then off - Decadron 10mg BID (XX/XX/XX - ) - Appreciate Neurology consultation - S/p Keppra load with 3grams IV x1 dose on XX/XX/XX,and increase standing Keppra to 1gram BID - OK to deescalate abx and defer LP - TTE,tele for stroke w/u negative - Delirium precautions - PT/OT/ST eval,need ARU placement and will depend on neurotoxicity status | ≤ 10 |
| S/p Solumedrol 1gram IV daily x3 days (XX/XX/XX-XX/XX/XX) given highest c/f neurotoxicity,now on taper to 500mg IV on XX/XX/XX,and 250mg IV on XX/XX/XX,then off | ≤ 10 |
| S/p Keppra load with 3grams IV x1 dose on XX/XX/XX,and increase standing Keppra to 1gram BID | ≤ 10 |
| Resumed on decadron 10mg BID (XX/XX/XX - XX/XX/XX). | ≤ 10 |
| Received dex 10mg IV | ≤ 10 |
| Received dex 10mg | ≤ 10 |
| Received Tocilizumab x3 (XX/XX/XX,XX/XX/XX,XX/XX/XX) and started on Dexamethasone 10 mg IV BID for 3 days (XX/XX/XX-XX/XX/XX) | ≤ 10 |
| ***** 10mg IV x 1 given | ≤ 10 |
| Started dex 10mg IV BID,increased to 10mg x3 on XX/XX/XX,plan to taper to 10mg IV BID,then to 10mg IV daily,then to 4mg PO daily,then off | ≤ 10 |
| No new interventions | ≤ 10 |
| Methylpred 1mg/kg on XX/XX/XX | ≤ 10 |
| Managed as specified in overview | ≤ 10 |
| Keppra for seizure prophylaxis,started with ***** chemo and continued until day +21 (stopped XX/XX/XX) | ≤ 10 |
| Keppra for seizure prophylaxis | ≤ 10 |
| Increased to dex 10mg IV increased to q8h | ≤ 10 |
| Increased dex 10mg IV increased to q8h | ≤ 10 |
| Increased decadron 10mg q6h (XX/XX/XX - ) | ≤ 10 |
| Improving on ***** | ≤ 10 |
| None so far | ≤ 10 |
| Started dex 10mg IV q6h (for borderline G2 ICANs without concurrent CRS) | ≤ 10 |
| IV Dexamethasone 10mg q6h | ≤ 10 |
| Started dex,received 10mg IV | ≤ 10 |
| tapering dex,now 20mg IV q8h | ≤ 10 |
| s/p solumedrol 1g IV daily x 3 doses (XX/XX/XX-XX/XX/XX) then 250mg IV x 1 on XX/XX/XX | ≤ 10 |
