# Supplementary material for: Extracting adverse event nature, severity, timelines and resulting interventions from clinical notes of patients receiving CAR-T cell therapy using large language models
Source: PLOS Digit Health. 2026 Aug 27;5(8):e0001426. doi: 10.1371/journal.pdig.0001426 (PMC13521353; doi:10.1371/journal.pdig.0001426)
Supplement: Supplementary file 17 [file pdig.0001426.s019.docx]

S16 Table All Clinical Intervention for Grade 1-2 ICANS

| **Clinical intervention** | **Frequency (patients)** |
| --- | --- |
| 1 dose tocilizumab on XX/XX/XX and dex 10 mg (XX/XX/XX-XX/XX/XX),continue keppra 500 mg PO BID through day +30 | ≤ 10 |
| Steroids/***** per CRI,STOP abx/carbapenem,management for sepsis | ≤ 10 |
| s/p amio load,steroids/***** per CRI,s/p abx,management for sepsis,IVF,Afib control: s/p amio load,dc amio titrate metop to 50mg q8h,*****/steroids for CRS--> decadron per CRI,APAP for fevers,***** repletions,s/p vanc/mero (XX/XX/XX),Zosyn (XX/XX/XX) | ≤ 10 |
