# Supplementary material for: Extracting adverse event nature, severity, timelines and resulting interventions from clinical notes of patients receiving CAR-T cell therapy using large language models
Source: PLOS Digit Health. 2026 Aug 27;5(8):e0001426. doi: 10.1371/journal.pdig.0001426 (PMC13521353; doi:10.1371/journal.pdig.0001426)
Supplement: Supplementary file 18 [file pdig.0001426.s020.docx]

S17 Table Top 30 Clinical Intervention for Grade 2 ICANS

| **Clinical intervention** | **Frequency (patients)** |
| --- | --- |
| ***** | ≤ 10 |
| Received tocilizumab x 2 on XX/XX/XX and XX/XX/XX and dexamethasone 10 mg IV x 2 on XX/XX/XX. From XX/XX/XX-XX/XX/XX,his encephalopathy worsened and he was started on dexamethasone 10 mg IV Q6H,w/ subsequent significant improvement. | ≤ 10 |
| Tocilizumab 8 mg/kg (760 mg) x 1 on XX/XX/XX and XX/XX/XX - ***** 10 mg x 1 on XX/XX/XX then 10 mg Q6 (XX/XX/XX-XX/XX/XX) > 10 mg Q12 (XX/XX/XX-XX/XX/XX) > 10 mg/day (XX/XX/XX-XX/XX/XX) > sx resolved prior to discharge | ≤ 10 |
| Steroids treatment from XX/XX/XX to XX/XX/XX | ≤ 10 |
| Steroids XX/XX/XX-XX/XX/XX | ≤ 10 |
| Steroid treatment,Monitoring for symptoms,Thiamine administration,CT-guided LP | ≤ 10 |
| Started on steroids (dex 10mg IV BID),dex 10 mg q6h x 4 doses,dex 10 mg q8h x 3 doses,dex 10 mg q12h x 2 doses | ≤ 10 |
| Started on steroids (dex 10mg IV BID),Neuro checks,Taper plan: dex 10 mg q8h x 3 doses XX/XX/XX - dex 10 mg q12h x 2 doses XX/XX/XX - off dex after this,monitor for 24H for symptoms | ≤ 10 |
| Started on dexamethasone 10 mg IV Q6H | ≤ 10 |
| Started on dex 10 mg IV | ≤ 10 |
| Started on dex | ≤ 10 |
| Started on ***** 10mg IV Q6h on XX/XX/XX received a total of 3 doses then increased to 20mg IV Q6h x4 doses on XX/XX/XX | ≤ 10 |
| Started on ***** 10mg IV Q6h on XX/XX/XX received a total of 3 doses then increased to 20mg IV Q6h doses on XX/XX/XX. Solumedrol 1000mg x1 given XX/XX/XX,XX/XX/XX. Ordered to receive again on XX/XX/XX. Continue Anakinra 100mg SC daily (XX/XX/XX-) - ***** switched to Solumedrol 100mg IV q6hours on XX/XX/XX given concern that dex is contributing to severe bradycardia. | ≤ 10 |
| Started on ***** 10mg IV Q6h | ≤ 10 |
| Started dexamethasone x 4 doses,continued dex 10mg iv Q6hr (XX/XX/XX) | ≤ 10 |
| Started dexamethasone XX/XX/XX 10 mg IV q6 hrs | ≤ 10 |
| Started dex 10 q 12 x 6 doses | ≤ 10 |
| Started IV Dexamethasone 10 mg twice daily x 3 days | ≤ 10 |
| S/p Solumedrol 1gram IV daily x3 days (XX/XX/XX-XX/XX/XX) given highest c/f neurotoxicity,now on taper to 500mg IV on XX/XX/XX,and 250mg IV on XX/XX/XX,then off - Decadron 10mg q12h (XX/XX/XX - ) - S/p decadron 10mg q12h (XX/XX/XX - XX/XX/XX),s/p dex 10mg q6h (XX/XX/XX - XX/XX/XX) - Appreciate Neurology consultation - S/p Keppra load with 3grams IV x1 dose on XX/XX/XX,and increase standing Keppra to 1gram BID - OK to deescalate abx and defer LP - TTE,tele for stroke w/u negative - Delirium precautions - PT/OT/ST eval. PT eval'd XX/XX/XX w/ rec for home with PT | ≤ 10 |
| S/p ***** 10mg q6h (XX/XX/XX to XX/XX/XX,first dose at 0930 on XX/XX/XX),***** 10mg q12h (XX/XX/XX - 23),***** 10mg daily (XX/XX/XX - XX/XX/XX),Delirium precautions: open curtains during the day,***** ***** *****,minimize noise at night. | ≤ 10 |
| S/p ***** 10mg q6h (XX/XX/XX to XX/XX/XX,first dose at 0930 on XX/XX/XX),***** 10mg q12h (XX/XX/XX - 23),***** 10mg daily (XX/XX/XX - ),MRI Brain w wo contrast ordered XX/XX/XX (pending),EEG,Delirium precautions: open curtains during the day,***** ***** *****,minimize noise at night,Appreciate neuro consult | ≤ 10 |
| S/p ***** 10mg q6h (XX/XX/XX to XX/XX/XX,first dose at 0930 on XX/XX/XX) | ≤ 10 |
| Restarted dexamethasone 10mg IV BID | ≤ 10 |
| Restarted Dexamethasone 10mg IV BID,Neuro consultation,NCHCT and video EEG planned | ≤ 10 |
| Restarted Dexamethasone 10mg IV BID on XX/XX/XX and now with somewhat improving mental status,and concern for underlying CRS/neurotoxicity. Decrease Dexamethasone to 10mg IV q12 (XX/XX/XX--> ) (previously on XX/XX/XX 10 BID,XX/XX/XX 10q6,XX/XX/XX 10 TID). Keppra 500mg IV q12h (restart XX/XX/XX-) -- please keep on until at least day 30 (XX/XX/XX) | ≤ 10 |
| Restarted ***** 10mg daily x 3 days | ≤ 10 |
| Restarted ***** 10mg IV q8h (XX/XX/XX-,incr XX/XX/XX-,decr XX/XX/XX-),Restart keppra 500mg IV q12h (restart XX/XX/XX-) | ≤ 10 |
| Restarted ***** 10mg IV q6h (XX/XX/XX-,incr XX/XX/XX-),Restart keppra 500mg IV q12h (restart XX/XX/XX-) | ≤ 10 |
| Restarted ***** 10mg IV BID on XX/XX/XX,completed ***** taper on XX/XX/XX | ≤ 10 |
| Restarted ***** 10mg IV BID on XX/XX/XX and now with somewhat improving mental status,and concern for underlying CRS/neurotoxicity. Management: - Appreciate Neurology recs,now signed off - Completed ***** ***** on XX/XX/XX - Previously on 10q12 (XX/XX/XX-->XX/XX/XX ) XX/XX/XX 10 BID,XX/XX/XX 10q6,TID XX/XX/XX-XX/XX/XX then q12h XX/XX/XX-XX/XX/XX,10mg every day XX/XX/XX,then 4mg daily on XX/XX/XX then off) - Keppra 500mg IV q12h (restart XX/XX/XX-) -- please keep on until at least day 30 (XX/XX/XX) - See previous notes for ICE score history | ≤ 10 |
