# Supplementary material for: Extracting adverse event nature, severity, timelines and resulting interventions from clinical notes of patients receiving CAR-T cell therapy using large language models
Source: PLOS Digit Health. 2026 Aug 27;5(8):e0001426. doi: 10.1371/journal.pdig.0001426 (PMC13521353; doi:10.1371/journal.pdig.0001426)
Supplement: Supplementary file 19 [file pdig.0001426.s021.docx]

S18 Table All Clinical Intervention for Grade 2-3 ICANS

| **Clinical intervention** | **Frequency (patients)** |
| --- | --- |
| Dexamethasone 10 mg IV Q6H and ICU transfer | ≤ 10 |
| Dexamethasone 10 mg IV Q6H and ICU transfer for closer monitoring | ≤ 10 |
| Started on dexamethasone 10 mg IV Q6H for grade XX/XX/XX neurotoxicity,per the ***** FDA insert (at *****://*****.*****.*****/*****/*****.*****),Transferred to ICU for close monitoring | ≤ 10 |
| dexamethasone 10 mg IV Q6H and ICU transfer | ≤ 10 |
| dexamethasone 10 mg IV Q6H and transferred to the ICU | ≤ 10 |
