# Supplementary material for: Extracting adverse event nature, severity, timelines and resulting interventions from clinical notes of patients receiving CAR-T cell therapy using large language models
Source: PLOS Digit Health. 2026 Aug 27;5(8):e0001426. doi: 10.1371/journal.pdig.0001426 (PMC13521353; doi:10.1371/journal.pdig.0001426)
Supplement: Supplementary file 20 [file pdig.0001426.s022.docx]

S19 Table Top 30 Clinical Intervention for Grade 3 ICANS

| **Clinical intervention** | **Frequency (patients)** |
| --- | --- |
| 10mg decadron q6h | ≤ 10 |
| Started on ***** 20mg IV q6hours without improvement in symptoms. Neurology consulted,and felt EEG could be c/w seizures causing *****'s paralysis. He was loaded with Keppra. Steroid dosing increased to Solumedrol 1gram IV daily x3 days. | ≤ 10 |
| Solumedrol 1gram IV daily x3 days (XX/XX/XX-XX/XX/XX),now on taper to 500mg IV on XX/XX/XX,and 250mg IV on XX/XX/XX,then off | ≤ 10 |
| Start Dexamethasone 20mg IV Q6H | ≤ 10 |
| Start Solumedrol 1gram IV daily x3 days,Neurology consultation,ICU eval,transfer to ICU for closer monitoring and nursing care,Stat CTA brain and neck,Plan for MRI brain w/w/o contrast,Given Keppra load with 3grams IV x1 dose,and increase standing Keppra to 1gram BID,Start Meningitic dosing of abx with Acyclovir,Ceftriaxone,Vanc,Ampicillin,Plan for diagnostic LP in the AM after plts | ≤ 10 |
| Started on ***** 10mg IV Q6h on XX/XX/XX received a total of 3 doses then increased to 20mg IV Q6h doses on XX/XX/XX-> switched to Solumedrol 1000mg due to severe bradycardia XX/XX/XX dex (XX/XX/XX,XX/XX/XX,XX/XX/XX) - currently on Solumedrol taper over the next XX/XX/XX days decreasing the dose buy 50% every XX/XX/XX days. Started Taper on XX/XX/XX. Solumedrol 500mg IV daily x 2 days (XX/XX/XX-XX/XX/XX)-> solumedrol 125mg x1 on XX/XX/XX. - Continue Anakinra 100mg SC daily (XX/XX/XX-) | ≤ 10 |
| Started on ***** 10mg IV Q6h on XX/XX/XX received a total of 3 doses then increased to 20mg IV Q6h doses on XX/XX/XX. | ≤ 10 |
| Started on ***** 10mg IV Q6h on XX/XX/XX received a total of 3 doses then increased to 20mg IV Q6h doses on XX/XX/XX. - ***** switched to Solumedrol 100mg IV q6hours on XX/XX/XX given concern that dex is contributing to severe bradycardia. | ≤ 10 |
| Started on ***** 10mg IV Q6h on XX/XX/XX received a total of 3 doses then increased to 20mg IV Q6h doses on XX/XX/XX. Solumedrol 1000mg x1 given XX/XX/XX,XX/XX/XX. Ordered to receive again on XX/XX/XX. Continue Anakinra 100mg SC daily (XX/XX/XX-) - ***** switched to Solumedrol 100mg IV q6hours on XX/XX/XX given concern that dex is contributing to severe bradycardia. | ≤ 10 |
| Started on ***** 10mg IV Q6h on XX/XX/XX received a total of 3 doses then increased to 20mg IV Q6h doses on XX/XX/XX. Solumedrol 1000mg x1 given XX/XX/XX,XX/XX/XX. Ordered to receive again on XX/XX/XX. Now mental status has improved and is currently on Solumedrol taper over the next XX/XX/XX days decreasing the dose ***** 50% every XX/XX/XX days. Started Taper on XX/XX/XX. Solumedrol 500mg IV daily x 2 days (XX/XX/XX-XX/XX/XX) f/b 100mg IV daily etc. - Continue Anakinra 100mg SC daily (XX/XX/XX-) - ***** switched to Solumedrol 100mg IV q6hours on XX/XX/XX given concern | ≤ 10 |
| Started on ***** 10mg IV Q6h on XX/XX/XX received a total of 3 doses then increased to 20mg IV Q6h doses on XX/XX/XX. Solumedrol 1000mg x1 given XX/XX/XX. Plan to give Anakinra on XX/XX/XX - ***** switched to Solumedrol 100mg IV q6hours on XX/XX/XX given concern that dex is contributing to severe bradycardia. | ≤ 10 |
| Started on ***** 10mg IV Q6h on XX/XX/XX received a total of 3 doses then increased to 20mg IV Q6h x4 doses on XX/XX/XX | ≤ 10 |
| Started on ***** 20mg IV q6hours without improvement in symptoms. Neurology consulted,and felt EEG could be c/w seizures causing *****'s paralysis. | ≤ 10 |
| Started on Decadron 20mg IV q6h,Decrease Keppra back to 500mg PO BID,Follow up final blood cultures,Follow up MRI brain | ≤ 10 |
| Appreciate Neurology consultation | ≤ 10 |
| Started on Decadron 20mg IV q6h,ordered stat NCHCT,EEG,increased Keppra to 1g BID,and placed neurology consult for further eval | ≤ 10 |
| Treated with solumedrol pulse | ≤ 10 |
| Treated with solumedrol pulse,continued keppra | ≤ 10 |
| dex 10mg IV q6 hours (March - ),keppra BID D-1 through ***** | ≤ 10 |
| dexamethasone 20mg IV q6h (XX/XX/XX-XX/XX/XX) | ≤ 10 |
| methylpred 1g qday followed by taper XX/XX/XX - XX/XX/XX - MRI,EEG; if results inconclusive and not improving w/ steroids will consider LP - continue keppra BID - feeding tube placed XX/XX/XX for obtundation and nutritional support | ≤ 10 |
| s/p ***** 10mg IV x 1 on XX/XX/XX,and x3 on XX/XX/XX | ≤ 10 |
| s/p ***** x1 on XX/XX/XX ~1740,XX/XX/XX at 1530 | ≤ 10 |
| s/p solumedrol 1g IV daily x 3 doses (XX/XX/XX-XX/XX/XX) | ≤ 10 |
| s/p solumedrol 1g IV daily x 3 doses (XX/XX/XX-XX/XX/XX) then 250mg IV x 1 on XX/XX/XX | ≤ 10 |
| solumedrol 1g IV daily x 3 doses (XX/XX/XX-XX/XX/XX) then 250mg IV x 1 on XX/XX/XX | ≤ 10 |
| S/p Solumedrol 1gram IV daily x3 days (XX/XX/XX-XX/XX/XX) given highest c/f neurotoxicity,now on taper to 500mg IV on XX/XX/XX,and 250mg IV on XX/XX/XX,then off | ≤ 10 |
| S/p Keppra load with 3grams IV x1 dose on XX/XX/XX,and increase standing Keppra to 1gram BID | ≤ 10 |
| S/p ***** on XX/XX/XX and XX/XX/XX - S/p dex 10mg q6H (XX/XX/XX-XX/XX/XX) - S/p dex 10mg q12H (XX/XX/XX-XX/XX/XX) | ≤ 10 |
| Plan to bolus PRN for any soft pressures. | ≤ 10 |
