# Supplementary material for: Extracting adverse event nature, severity, timelines and resulting interventions from clinical notes of patients receiving CAR-T cell therapy using large language models
Source: PLOS Digit Health. 2026 Aug 27;5(8):e0001426. doi: 10.1371/journal.pdig.0001426 (PMC13521353; doi:10.1371/journal.pdig.0001426)
Supplement: Supplementary file 21 [file pdig.0001426.s023.docx]

S20 Table All Clinical Intervention for Grade 3-4 ICANS

| **Clinical intervention** | **Frequency (patients)** |
| --- | --- |
| LP today w/ TAPS,f/u gram stain,culture,cell count/diff,cytology,glucose,protein; continue keppra 500 BID; continue solumedrol ***** XX/XX/XX-XX/XX/XX,then taper; continue Tocilizumab ***** PRN for CRS; Abx as below | ≤ 10 |
| continue keppra 500 BID - continue solumedrol ***** XX/XX/XX-XX/XX/XX,then taper - continue Tocilizumab ***** PRN for CRS | ≤ 10 |
| continue keppra 500 BID - continue solumedrol ***** XX/XX/XX-XX/XX/XX,then taper - continue Tocilizumab ***** PRN for CRS - continue Abx as below | ≤ 10 |
