# Supplementary material for: Extracting adverse event nature, severity, timelines and resulting interventions from clinical notes of patients receiving CAR-T cell therapy using large language models
Source: PLOS Digit Health. 2026 Aug 27;5(8):e0001426. doi: 10.1371/journal.pdig.0001426 (PMC13521353; doi:10.1371/journal.pdig.0001426)
Supplement: Supplementary file 22 [file pdig.0001426.s024.docx]

S21 Table All Clinical Intervention for Grade 4 ICANS

| **Clinical intervention** | **Frequency (patients)** |
| --- | --- |
| Appreciate Neurology consultation | ≤ 10 |
| Continue Solumedrol 1gram IV daily x3 days (XX/XX/XX-XX/XX/XX) given highest c/f neurotoxicity,plan to taper to 500mg IV on XX/XX/XX,and 250mg IV on XX/XX/XX,then off | ≤ 10 |
| S/p Keppra load with 3grams IV x1 dose on XX/XX/XX,and increase standing Keppra to 1gram BID | ≤ 10 |
| S/p Solumedrol 1gram IV daily x3 days (XX/XX/XX-XX/XX/XX) given highest c/f neurotoxicity,now on taper to 500mg IV on XX/XX/XX,and 250mg IV on XX/XX/XX,then off | ≤ 10 |
| S/p Solumedrol 1gram IV daily x3 days (XX/XX/XX-XX/XX/XX) given highest c/f neurotoxicity,now on taper to 500mg IV on XX/XX/XX,and 250mg IV on XX/XX/XX,then off - Appreciate Neurology consultation - S/p Keppra load with 3grams IV x1 dose on XX/XX/XX,and increase standing Keppra to 1gram BID - OK to deescalate abx and defer LP - TTE,tele for stroke w/u negative - Delirium precautions - PT/OT/ST eval,need ARU placement. ***** send Covid tomorrow AM,with goal to discharge on XX/XX/XX. | ≤ 10 |
| Solumedrol 1gram IV daily x3 days (XX/XX/XX-XX/XX/XX),now on taper to 500mg IV on XX/XX/XX,and 250mg IV on XX/XX/XX,then off | ≤ 10 |
| Started on ***** 20mg IV q6hours without improvement in symptoms. Neurology consulted,and felt EEG could be c/w seizures causing *****'s paralysis. | ≤ 10 |
| Started on ***** 20mg IV q6hours without improvement in symptoms. Neurology consulted,and felt EEG could be c/w seizures causing *****'s paralysis. He was loaded with Keppra. Steroid dosing increased to Solumedrol 1gram IV daily x3 days. | ≤ 10 |
| Treatment with steroids (dex),tapered off by XX/XX/XX | ≤ 10 |
