# Supplementary material for: Extracting adverse event nature, severity, timelines and resulting interventions from clinical notes of patients receiving CAR-T cell therapy using large language models
Source: PLOS Digit Health. 2026 Aug 27;5(8):e0001426. doi: 10.1371/journal.pdig.0001426 (PMC13521353; doi:10.1371/journal.pdig.0001426)
Supplement: Supplementary file 23 [file pdig.0001426.s025.docx]

Dear Editors and Reviewers,

We thank both reviewers for the thoughtful, detailed review. We appreciate the constructive feedback and have made substantial revisions to address these points.

In response, we reframed and justified the 50‑note manual review as a feasibility assessment and explicitly acknowledged its limitations. We tempered claims throughout to emphasize that this study supports further evaluation of supervised extraction and that all subgroup-level findings, particularly for prespecified and non-prespecified AEs, should be regarded as hypothesis generating. We added the full annotation guide and clarified the independent dual‑review process clarifying that we aimed to quantify independent agreement rather than generate consensus labels and explaining that we did not formally resolve disagreements. We conducted a sensitivity analysis excluding lab‑based cytopenias and expanded discussion of discrepancy patterns and IRR. We clarified that denominators are prompt‑specific and explained the rationale for extracting explicitly documented “grade 0” for CRS/ICANS. We detailed token/context handling, the rationale for restricting to progress notes. We strengthened reproducibility by providing prompts and the annotation guide in the supplement and a sanitized GitHub repository with analytic code. We performed comprehensive language edits for grammatical and scientific clarity.

Please find our detailed answers to reviewers’ comments below in blue , with line numbers corresponding to the unmarked version of the manuscript.

Reviewer #1: This manuscript addresses an important and clinically relevant problem, extracting adverse event information from unstructured clinical notes in patients receiving CAR-T therapy. The topic is timely, and the use of large language models for this purpose fits well within digital health research. I also appreciate that the study evaluates several extraction targets, including adverse event presence, dates, grades, and interventions, and that it examines CRS and ICANS separately, since these are key toxicities in CAR-T care.

The strongest part of the paper is the focused analysis of CRS and ICANS. The model appears to perform reasonably well in identifying the presence and grade of these toxicities, and this is the most convincing contribution of the study. The authors also note that performance is less consistent for broader adverse event extraction, and I think that is an important point that should remain central to the interpretation of the study.

We thank the reviewer for their thoughtful and detailed review. We appreciate the constructive feedback.

My main concern is the limited validation sample. Although the overall cohort includes 293 patients and 4,762 retained progress notes, the model evaluation is based on manual review of only 50 notes from 47 patients. That is a fairly small sample for assessing several extraction tasks across prespecified adverse events, non-prespecified adverse events, dates, grades, and interventions. The manuscript would benefit from a clearer justification for this sample size, along with a more direct discussion of the uncertainty this creates in the reported performance measures. Claims that go beyond this level of validation should also be softened.

We thank the reviewer for this important point. Our goal was to assess whether the approach shows sufficient promise to justify larger-scale validation rather than to establish definitive performance estimates. We have added details to the Methods section to justify the sample size and to clarify that the manual review was designed as a feasibility assessment rather than a fully powered validation study:

**- lines 202–213**: “A sample of notes was randomly selected for manual evaluation of LLM performance. It was designed as a feasibility evaluation to assess whether a zero-shot LLM could recover key adverse event elements from routine notes. We selected 50 progress notes at random from the eligible set, these sampled notes represented 47 different patients and enabled independent double evaluation by two clinical reviewers within practical time constraints. While each note generated multiple element-level assessments across the four prompts (AE presence, date, grade for CRS/ICANS, and interventions), these observations were not fully independent, and therefore do not eliminate the uncertainty associated with the modest note-level sample size. Our intent was not to produce narrow confidence bounds but to obtain point estimates and to identify tasks where model behavior appeared reliable enough at the zero-shot step. No prompt engineering was performed on the validation notes.”

We have also expanded the Limitations section to explicitly discuss the uncertainty introduced by the small validation set:

**- lines 441–451**: “A key limitation is the small manual review sample relative to the number of extraction tasks assessed. The sample is not powered to provide narrow estimates of model performance and estimates could vary with small changes in counts. In addition, multiple elements within one note were not independent and one note could include content copied forward from previous records or notes without explicit formatting, which can further complicate interpretation and manual assessment of the extracted adverse events. For these reasons, we interpreted performance estimates as preliminary and placed greater emphasis on the qualitative pattern of results (notably, higher point estimates for CRS/ICANS presence and grade). Therefore, all subgroup-level findings, particularly for prespecified and non-prespecified AEs, should be regarded as hypothesis generating.”

A second concern is the variability in inter-rater reliability. Agreement appears strong for some CRS and ICANS outputs, but noticeably weaker for broader adverse event extraction tasks. This suggests that some target labels may be difficult to define consistently from free-text notes. The manuscript would be stronger with a clearer description of the annotation process, the instructions given to reviewers, and whether disagreements were formally resolved. It would also help to discuss whether the lower agreement mainly reflects ambiguity in the notes themselves, ambiguity in the annotation framework, or both.

We would like to thank the reviewer. We have added the annotation guide that was followed by both reviewers to the supplementary materials. Furthermore, we expanded the methods section to describe the annotation process and independence of reviewers, and how disagreements were handled.

**l-224 -230**: “An annotation guide was written by JG and provided to AS to ensure that both evaluators followed a similar evaluation process (Supplementary Material). After reviewing the guide, the reviewers independently assessed the veracity of the model outputs. The reviewers remained independent and did not share information or formally resolve disagreements for the IRR analysis, because the goal was to quantify independent agreement rather than generate consensus labels.”

In addition, to address the variability in IRR for broader AE extraction, we added a sensitivity analysis that recalculates IRR after excluding cytopenias primarily identifiable via labs (anemia, thrombocytopenia, neutropenia, leukopenia). The rationale, approach, and findings are now described in the Methods and Results.

**l-233-236**: “Among the prespecified adverse events, four could be identified from either or both free text or blood count laboratory values: anemia, thrombocytopenia, neutropenia, and leukopenia. Given the complexity and variability in how these are reported in clinical notes, we conducted a sensitivity analysis and calculated IRR for the prespecified adverse events after excluding these four.”

**l-282 – 293**: “Of the 264 inter-reviewer discrepancies, 16 (6.1%) involved thrombocytopenia, 14 (5.3%) involved anemia within the prespecified AE prompt. False negative model outputs -- defined as instances in which the presence of an AE, date, or clinical intervention was identified by an evaluator but not the LLM -- were also common discrepancies (3.8% each for AE presence and date in the non-prespecified event prompt; 2.3% each for AE presence and date in the prespecified event prompt). Disagreements on CRS/ICANS presence were infrequent (CRS yes: 1.5%; ICANS yes: 1.1%), consistent with the stronger kappas and higher point estimates we observed for these tasks. Date-related disagreements included many single-date mismatches at low frequency. The sensitivity analysis excluding lab-based AEs (Anemia, Thrombocytopenia, Neutropenia, Leukopenia) in the prespecified event prompt showed a moderate IRR for nature (0.63) and lower agreement for date (0.56) and clinical intervention (0.51).”

We also discussed the limitations of the note sample of notes and discrepancies patterns in the Discussion

**l-451-470**: “Another limitation is the variability in inter‑rater reliability (IRR), and the discrepancy patterns help localize where performance and agreement were most fragile. First, temporality was difficult to adjudicate when notes contained multiple absolute dates, relative day counts, or carry‑forward text. Evaluators assessed plain‑text exports without the usual EHR sectioning or time stamps, which further impeded determining AE occurrence relative to infusion. Second, prespecified AEs that can be inferred from both laboratory values and narrative (anemia, thrombocytopenia, neutropenia, leukopenia) could have been documented inconsistently across text and labs and were sometimes historical or resolving; clinicians and evaluators may also have applied different thresholds. In our sensitivity analysis excluding these lab‑based cytopenias, IRR improved for AE nature, supporting this interpretation, though agreement for dates and interventions remained lower. Third, AE and intervention mentions were highly heterogeneous; although causality and imputation were not requested, implicit clinical reasoning about whether an event was CAR‑T–related may have influenced extraction and evaluators’ review which also may reflect differences in the evaluators’ training backgrounds. Fourth, de‑identification placeholders (*****), abbreviations, and copy‑forward practices sometimes obscured extraction, evaluation, or whether text reflected a plan, prophylaxis, or an administered therapy, further increasing variability in IRR. Taken together, these factors suggest that weaker Kappas for prespecified and non-prespecified AEs reflect documentation ambiguity and annotation-framework complexity at least as much as model limitations.”

I also think the discussion should be more careful when referring to implementation. The conclusion moves toward integration into EHR workflows, safety monitoring, and real-world evidence pipelines. While the findings are encouraging for certain tasks, especially CRS and ICANS extraction, the broader adverse event results remain mixed, the validation sample is small, and the work comes from a single institution using one specific model version. The paper should make a clearer distinction between a promising early study and a system that is ready for operational use.

We have made our statements more cautious in:

- **Abstract l-43-46: “**While cautious use of LLMs is warranted due to the variable performance observed in this study, these results support further evaluation of supervised CRS/ICANS extraction in controlled EHR-based research or pilot settings, with monitoring and prospective validation.”

**- Author summary l-58-62**: “Since many notes did not state severity explicitly, the model sometimes could not assign a grade, and the small manually reviewed sample means results on performance are preliminary. We recommend cautious, supervised use focused on higher-performing tasks (detecting and grading CRS and neurotoxicity), with continued validation before broader deployment in electronic records.”

- **Discussion:l-398-401**: These results encourage physicians and researchers to conduct further research using these tools in CAR-T monitoring, clinical trials, and real-world evidence research, while emphasizing the need for careful supervision and improving and standardizing performance.
**l-436-438**: “These findings support further supervised evaluation and validation of LLM-driven extraction tools in clinical and research settings to complement existing manual or structured data sources within oncology practice and research”
**l-502-504**: “Moreover, we envision evaluating this method in other healthcare systems and with additional model versions to better assess generalizability, robustness, and potential future applications.”

**- Conclusion l-513-516**: “These results support further prospective evaluation of supervised LLM-based extraction of CRS and ICANS grading in EHR-based research or pilot settings, accompanied by prospective monitoring and expanded validation. Broader AE extraction should remain exploratory until supported by larger, more diverse manual evaluations.”

Another issue is data availability. The manuscript states that the underlying data are restricted and not available upon request. I understand the sensitivity of clinical text data, but the authors should clarify whether any materials that support reproducibility can still be shared, such as prompts, abstraction guidelines, code, evaluation scripts, aggregate outputs, or other derived resources. As written, the statement may raise concerns in relation to the journal’s data sharing expectations.

We understand the importance of sharing materials to support reproducibility. We have included the annotation guidelines and prompts in the supplementary materials. However, we cannot share any clinical text data, note-level annotations, or local evaluation scripts that contain or depend on patient‑level data, institutional database structure, or clinical-note processing workflows. We have provided a GitHub repository with sanitized analytic code that has been reviewed to ensure it contains no sensitive information from clinical notes or patient-level data.

We have detailed the availability of all materials in the Data Availability Statement **(l-561-570):**

“The annotation guidelines and prompts are provided in the Supplementary Materials. Code to reproduce the analyses is available at GitHub [https://github.com/jordan-guillot/Adverse-Event-Extraction-from-CAR-T-Clinical-Notes-Using-Large-Language-Models] and has been reviewed to ensure it contains no sensitive information from clinical notes or patient-level data. Due to the sensitivity of clinical text data and institutional/IRB restrictions, the underlying de-identified patient-level data, including clinical notes and note-level annotations, are not publicly available and cannot be shared outside our institution, even upon request. Aggregate results and summary statistics are included in the manuscript and Supplementary Materials, and sanitized analytic code is available where feasible and consistent with institutional policies.”

The methods section would also benefit from more detail in a few places. In particular, the paper should explain more clearly how long notes were handled in relation to model context limits, how truncation may have affected extraction quality, why only progress notes were included, and whether excluding other note types may have removed clinically relevant adverse event information. It would also be useful to discuss the decision to treat each infusion as an independent episode and whether repeated episodes from the same patient could have affected the descriptive findings.

We thank the reviewer for this helpful suggestion. We would like to clarify that we did not truncate individual notes; instead, we restricted the overall sample by excluding notes based on length or type. To avoid confusion, we added two paragraphs to the Methods section describing how notes were handled in relation to model context limits.

**-l-154–161** :” We tokenized all candidate notes using tiktoken and compared token counts to the GPT 4 0314 context window (8k tokens). To ensure the full note plus prompt (described below) fit and allow headroom for model output, we set an operational cap of 6,000 input tokens per note. Notes with fewer than 71 tokens (bottom quartile) were excluded due to limited clinical content, and notes with more than 6,000 tokens were excluded to avoid exceeding model context. No truncation or chunking was performed in the primary analysis; each retained note was submitted in full.”

**- l-174–179:** “For each retained note, we constructed the user message as ‘Clinical note: “”“”“”’ + task and computed a conservative output-token budget to keep the combined input+output within the 8,192-token context window. Because the 6,000-token input cap was enforced upstream with tiktoken, input side overflows were not expected. If the code returned a token-budget error, we reduced the max tokens parameter and retried.”

We added the rationale for selecting only progress notes to the Methods section **(lines 163–169):**
“We restricted the analysis to progress notes because these documents consistently synthesize interval symptoms, assessment/plans, and bedside interventions relevant to AE onset, grade rationales, and management. To reduce heterogeneity in narrative structure and to ensure cost-effective analysis and evaluation, we excluded other types of notes (e.g., nursing documentation, consult notes, procedure notes, imaging reports, discharge summaries). This design choice prioritized meaningful clinical extraction for zero-shot prompting.”

We also provided the frequencies of each note type in the Supplementary Materials and expanded the Discussion’s Limitations section to address the restriction to progress notes **(lines 478–488)**:

“Limiting the analysis to progress notes optimized our use of resources and likely reduced heterogeneity, but it may have excluded clinically meaningful AE signals captured elsewhere (e.g., nursing notes for vitals and supportive care, consults for subspecialty assessments, ED notes for early symptoms, and discharge summaries for consolidated course/grade rationales). As a result, some AE dates or interventions may have been missed when they were documented exclusively in non-progress note types. An important next step would be to extend extraction to targeted additional note types. Furthermore, because only length-eligible progress notes were analyzed, the retained note set represented a selected subset of all available post-infusion documentation, which may have introduced selection bias if adverse events, interventions, or severity details were preferentially documented in excluded note types or in notes outside the token-length range.”

In the Methods, we clarified that although each infusion was considered a separate episode, the primary descriptive statistics were aggregated at the patient level **(l-150-153):**
“If a patient received multiple CAR-T infusions at UCSF, each infusion was counted as a separate, independent episode. However, the primary description of the cohort and AE frequencies were aggregated at the patient level.”

In the Results, we noted that only one patient received two separate CAR‑T infusions. Because this involved a single patient and the infusions were approximately 10 months apart, we expect any impact on the descriptive findings to be minimal **(lines 245–248)**:
“Among these patients, only one received CAR-T therapy twice, in two separate instances approximately 10 months apart. Due to the rarity of this situation and the time difference between instances, we expect any impact on the analysis descriptive findings to be minimal.”

Finally, although the manuscript is understandable overall, it would benefit from careful language editing. There are a number of grammatical and phrasing issues throughout the paper that should be corrected during revision. For example, there are subject-verb agreement problems and several awkward sentences in the introduction, discussion, and conclusion.

We performed comprehensive language edits for grammatical and scientific clarity.

In summary, this is a relevant and promising study with a meaningful clinical use case and encouraging results for CRS and ICANS extraction. However, the manuscript would be stronger if it more directly addressed the limitations of the validation design, annotation reliability, reproducibility, and the framing of clinical implementation. I recommend major revision.

Reviewer #2:

We thank the reviewer for the careful reading and constructive suggestions.

-statement that “In 2023, six CAR T-cell therapies were approved” may be temporally imprecise and could be interpreted as all six therapies receiving approval in 2023. Consider revising to clarify that six FDA-approved CAR T-cell therapies were available as of 2023.

The text has been updated to “were available on the US market” **l-72:**

-sentence “CAR-T cell therapy are associated…” contains a grammatical inconsistency between singular and plural forms. Please revise for grammatical and scientific clarity.
-sentence describing ICANS contains a sentence fragment (“…ICANS (10). which can occur frequently…”). This should be revised to improve readability and maintain formal scientific style.

The text has been updated **(l-75 - 79):**

“Despite their considerable therapeutic potential, CAR-T cell therapies are associated with frequent and clinically significant adverse events (AEs) (1–4), including cytokine release syndrome (CRS) (7–9) and immune effector cell-associated neurotoxicity syndrome (ICANS) (10), which are among the most important toxicities observed after CAR-T therapy.”

The description of CRS as “severe and potentially life-threatening” may overgeneralize the clinical spectrum of CRS, which ranges from mild to severe presentations. Consider more balanced wording such as “potentially severe and life-threatening.”

The text has been updated **(l-81-85)**:

“CRS is a potentially severe and potentially life-threatening AE characterized by an excessive immune response triggered by the activated CAR-T cells releasing large amounts of cytokines into the bloodstream (11). Symptoms of CRS can range from mild constitutional symptoms to severe inflammatory responses, which can lead to multi-organ dysfunction or even death if not managed promptly and effectively (12).”

There appears to be an internal inconsistency in the note-filtering methodology. The Methods section states that notes with fewer than 71 or greater than 6000 tokens were excluded, whereas the Results section describes retaining notes with more than 71 tokens or fewer than 6000 tokens; the latter should likely use an “and” condition rather than “or.”

The text has been updated in the result section **(l – 256 - 258)**

“Specifically, after filtering by note length (i.e., retaining only notes with more than 71 tokens and fewer than 6000 tokens), 18,808 notes remained, of which 4,762 (25.3%) were progress notes retained for analysis. (Table 1).”

Manual validation was performed on only 50 notes from 47 patients, which is a relatively small evaluation dataset given the breadth of extraction tasks assessed. This limitation should be more explicitly acknowledged when interpreting performance estimates and subgroup-specific conclusions.

We thank the reviewer for this important point. Our goal was to assess whether the approach shows sufficient promise to justify larger-scale validation rather than to establish definitive performance estimates. We have added details to the Methods section to justify the sample size and to clarify that the manual review was designed as a feasibility assessment rather than a fully powered validation study:

**- lines 202–213**: “A sample of notes was randomly selected for manual evaluation of LLM performance. It was designed as a feasibility evaluation to assess whether a zero-shot LLM could recover key adverse event elements from routine notes. We selected 50 progress notes at random from the eligible set, these sampled notes represented 47 different patients and enabled independent double evaluation by two clinical reviewers within practical time constraints. While each note generated multiple element-level assessments across the four prompts (AE presence, date, grade for CRS/ICANS, and interventions), these observations were not fully independent, and therefore do not eliminate the uncertainty associated with the modest note-level sample size. Our intent was not to produce narrow confidence bounds but to obtain point estimates and to identify tasks where model behavior appeared reliable enough at the zero-shot step. No prompt engineering was performed on the validation notes.”

We have also expanded the Limitations section to explicitly discuss the uncertainty introduced by the small validation set:

**- lines 441–451**: “A key limitation is the small manual review sample relative to the number of extraction tasks assessed. The sample is not powered to provide narrow estimates of model performance and estimates could vary with small changes in counts. In addition, multiple elements within one note were not independent and one note could include content copied forward from previous records or notes without explicit formatting, which can further complicate interpretation and manual assessment of the extracted adverse events. For these reasons, we interpreted performance estimates as preliminary and placed greater emphasis on the qualitative pattern of results (notably, higher point estimates for CRS/ICANS presence and grade). Therefore, all subgroup-level findings, particularly for prespecified and non-prespecified AEs, should be regarded as hypothesis generating.”

Reported denominators for CRS and ICANS differ across extraction strategies (e.g., CRS 235 vs 239 patients; ICANS 102 vs 132 patients). Although these discrepancies may reflect prompt-specific extraction behavior, additional clarification is needed to avoid confusion regarding cohort definitions and outcome ascertainment.

We would like to confirm that these numbers are correct. Denominator differences arise because each prompt was executed independently, and patient‑level counts were derived separately for each prompt. We added details in the methods **(l-192-195)**:

“Each of the four prompts was executed independently on the same set of progress notes. AE and Clinical interventions counts were derived separately per prompt without cross prompt reconciliation. Therefore, AE and clinical intervention counts are prompt specific and may differ across prompts.”

We also highlighted this in the results **(l-322-324 and l-345-347)**: “Because each prompt was run independently and outputs were not reconciled across prompts, the patient-level CRS/ICANS count from the CRS/ICANS-specific prompt differed from the CRS/ICANS count extracted by the prespecified AE prompt.”

- use of “Grade 0 CRS” and “Grade 0 ICANS” is conceptually unclear, as Grade 0 generally reflects absence of toxicity rather than an adverse event category. Reporting Grade 0 within cohorts already described as having CRS or ICANS may therefore create interpretative ambiguity and should be clarified.

We thank the reviewer for raising this important point. We agree that Grade 0 reflects absence of active CRS or ICANS rather than an adverse event category. However, because clinicians frequently document “CRS grade 0” or “ICANS grade 0” during post-CAR-T monitoring, we instructed the model to extract grade 0 when it is explicitly documented, so that these clinically meaningful monitoring statements could be preserved rather than interpreted by the model.

We added this rationale in the Methods section **(lines 188–192)**:

“Clinicians frequently document ‘CRS grade 0’ and ‘ICANS grade 0’ in the clinical notes during daily monitoring to indicate no active toxicity. We therefore instructed the model to extract ‘grade 0’ when explicitly documented, even though it reflects absence of the event. In all analyses, grade 0 was treated as an explicitly documented grade category and interpreted as absence of active CRS/ICANS, rather than as an adverse event.”.

-conclusion may overstate implementation readiness by suggesting integration into EHR workflows despite the use of zero-shot prompting, single-center data, limited manual validation, and variable performance across broader AE categories. -interpretation would be strengthened by framing the findings as supporting further validation and supervised evaluation of CRS/ICANS extraction workflows rather than immediate integration.

We have made our statements more cautious in:

**Abstract l-43-46: “**While cautious use of LLMs is warranted due to the variable performance observed in this study, these results support further evaluation of supervised CRS/ICANS extraction in controlled EHR-based research or pilot settings, with monitoring and prospective validation.”

**- Author summary l-58-62**: “Since many notes did not state severity explicitly, the model sometimes could not assign a grade, and the small manually reviewed sample means results on performance are preliminary. We recommend cautious, supervised use focused on higher-performing tasks (detecting and grading CRS and neurotoxicity), with continued validation before broader deployment in electronic records.”

- **Discussion:l-398-401**: These results encourage physicians and researchers to conduct further research using these tools in CAR-T monitoring, clinical trials, and real-world evidence research, while emphasizing the need for careful supervision and improving and standardizing performance.
**l-436-438**: “These findings support further supervised evaluation and validation of LLM-driven extraction tools in clinical and research settings to complement existing manual or structured data sources within oncology practice and research”
**l-502-504**: “Moreover, we envision evaluating this method in other healthcare systems and with additional model versions to better assess generalizability, robustness, and potential future applications.”

**- Conclusion l-513-516**: “These results support further prospective evaluation of supervised LLM-based extraction of CRS and ICANS grading in EHR-based research or pilot settings, accompanied by prospective monitoring and expanded validation. Broader AE extraction should remain exploratory until supported by larger, more diverse manual evaluations.”
