# Supplementary material for: Extracting adverse event nature, severity, timelines and resulting interventions from clinical notes of patients receiving CAR-T cell therapy using large language models
Source: PLOS Digit Health. 2026 Aug 27;5(8):e0001426. doi: 10.1371/journal.pdig.0001426 (PMC13521353; doi:10.1371/journal.pdig.0001426)
Supplement: Peer review history

# Peer review: Decision Letter 0

**DOI:** 10.1371/journal.pdig.0001426.r001

**Authors:** Louise A C Millard, Wisit Cheungpasitporn

25 May 2026

Response to Reviewers Revised Manuscript with Track Changes Manuscript **Journal Requirements:**

**Additional Editor Comments (if provided):** **Reviewers' Comments:**

**Comments to the Author**

1. Does this manuscript meet PLOS Digital Health’s publication criteria?

Reviewer #1: Yes

Reviewer #2: Yes

**********

2. Has the statistical analysis been performed appropriately and rigorously?

Reviewer #1: Yes

Reviewer #2: Yes

**********

3. Have the authors made all data underlying the findings in their manuscript fully available (please refer to the Data Availability Statement at the start of the manuscript PDF file)?

The PLOS Data policy

Reviewer #1: No

Reviewer #2: Yes

**********

4. Is the manuscript presented in an intelligible fashion and written in standard English?

Reviewer #1: Yes

Reviewer #2: Yes

**********

Reviewer #1: This manuscript addresses an important and clinically relevant problem, extracting adverse event information from unstructured clinical notes in patients receiving CAR-T therapy. The topic is timely, and the use of large language models for this purpose fits well within digital health research. I also appreciate that the study evaluates several extraction targets, including adverse event presence, dates, grades, and interventions, and that it examines CRS and ICANS separately, since these are key toxicities in CAR-T care.

The strongest part of the paper is the focused analysis of CRS and ICANS. The model appears to perform reasonably well in identifying the presence and grade of these toxicities, and this is the most convincing contribution of the study. The authors also note that performance is less consistent for broader adverse event extraction, and I think that is an important point that should remain central to the interpretation of the study.

My main concern is the limited validation sample. Although the overall cohort includes 293 patients and 4,762 retained progress notes, the model evaluation is based on manual review of only 50 notes from 47 patients. That is a fairly small sample for assessing several extraction tasks across prespecified adverse events, non-prespecified adverse events, dates, grades, and interventions. The manuscript would benefit from a clearer justification for this sample size, along with a more direct discussion of the uncertainty this creates in the reported performance measures. Claims that go beyond this level of validation should also be softened.

A second concern is the variability in inter-rater reliability. Agreement appears strong for some CRS and ICANS outputs, but noticeably weaker for broader adverse event extraction tasks. This suggests that some target labels may be difficult to define consistently from free-text notes. The manuscript would be stronger with a clearer description of the annotation process, the instructions given to reviewers, and whether disagreements were formally resolved. It would also help to discuss whether the lower agreement mainly reflects ambiguity in the notes themselves, ambiguity in the annotation framework, or both.

I also think the discussion should be more careful when referring to implementation. The conclusion moves toward integration into EHR workflows, safety monitoring, and real-world evidence pipelines. While the findings are encouraging for certain tasks, especially CRS and ICANS extraction, the broader adverse event results remain mixed, the validation sample is small, and the work comes from a single institution using one specific model version. The paper should make a clearer distinction between a promising early study and a system that is ready for operational use.

Another issue is data availability. The manuscript states that the underlying data are restricted and not available upon request. I understand the sensitivity of clinical text data, but the authors should clarify whether any materials that support reproducibility can still be shared, such as prompts, abstraction guidelines, code, evaluation scripts, aggregate outputs, or other derived resources. As written, the statement may raise concerns in relation to the journal’s data sharing expectations.

The methods section would also benefit from more detail in a few places. In particular, the paper should explain more clearly how long notes were handled in relation to model context limits, how truncation may have affected extraction quality, why only progress notes were included, and whether excluding other note types may have removed clinically relevant adverse event information. It would also be useful to discuss the decision to treat each infusion as an independent episode and whether repeated episodes from the same patient could have affected the descriptive findings.

Finally, although the manuscript is understandable overall, it would benefit from careful language editing. There are a number of grammatical and phrasing issues throughout the paper that should be corrected during revision. For example, there are subject-verb agreement problems and several awkward sentences in the introduction, discussion, and conclusion.

In summary, this is a relevant and promising study with a meaningful clinical use case and encouraging results for CRS and ICANS extraction. However, the manuscript would be stronger if it more directly addressed the limitations of the validation design, annotation reliability, reproducibility, and the framing of clinical implementation. I recommend major revision.

Reviewer #2: -statement that “In 2023, six CAR T-cell therapies were approved” may be temporally imprecise and could be interpreted as all six therapies receiving approval in 2023. Consider revising to clarify that six FDA-approved CAR T-cell therapies were available as of 2023.

-sentence “CAR-T cell therapy are associated…” contains a grammatical inconsistency between singular and plural forms. Please revise for grammatical and scientific clarity.

-sentence describing ICANS contains a sentence fragment (“…ICANS (10). which can occur frequently…”). This should be revised to improve readability and maintain formal scientific style.

The description of CRS as “severe and potentially life-threatening” may overgeneralize the clinical spectrum of CRS, which ranges from mild to severe presentations. Consider more balanced wording such as “potentially severe and life-threatening.”

There appears to be an internal inconsistency in the note-filtering methodology. The Methods section states that notes with fewer than 71 or greater than 6000 tokens were excluded, whereas the Results section describes retaining notes with more than 71 tokens or fewer than 6000 tokens; the latter should likely use an “and” condition rather than “or.”

Manual validation was performed on only 50 notes from 47 patients, which is a relatively small evaluation dataset given the breadth of extraction tasks assessed. This limitation should be more explicitly acknowledged when interpreting performance estimates and subgroup-specific conclusions.

Reported denominators for CRS and ICANS differ across extraction strategies (e.g., CRS 235 vs 239 patients; ICANS 102 vs 132 patients). Although these discrepancies may reflect prompt-specific extraction behavior, additional clarification is needed to avoid confusion regarding cohort definitions and outcome ascertainment.

- use of “Grade 0 CRS” and “Grade 0 ICANS” is conceptually unclear, as Grade 0 generally reflects absence of toxicity rather than an adverse event category. Reporting Grade 0 within cohorts already described as having CRS or ICANS may therefore create interpretative ambiguity and should be clarified.

-conclusion may overstate implementation readiness by suggesting integration into EHR workflows despite the use of zero-shot prompting, single-center data, limited manual validation, and variable performance across broader AE categories. -interpretation would be strengthened by framing the findings as supporting further validation and supervised evaluation of CRS/ICANS extraction workflows rather than immediate integration.

**********

what does this mean?). If published, this will include your full peer review and any attached files.

**Do you want your identity to be public for this peer review?**  If you choose “no”, your identity will remain anonymous but your review may still be made public.

For information about this choice, including consent withdrawal, please see our Privacy Policy

Reviewer #1: **Yes:**  Sudhakavya Bodapati Venkata

Reviewer #2: No

**********

**Figure resubmission:**

**Reproducibility:**

To enhance the reproducibility of your results, we recommend that authors of applicable studies deposit laboratory protocols in protocols.io, where a protocol can be assigned its own identifier (DOI) such that it can be cited independently in the future. Additionally, PLOS ONE offers an option to publish peer-reviewed clinical study protocols. Read more information on sharing protocols at https://plos.org/protocols?utm_medium=editorial-email&utm_source=authorletters&utm_campaign=protocols

# Author comment: Author response to Decision Letter 1

**DOI:** 10.1371/journal.pdig.0001426.r002

13 Jul 2026

Attachment Submitted filename: Response to Reviewers.docx

# Peer review: Decision Letter 1

**DOI:** 10.1371/journal.pdig.0001426.r003

**Authors:** Louise A C Millard, Wisit Cheungpasitporn

31 Jul 2026

Extracting adverse event nature, severity, timelines and resulting interventions from clinical notes of patients receiving CAR-T therapy using large language models.

PDIG-D-26-00687R1

Dear Dr Guillot,

We are pleased to inform you that your manuscript 'Extracting adverse event nature, severity, timelines and resulting interventions from clinical notes of patients receiving CAR-T therapy using large language models.' has been provisionally accepted for publication in PLOS Digital Health.

Before your manuscript can be formally accepted you will need to complete some formatting changes, which you will receive in a follow-up email from a member of our team.

Please note that your manuscript will not be scheduled for publication until you have made the required changes, so a swift response is appreciated.

IMPORTANT: The editorial review process is now complete. PLOS will only permit corrections to spelling, formatting or significant scientific errors from this point onwards. Requests for major changes, or any which affect the scientific understanding of your work, will cause delays to the publication date of your manuscript.

If your institution or institutions have a press office, please notify them about your upcoming paper to help maximize its impact. If they'll be preparing press materials, please inform our press team as soon as possible -- no later than 48 hours after receiving the formal acceptance. Your manuscript will remain under strict press embargo until 2 pm Eastern Time on the date of publication. For more information, please contact digitalhealth@plos.org.

Thank you again for supporting Open Access publishing; we are looking forward to publishing your work in PLOS Digital Health.

Best regards,

Wisit Cheungpasitporn, MD

Academic Editor

PLOS Digital Health

***********************************************************

**Additional Editor Comments (if provided):**

**Reviewer Comments (if any, and for reference):**

Reviewer's Responses to Questions

**Comments to the Author**

Reviewer #1: All comments have been addressed

Reviewer #2: All comments have been addressed

**********

publication criteria?

Reviewer #1: Yes

Reviewer #2: Yes

**********

3. Has the statistical analysis been performed appropriately and rigorously?

Reviewer #1: Yes

Reviewer #2: Yes

**********

4. Have the authors made all data underlying the findings in their manuscript fully available (please refer to the Data Availability Statement at the start of the manuscript PDF file)?

The PLOS Data policy

Reviewer #1: No

Reviewer #2: Yes

**********

5. Is the manuscript presented in an intelligible fashion and written in standard English?

Reviewer #1: Yes

Reviewer #2: Yes

**********

Reviewer #1: The authors have adequately addressed my previous comments. The revised manuscript now presents the 50-note evaluation appropriately as a viability assessment, provides clearer details on the annotation process and inter-rater reliability, and includes an additional sensitivity analysis. The discussion and conclusion are also more cautious and better aligned with the limits of the study.

The added prompts, annotation guidance, aggregate results, and sanitized analysis code improve reproducibility. I understand that the underlying patient level clinical notes and annotations cannot be shared because of privacy and institutional restrictions. Overall, the manuscript has been substantially improved and is now suitable for publication.

Reviewer #2: all comments have been addressed.

**********

what does this mean?). If published, this will include your full peer review and any attached files.

**Do you want your identity to be public for this peer review?**  If you choose “no”, your identity will remain anonymous but your review may still be made public.

For information about this choice, including consent withdrawal, please see our Privacy Policy

Reviewer #1: **Yes:**  Sudhakavya Bodapati Venkata

Reviewer #2: No

**********
